# Lactase Persistence and Lipid Pathway Selection in the Maasai

**DOI:** 10.1371/journal.pone.0044751

**Published:** 2012-09-28

**Authors:** Kshitij Wagh, Aatish Bhatia, Gabriela Alexe, Anupama Reddy, Vijay Ravikumar, Michael Seiler, Michael Boemo, Ming Yao, Lee Cronk, Asad Naqvi, Shridar Ganesan, Arnold J. Levine, Gyan Bhanot

**Affiliations:** 1 Department of Physics, Rutgers University, Piscataway, New Jersey, United States of America; 2 The Broad Institute, Cambridge, Massachusetts, United States of America; 3 Dana-Farber Cancer Institute, Boston, Massachusetts, United States of America; 4 BioMaPS Institute, Rutgers University, Piscataway, New Jersey, United States of America; 5 Department of Mathematics, Rutgers University, Piscataway, New Jersey, United States of America; 6 The Cancer Institute of New Jersey, New Brunswick, New Jersey, United States of America; 7 Department of Anthropology, Rutgers University, New Brunswick, New Jersey, United States of America; 8 Institute for Advanced Study, Princeton, New Jersey, United States of America; 9 Department of Molecular Biology and Biochemistry, Rutgers University, Piscataway, New Jersey, United States of America; University of Massachusetts, United States of America

## Abstract

The Maasai are a pastoral people in Kenya and Tanzania, whose traditional diet of milk, blood and meat is rich in lactose, fat and cholesterol. In spite of this, they have low levels of blood cholesterol, and seldom suffer from gallstones or cardiac diseases. Field studies in the 1970s suggested that the Maasai have a genetic adaptation for cholesterol homeostasis. Analysis of HapMap 3 data using Fixation Index (Fst) and two metrics of haplotype diversity: the integrated Haplotype Score (iHS) and the Cross Population Extended Haplotype Homozygosity (XP-EHH), identified genomic regions and single nucleotide polymorphisms (SNPs) as strong candidates for recent selection for lactase persistence and cholesterol regulation in 143–156 founder individuals from the Maasai population in Kinyawa, Kenya (MKK). The non-synonmous SNP with the highest genome-wide Fst was the TC polymorphism at rs2241883 in Fatty Acid Binding Protein 1(*FABP1)*, known to reduce low density lipoprotein and tri-glyceride levels in Europeans. The strongest signal identified by all three metrics was a 1.7 Mb region on Chr2q21. This region contains the genes *LCT* (Lactase) and *MCM6* (Minichromosome Maintenance Complex Component) involved in lactase persistence, and the gene *Rab3GAP1* (Rab3 GTPase-activating Protein Catalytic Subunit), which contains polymorphisms associated with total cholesterol levels in a genome-wide association study of >100,000 individuals of European ancestry. Sanger sequencing of DNA from six MKK samples showed that the GC-14010 polymorphism in the *MCM6* gene, known to be associated with lactase persistence in Africans, is segregating in MKK at high frequency (∼58%). The Cytochrome P450 Family 3 Subfamily A (*CYP3A)* cluster of genes, involved in cholesterol metabolism, was identified by Fst and iHS as candidate loci under selection. Overall, our study identified several specific genomic regions under selection in the Maasai which contain polymorphisms in genes associated with lactase persistence and cholesterol regulation.

## Introduction

The Maasai are a pastoralist, Nilotic people living primarily in southern Kenya and northern Tanzania. An economy traditionally based on herds of cattle, sheep, and goats led to a diet rich in lactose, fat, and cholesterol consisting largely of milk, meat, and blood. Although their cholesterol intake is high (600–2000 mg/day), and 66% of their calories come from fat, their total serum cholesterol levels average 135 mg/100 ml [1]–[4]. In comparison, a study consisting of cohorts from seven countries (Croatia, Finland, Greece, Italy, Japan, Netherlands, USA) found that the average dietary cholesterol intakes are 141–612 mg/day and serum cholesterol levels range from 160–266 mg/100 ml [5]. Greenland Eskimos were found to have a high cholesterol consumption of 420–1650 mg/day [6] with average consumption of ∼700 mg/day [7], and were found to have plasma cholesterol levels of 233 mg/100 ml [8]. Although African children generally have lower cholesterol levels (115–137 mg/100 ml for 7–8 year olds) than other populations [9], the fact that adult Maasai have very low cholesterol levels, inspite of a high cholesterol diet, is quite remarkable. The Maasai also have low rates of cholelithiasis (especially cholesterol gallstones), low blood pressure, and low incidence of atherosclerotic coronary artery disease [1]–[3], [10]. Various hypotheses to understand this puzzle have been proposed, such as: “physical fitness and freedom from emotional stress” [10], [11], a “hypo-cholesterolaemic factor” in milk [12] and saponins derived from herbs [13]. However, the hypo-cholesterolaemic factor was never found, and the model of [10], [11] could not explain the low frequencies of heart disease in older Maasai men who lead sedentary lives after age ∼ 24, when their warrior (Murran/Moran) period ends [14], [15].

Additional clues emerged from a controlled experiment [2] on 23 healthy Maasai adults (11 experimental, 12 control) between the ages of 20 and 24 years. All study subjects were fed a basic high calorie, cholesterol-free diet for 8 weeks, including trace amounts (1 micro-curie) of radioactively labeled Cholesterol-4-^14^C. The eleven subjects in the treatment group were fed 2 gm of crystalline cholesterol per day in addition to the basic diet. Blood and fecal samples were collected at the start of the study, weekly for 8 weeks and at the end of 9, 16 and 24 weeks. Using the radioactive tracer to quantitate/normalize the measurements, the data were analyzed to characterize metabolic patterns, namely, the amounts of dietary cholesterol absorbed, synthesized and excreted. The study found that, in spite of the additional 2 gm/day ingestion of cholesterol in the experimental group, there were no significant differences in serum cholesterol, phospholipids, triglyceride levels and lipoprotein patterns between the experimental and control groups. Both groups had identical turnover rates for cholesterol, with no evidence for cholesterol storage in the experimental group. In a similar study in American subjects, Mattson et al [16] found that total serum cholesterol increased linearly with dietary cholesterol with 11.8 mg/100 ml increase for every 100 mg/1000 kcal increase in dietary cholesterol over the range 100–317 mg/1000 kcal. Were this relation to hold in the Maasai, an increase of 66 mg/100 ml total cholesterol levels would be expected in the above experiment, contrary to the observed cholesterol homeostasis. The observed cholesterol homeostasis could not be attributed to a “hypo-cholesterolaemic factor”, or to saponins, which were absent from the Maasai study diet. The authors concluded that “the Maasai have some basically different genetic traits that result in their having superior biologic mechanisms for protection from hypercholesterolemia” [3].

It is widely accepted that there is a strong genetic component in the risk of hypercholesterolemia, atherosclerosis and heart disease [17]–[20]. Typically, genome-wide association studies (GWAS) focus on markers for *increased risk of disease* [21]–[25] and to a lesser extent on protective polymorphisms. Such protective polymorphisms are known to arise as adaptations and can be identified in selection studies. For example, many studies have identified polymorphisms conferring lactase persistence in Northern Europeans, which arose with the advent of cattle breeding [26]. Just as in Europe, pastoralism arose in East Africa around 4,000–10,000 years ago [27] leading to selection for lactase persistence [28]. In the Maasai, pastoralism led to a lactose rich, high fat, high cholesterol diet of milk, meat and blood [4]. It is quite reasonable that, in a time span similar to that which conferred lactase persistence in Europeans, selection pressure in the Maasai from such a diet might result in genetic adaptations against diseases such as hypercholesterolemia and atherosclerosis.

Motivated by this possibility, we performed a genome wide scan for selection in 143–156 founder individuals from the Maasai of Kinyawa, Kenya (MKK) using the HapMap 3 [29] SNP (single nucleotide polymorphism) data to identify genomic regions under recent selection. We also used 90–110 HapMap 3 founder individuals from the Luhya population from Webuye, Kenya (LWK) as a reference group. Three complementary metrics to detect selection were applied: the Fixation Index (Fst) [30], the Cross Population Extended Haplotype Homozygosity (XP-EHH) [31], and the Integrated Haplotype Score (iHS) [32], [33]. Note that the phased data used for iHS and XP-EHH was from HapMap3 Release 2, which has fewer individuals (143 and 90 for MKK and LWK respectively) whereas the data for Fst was from HapMap Release 3, which had more individuals (156 and 110 respectively). Our analysis consistently identified strong, recent selection in genes involved in lipid metabolism and lactase persistence in the Maasai (MKK) samples. Several of the regions under selection in MKK contained specific polymorphisms known to protect against hyperlipidemia in other populations. Sanger sequencing of DNA from six MKK samples showed that the GC-14010 polymorphism in the Minichromosome Maintenance Complex Component (*MCM6)* gene, known to confer adult lactase persistence in East Africans [28], is segregating in the Maasai at a frequency of ∼58%. These results suggest that the regions identified contain polymorphisms that confer lactase persistence and protection from hypercholesterolemia in the Maasai. The wider consequence of our study is that consistent dietary pressure can induce strong selection in complex pathways in a short time (∼150–400 generations).

## Results

### Population Structure

Two of the methods used to detect selection (Fst and XP-EHH) require a genetically similar reference population. A comparison of Fst among HapMap populations shows that the MKK and African-Americans from South-west USA (ASW) have the lowest average Fst (0.0145), followed by MKK and the Luhya in Webuye, Kenya (LWK) (0.017), while Fst between MKK and Yoruba from Nigeria (YRI) is significantly higher (0.027) (Table S6 in [29]). However, a plot of the first two principal components from a PCA analysis of the African populations and Utah residents with Northern and Western European ancestry from the CEPH collection (CEU) (Figure S2, (c) in [29]) shows that the MKK are genetically closer to LWK.

**Figure 1:**
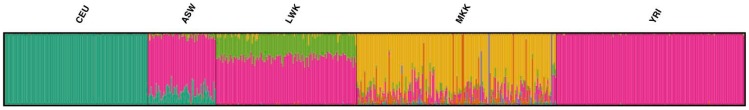
Population structure components for individuals from CEU, ASW, LWK, MKK and YRI. Results from STRUCTURE version 2.3 on genotype data for 12,999 randomly selected SNPs in 578 founder (unrelated) individuals from the CEU, ASW, LWK, MKK and YRI HapMap populations. The no-admixture model showed that the data was best fit by 6 inferred ancestral populations. Each column represents an individual, and the colors indicate the fractions of their genotype attributable to ancestry from each of the 6 ancestral populations.

To understand the degree of admixture in the populations ASW, CEU, LWK, MKK and YRI, we used STRUCTURE [34] on a randomly sampled subset of 12,999 SNPs from the HapMap 3 dataset. Without using any population identification information, STRUCTURE found that the data fits best to 6 ancestral populations (Figure 1, details in Appendix S1). In agreement with [29], [35], the STRUCTURE results show that whereas the CEU and YRI are genetically homogenous, the LWK, ASW and MKK are admixed, with a ∼20% CEU admixture in ASW. The LWK and ASW also have a large admixture with YRI (66% and 76% respectively), while MKK have a smaller admixture with YRI (10%). In addition, the STRUCTURE results indicate that MKK have a 15% admixture with two populations that are not sampled in the HapMap study. We also see a small admixture between MKK and LWK, which is expected, given their geographical proximity. These results are largely consistent with linguistic phylogeny; whereas the Maasai speak a Nilo-Saharan language, the Luhya and the Yoruba speak Niger-Congo languages, also spoken by African ancestors of African Americans [35].

To further quantify the genetic similarity of MKK, LWK, ASW and YRI to the six ancestral populations, we assigned a six component vector to each of these populations, whose coordinates were the fraction of the ancestral components represented in them. A comparison of the cosine similarity of these vectors showed that the largest overlap was between MKK and LWK (0.18), followed by MKK and ASW (0.16). Based on their closer proximity to MKK in the PCA plot, as well as closer cosine similarity, we chose the LWK as the appropriate reference population for the Fst and XP-EHH analysis.

**Table 1:** Top 20 genomic regions identified as selection candidates in MKK using the Fst statistic and clustering.

Chr	Start location	Stop location	Genes in region	Number of HighFst SNPs (empiricalp-value <0.001)	Max Fst within cluster	Max XP-EHH score within cluster
2	135036696	136726567	RAB3GAP1, ZRANB3, DARS, R3HDM1, TMEM163,YSK4, LCT, UBXN4, MCM6, MGAT5, CCNT2	123	0.382	12.202
2	78305622	78500655	-	33	0.311	3.805
12	56402204	56754137	PAN2, OBFC2B, SLC39A5, APOF, STAT2, CS,RNF41, IKZF4, SMARCC2	28	0.283	3.024
3	191929784	191990575	FGF12	13	0.272	5.222
5	115126388	115223035	ATG12, AP3S1	7	0.266	3.870
2	163048404	163152351	IFIH1, FAP	19	0.261	3.108
7	99053816	99436198	ZNF498, CYP3A4, CPSF4, CYP3A7, CYP3A43	17	0.260	3.290
1	12296232	12319994	VPS13D	4	0.253	3.060
22	49978502	50077531	-	4	0.244	3.732
5	32128179	32159329	GOLPH3	5	0.242	3.062
5	14747247	14750823	ANKH	4	0.237	6.800
14	36033703	36201722	RALGAPA1	4	0.221	3.517
2	136917330	136921703	-	2	0.218	8.549
1	198692364	198745866	PTPRC	2	0.212	3.138
2	137580234	137595545	-	4	0.209	4.871
12	111414527	111502280	CUX2	5	0.209	3.393
17	75423198	75431978	SEPT9	3	0.200	5.024
18	66714832	66724690	CCDC102B	4	0.200	5.704
1	74807337	74842787	TNNI3K	3	0.193	3.993
3	185752767	185805993	ETV5	3	0.192	4.569

1,232 SNPs with significant Fst scores (p_B_<8.6E−6, p_E_<0.001) were clustered into contiguous genomic regions of linkage disequilibrium. A cluster was defined as a collection of SNPs in a genomic region where each SNP had genotype R^2^≥0.25 with at least one other SNP in the cluster. Clusters containing a SNP with maximum XP-EHH score >3 were identified as being MKK associated. The 22 top clusters are ranked by the highest Fst value for a SNP pair in a cluster. The complete set of clusters identified by Fst is in Table S2.

### Identifying selection in the Maasai

#### Selection based on Fst

We calculated Fst between MKK (n = 156) and LWK (n = 110) as in [30] for 1,175,055 SNPs common to both populations that passed filters for minor allele frequency, genotyping rate, and consistency with Hardy-Weinberg equilibrium. Statistical significance was assessed using a Bonferroni corrected permutation test p-value p_B_ (Methods, Appendix S2). Within the SNPs that passed this filter, we identified those deviating significantly from neutral evolution using an empirical p-value (p_E_) based on the Fst distribution of inter-genic SNPs. This identified 1,232 SNPs with p_B_<8.6E−6 and p_E_ <0.001 (Table S1) which were either genic or within 50 kb of genes.

**Table 2:** The most significant non-synonymous SNPs under selection in MKK using Fst, with LWK as the reference population.

Rsid of SNP	Chr	Position	Gene	Bonferroni correctedPermutation p-value (pB)	Empirical p-value (pE)using distribution ofnon-coding SNPs	Fst MKK vs LWK	Fst MKK vs YRI	Fst MKK vs ASW
rs2241883	2	88424066	FABP1	1.72E−12	3.13E−05	0.250	0.172	0.152
rs961360	2	136393658	R3HDM1	3.13E−08	3.13E−04	0.199	0.288	0.447
rs6997753	8	142487937	FLJ43860	4.87E−08	3.59E−04	0.194	0.138	0.006
rs531503	7	100377082	ZAN	3.83E−07	5.47E−04	0.182	0.014	0.073
rs17014118	4	89319296	HERC6	4.42E−07	6.06E−04	0.180	0.178	0.045
rs2271586	11	3659993	ART5	4.76E−07	6.06E−04	0.180	0.034	0.004
rs10930046	2	163137983	IFIH1	1.24E−06	6.86E−04	0.176	0.279	0.128
rs1051334	12	71523134	TSPAN8	1.36E−06	6.86E−04	0.176	0.173	0.104
rs10475299	5	5461233	KIAA0947	1.46E−06	6.86E−04	0.175	0.160	0.198
rs1918496	12	56722060	PAN2	3.06E−06	8.17E−04	0.171	0.296	0.074
rs13389745	2	65298657	CEP68	3.84E−06	8.17E−04	0.172	0.115	0.052
rs846266	7	42088222	GLI3	2.54E−06	9.42E−04	0.169	0.150	0.059
rs3813227	2	73651967	ALMS1	6.02E−06	9.82E−04	0.167	0.173	0.034

The most significant non-synonymous SNPs identified as candidates for selection by Fst. The complete list of 1,232 SNPs identified as selection candidates by Fst (p_B_ <8.6E−6 and p_E_ <0.001) is in Table S1.

In a recent selective sweep, many neighboring SNPs may remain linked due to genetic hitchhiking. To identify such regions, we grouped the genome-wide significant SNPs identified by Fst into clusters based on linkage disequilibrium using the criterion that each SNP has genotype R^2^≥0.25 with at least one other SNP in the cluster (Methods, Appendix S2). Each cluster so identified is a candidate for a selective sweep in one of the two populations. To identify the population in which the sweep is most likely to have occurred, we compared the local haplotype diversity in each population using the XP-EHH score [31]. For each cluster identified by Fst, we label it as a selection candidate in MKK if the maximum XP-EHH score of a SNP in the cluster is >3. A positive value for XP-EHH indicates that the MKK carry the longer-range haplotypes. This procedure identified 26 clusters (containing 318 SNPs) as candidate regions for selective sweeps in MKK (Table S2). Nine of these clusters include SNPs that exceed the genome-wide significance threshold for XP-EHH (XP-EHH >4.79580, Bonferroni corrected p<0.05, two-tailed). The most significant genomic regions and non-synonymous SNP candidates under selection in MKK by Fst are listed in Table 1 and Table 2 respectively. Note that the isolated SNPs identified in Table 2 have high Fst with respect to at least two of the three possible reference African populations (ASW, LWK and YRI). This suggests that the results shown there are relatively independent of the reference population.

#### Selection based on his

Recent selective sweeps amplify beneficial mutations and reduce haplotype diversity due to the hitchhiking effect. The Extended Haplotype Homozygosity [32] (EHH) statistic identifies such events *without* using a reference population. EHH(*x*) measures the probability that two randomly selected haplotypes sharing the same allele at a SNP are identical up to genomic distance *x*. At each SNP, we computed the unstandardized Integrated Haplotype Score [33] (iHS), defined as the logarithm of the ratio of the integrated EHH scores for the ancestral allele and the derived allele. Stratifying the data into bins by the derived allele frequency of the SNPs, the scores within each bin were then normalized to have zero mean and unit standard deviation. The iHS statistic is less sensitive to demographic history (e.g. population bottlenecks) and to local differences in recombination rates, because such factors have similar effects on ancestral and derived alleles, and tend to cancel in the ratio [33]. If either allele is under selection, the reduced haplotype diversity around it will tend to increase the absolute value of iHS.

Following the protocols in [33], raw iHS scores for 991,737 SNPs in MKK (n = 143 individuals) that passed filters (minor allele frequency cutoff, consistency with Hardy-Weinberg equilibrium) were binned by derived allele frequency and standard normalized within each bin (details in Methods and Appendix S3). Genomic regions were scored by the fraction of high scoring iHS SNPs (|iHS| >2) using a sliding window of 50 SNPs. The top 0.02% of non-overlapping SNP windows identified 196 regions likely to be under selection (Table S3). These were further grouped on the basis of linkage disequilibrium using the same criterion as for Fst (genotype R^2^≥0.25). The most significant regions identified as candidates for selection in MKK are in Table 3 (the complete list is in Table S4).

**Table 3:** The most significant genomic regions under selection in MKK using iHS.

Chr	Cluster start position (GRCh37)	Cluster end position (GRCh37)	Genes	Max |iHS| in cluster	# of SNPs in cluster with |iHS| >2
2	134221398	137892309	LCT, MGAT5, NCKAP5, DARS, ZRANB3, R3HDM1, TMEM163, RAB3GAP1, THSD7B, CCNT2, YSK4, UBXN4, MCM6	6.339	545
13	30496779	30565298	–	5.234	26
7	20373632	20468718	ITGB8	5.012	45
2	176089888	176422005	–	4.626	69
11	110532348	110663647	ARHGAP20	4.480	36
9	83127968	83382243	–	4.471	59
5	14657062	14753764	FAM105B, ANKH	4.429	23
18	66652846	66765215	CCDC102B	4.402	33
11	34025053	34189564	CAPRIN1, NAT10, ABTB2	4.375	22
2	179421694	179606538	TTN	4.289	28
14	105792959	105907642	PACS2, MTA1	4.228	20
5	108990708	109217428	MAN2A1	4.219	50
9	107973277	108067684	SLC44A1	4.192	34
9	3869844	3919130	GLIS3	4.185	23
7	99053816	99314986	ZNF789, CPSF4, ATP5J2, FAM200A, ZNF655, ZNF498,CYP3A7, ZKSCAN5, CYP3A5	4.120	24
9	13812037	13867306	–	4.066	23
11	75470813	75678647	UVRAG, DGAT2	4.059	48
2	12294875	12366781	–	4.041	24
14	97426813	97505011	–	4.025	24
8	145839058	146082167	COMMD5, LOC100287170, LOC100129596, ARHGAP39,RPL8, ZNF7, ZNF251, ZNF34, LOC100287297, ZNF517	3.955	22

Using a sliding window of 50 SNPs wide, genomic regions were scored for the fraction of SNPs with |iHS|>2. The top 0.02% of non-overlapping windows were identified and merged into genomic clusters based on genotype R^2^ using the same criterion as in Table 1. Clusters are ranked by the maximum |iHS| value in the cluster. Complete lists of genome-wide significant SNPs and regions identified by iHS are in Tables S2a and S2b respectively.

#### Selection based on XP-EHH

The third method used to identify selective sweeps in MKK was the Cross Population Extended Haplotype Homozygosity statistic (XP-EHH) [31]. This statistic compares the EHH profiles for bi-allelic SNPs between two populations. It is defined as the log of the ratio of the integrals of the EHH profiles for a given allele between the two populations (Appendix S4). The comparison between populations normalizes the effects of large-scale variations in recombination rates on haplotype diversity, and has a higher statistical power to detect sweeps that are close to fixation [31].

Using the LWK cohort (n = 90) as the reference population for MKK (n = 143), XP-EHH was calculated for 1,373,755 SNPs that passed various filters (Methods, Appendix S4). Following [31], we assigned p-values using a Gaussian fit after standard normalizing the XP-EHH distribution. SNPs with Bonferroni corrected p-value <0.05 (two-tailed) were chosen as potentially significant candidates for selection. These are listed in Table S5. We also clustered these candidate SNPs (using the genotype R^2^≥0.25 criterion as before) to identify putative regions under selection in MKK (Table S6). The most significant regions thus identified are listed in Table 4.

**Table 4:** The most significant genomic regions under selection in MKK using XP-EHH, with LWK as the reference population.

Chr	Start Position	End Position	Genes	Number of SNPs	Max XP-EHH
2	135058615	137017060	R3HDM1, MGAT5, RAB3GAP1, LCT, DARS, ZRANB3,MCM6, TMEM163, ACMSD, CCNT2, YSK4, UBXN4, CXCR4	572	12.182
5	14681797	14751400	FAM105B, ANKH	25	6.800
18	66712510	66731187	CCDC102B	12	5.587
5	115885282	115922669	SEMA6A	21	5.482
18	66768031	66777543	–	5	5.324
20	4513311	4522535	–	10	5.313
13	104870241	104880533	–	7	5.183
4	64594290	64639661	–	16	5.149
2	134507165	134561145	–	12	5.062
16	75360734	75364940	CFDP1	2	5.040
17	75427551	75428021	SEPT9	2	5.024
3	191943578	191989642	FGF12	10	5.019
11	117610387	117620420	DSCAML1	8	4.989

SNPs with positive genome-wide significant XP-EHH scores (XP-EHH ≥4.796, two-tailed Bonferroni corrected p≤0.05) were grouped into contiguous genomic clusters using genotype R^2^ using the same criterion as in Table 1. Overlapping clusters were merged. Column E lists the number of significant SNPs in each each cluster. Complete lists of genome-wide significant SNPs and clusters identified by XP-EHH are in Tables S3a and S3b.

#### Overlap of high scoring regions

**Figure 2:**
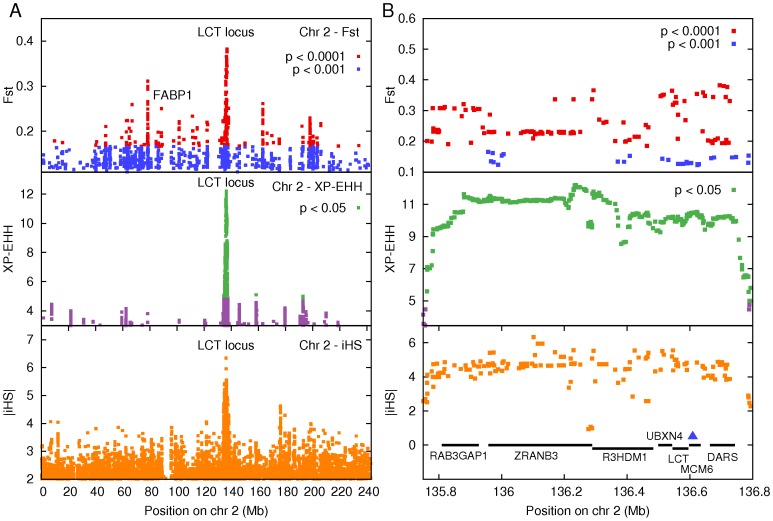
(a) Genome-wide significant scores identifying candidate regions under selection on Chromosome 2. Chromosome wide plot of SNPs with significant scores using Fst (empirical p-value <0.001 and Bonferroni corrected permutation test pB <8.6E−6), iHS (normalized |iHS|>2), and XP-EHH (XP-EHH ≥4.796, two-tailed Bonferroni corrected p≤0.05). The SNPs thus identified were clustered on the basis of linkage disequilibrium to identify contiguous genomic regions that are candidates for selections (Table 1,2,3,4). The locus containing the genes LCT and MCM6 (135–137 Mb) was identified by all three metrics as the top candidate for selection. The non-synonymous TC polymorphism at rs2241883 in the FABP1 gene had most significant genome-wide Fst (Fst = 0.25, pE = 3.13E−5). The MKK samples have a high frequency (∼0.45) of the protective C allele, known to be associated with low cholesterol levels in Europeans (plots for other chromosomes in Appendix S6). **(b) Inset of the LCT locus on Chromosome 2.**An inset of the Fst, iHS and XP-EHH scores for SNPs in the ∼ 1 Mb locus (from 135.8–136.8 Mb) on Chr 2 containing the genes LCT and MCM6. The uniformly high values for all three metrics in this region suggest that this locus has undergone strong selection pressure. The blue marker indicates the position of the lactase associated SNP in MCM6 that we sequenced, which was polymorphic in MKK with frequency pC = 0.58+/−0.14 (68% CI) for the protective C allele.

The metrics we use probe for different signatures of selection, and hence, genomic regions which are identified by more than one metric are more likely to be true positives. Using a concordance between at least two of the metrics, we identified seven genomic regions as strong candidates for selection (Table 5). There was also overlap between the regions identified by our methods and those identified by the International HapMap Consortium for MKK (they used a statistic they call CMS or “Composite of Multiple Signals”) [29]. These regions of concordance are listed in Table S7. Figure 2 shows the results for all three metrics for chromosome 2. The significant selection in a region in Chr2q21 of size ∼ 1.0–1.7 Mb is clearly visible in Figure 2a. Figure 2b shows details of this region which contains a large number of polymorphisms with significant high scores by all three metrics (discussed further below). Similar figures for all chromosomes are shown in Appendix S6.

**Table 5:** Concordant genomic regions identified by at least two of three metrics as candidates for selection in MKK.

Chr	Genomic Extent	Significant by (Method)	Genes in Region	Number of SNPs identified by each Method
2	135058615–136726567	Fst, iHS, XP-EHH	MGAT5, TMEM163, ACMSD, CCNT2, YSK4, RAB3GAP1,ZRANB3, R3HDM1, UBXN4, LCT, MCM6, DARS	Fst: 123, iHS: 545, XP-EHH: 572
3	191943578–191989642	Fst, XP-EHH	FGF12	Fst:13, XP-EHH: 10
5	14747247–14750823	Fst, iHS, XP-EHH	ANKH	Fst: 4, iHS: 23, XP-EHH: 25
5	115885574–115885672	Fst,XP-EHH	SEMA6A	Fst: 2, XP-EHH: 21
7	99053816–99314986	Fst, iHS	ZNF789, CPSF4, ATP5J2, FAM200A, ZNF655,ZNF498, CYP3A7, ZKSCAN5, CYP3A5	Fst: 17, iHS: 24
17	75427551–75428021	Fst, XP-EHH	SEPT9	Fst: 3, XP-EHH: 2
18	66714832–66724690	Fst, iHS, XP-EHH	CCDC102B	Fst: 4, iHS: 33, XP-EHH: 12

Genomic regions identified as genome-wide significant by at least two of the three methods - Fst, iHS and XP-EHH.

### The non-synonymous SNP at rs2241883 in *FABP1* is a Candidate for Selection in Maasai

We found that the non-synonymous SNP with the highest genome-wide significant Fst was rs2241883 in the gene Fatty Acid binding Protein 1, Liver (*FABP1,* alternative name *LFABP*) (Table 2 and Figure 2a). The SNP rs2241883 is a TC non-synonymous transition which encodes a Threonine to Alanine (T94A) change in the protein *LFABP*, which is expressed in liver. The C allele was associated with total tri-glyceride and low density lipoprotein (*LDL*) cholesterol levels in Germans [36], and with Apolipoprotein B (ApoB) levels induced by a high fat diet in French-Canadians [37]. The MKK have high Fst at this SNP, relative to all the other three African populations in Hapmap (Table 2). The allele frequency of the C allele is also highest (0.44) in MKK compared to all other HapMap3 populations (in which the frequency ranges from 0.09–0.32). These results suggest that the rs2241883 polymorphism is under selection in the Maasai.

### Maasai are under Selection in a 1.7 Mb Region on Chr2q21 for Lactase Persistence

The largest cluster under selection in Maasai, identified by all the metrics, was a 1.7 Mb region on Chr2q21 (Figures 2a, 2b, Tables 1,2,3,4). The region includes the Lactase (*LCT)* gene, which encodes the Lactase protein, as well as the gene *MCM6*, which contains intronic regulatory regions for *LCT* [28], [38]–[40]. Specific polymorphisms in these regions are known to confer lactase persistence in Europeans and Africans [28], [38]. Our results are in agreement with other studies that have also shown that this region is under recent, positive selection in the Maasai [28], [31]–[33], [41], [42].

To identify specific polymorphisms for adult lactase persistence in the Maasai, we sequenced DNA from six founder MKK samples (HapMap IDs: NA21367, NA21379, NA21454, NA21519, NA21522, NA21650) at five loci in *MCM6* (G/C-14010, rs41525747, rs4988235, rs41380347 and rs182549), which are known to be associated with lactase persistence in Africans and Europeans [28]. We found that the GC-14010 polymorphism in the MCM6 gene is segregating in these samples (n_GG_ = 1, n_GC_ = 3, n_CC_ = 2). We estimated the frequency of the beneficial (C) allele in the MKK samples to be p_C_ = 0.58+/−0.14 (68% CI from finite size sampling - details in Appendix S5). This is in agreement with Tishkoff et al [28], who showed that this allele is significantly associated with lactase persistence, has significantly reduced haplotype diversity indicative of a selective sweep, and is segregating at high frequency in the Maasai samples from Kenya.

### The Selected Locus on Chr2q21 Contains Polymorphisms Associated with Cholesterol Levels

The selected locus on Chr2q21 contains polymorphisms that have been associated with cholesterol levels in various GWAS studies [43]–[45]. The SNP rs7570971 in *RAB3GAP1*, not found in the HapMap data for the MKK, is associated with total cholesterol levels in a GWAS of >100,000 individuals of European descent [43]. However, the six MKK samples we sequenced were homozygous at this locus in the Maasai for the allele associated with an *increase* in total cholesterol levels in the samples with European descent.

A study in a Finnish cohort identified polymorphisms in *LCT* associated with total cholesterol and Low Density Lipoprotein C (LDL-C) levels [44]. The authors found that the lactase persistence genotype in Finns, as defined by the genotype for SNP rs4988235, was associated with lower cholesterol values. Several SNPs in and around the gene *LCT* were associated with total cholesterol and LDL-C levels, with stronger associations in males than females. This study also found that the G allele at the synonymous SNP rs2304371 in the *LCT* gene was associated with highest LDL-C levels in males. The same SNP was identified by our methods as a selection candidate (Tables S1, S2, S3). However, once again, the major allele in the MKK (frequency 87%) was the one associated with higher LDL-C levels.

### The *CYP3A* Locus is a Candidate for Selection in Maasai

On Chromosome 7, a 261 kb wide region spanning the entire Cytochrome P450 Subfamily 3A (*CYP3A*) locus was identified as a candidate for selection by Fst and iHS (Tables 1, 2). All *CYP* genes in this locus contain SNPs with genome-wide significant Fst or iHS scores, including: *CYP3A4* (a potent oxidizer of steroids and drugs), *CYP3A5* (involved in oxidation of fatty acids and steroids in the liver), *CYP3A7* (the main *CYP* enzyme expressed in fetal livers) and *CYP3A43* (involved in testosterone metabolism). The *CYP* proteins play an important role in drug metabolism and in the synthesis of steroids from cholesterol [46].

## Discussion

In spite of a fat and cholesterol rich diet, the Maasai have low blood cholesterol levels and low incidence of heart disease and atherosclerosis. Cholesterol challenge studies in the 1970s [2] demonstrated that the Maasai are able to maintain cholesterol homeostasis in response to elevated levels of dietary cholesterol, and suggested that the mechanism of cholesterol homeostasis may have a genetic basis. In the present study, we used HapMap 3 data to investigate this possibility. Using 90–110 unrelated LWK individuals as a reference population, three complementary metrics (Fst, iHS and XP-EHH) were used to identify SNPs and chromosomal regions under selection in 143–156 unrelated MKK (Maasai) individuals in HapMap 3. The genomic regions and genes identified as selection candidates in MKK are shown in Tables 1,2,3 and Tables S1,S2,S3 for the Fst, iHS and XP-EHH metrics respectively. We identified seven genomic regions as strong candidates for selection using concordance between at least two of the metrics (Table 5). We now discuss some of the most interesting SNPs and regions identified for the role they may play in lactase persistence and lipid pathway selection in the Maasai.

Using Fst, the most significant non-synonymous SNP was the polymorphism rs2241883 located at 88.42 Mb on Chromosome 2 (Figure 2a, Table 1). This is a Threonine to Alanine substitution (T94A) in exon 3 of the *FABP1* (or *LFABP*) gene, a fatty acid binding protein expressed in liver. This locus was not detected by iHS or XP-EHH, suggesting either an increased local recombination rate or a more ancient selective sweep. The T94A polymorphism was strongly associated with lower levels of plasma triglycerides and LDL-cholesterol levels in a study of 826 individuals from Northern Germany [36]. A study of plasma concentrations of ApoB in 623 French Canadian men found that carriers of the A94 allele were protected against high ApoB levels when consuming a high fat and saturated fat diet, possibly because of diminished function of the protein *LFABP* due to a disruption in ligand binding [37]. *LFABP* knockout mice fed a high cholesterol, high saturated fat diet were protected against diet-induced obesity and lower levels of hepatic triglycerides compared to control mice, despite the absence of discernible differences in energy levels, food intake, or mal-absorption of fat induced obesity [47], [48]. The study concluded that “*LFABP* may function as a metabolic sensor in regulating lipid homeostasis” [47]. The protective C allele of this SNP is segregating in the Maasai at allele-frequency 0.44, suggesting that the effect of the T94A mutation on the *LFABP* pathway may be partly responsible for the homeostatic regulation of blood cholesterol in Maasai [1]–[3].

We found evidence for a strong recent selective sweep in a ∼1.7 Mb region on Chr2q21 (Fig 2, Table 1,2,3,4). This region is known to harbor polymorphisms conferring lactase persistence in Kenyans, and has been shown to be under strong recent selection. Tishkoff et al [28] performed a phenotype-genotype association study for lactase persistence on 470 Tanzanians, Kenyans and Sudanese who were genotyped at 123 SNPs, in a 3 Mb region surrounding the *LCT* and *MCM6* genes. The SNP known as G/C-14010 was found to have the most significant association with the lactase persistence phenotype in Kenyan Nilo-Saharan and Tanzanian Afro-Asiatic populations, as well as in a meta-analysis of all the populations combined. Tishkoff et al observed the C-14010 allele to occur at 32% frequency in Kenyan populations. As this SNP is in the upstream regulatory region of the gene *LCT*, the authors also studied the effect of this polymorphism on expression using luciferase assays in intestinal cells. They found that the C-14010 allele leads to a significantly higher expression. Furthermore, an iHS analysis of the haplotype background on which the SNP occurs indicated that the SNP is under selection in Kenyans and Tanzanians. We found that in the MKK samples from HapMap the C-14010 allele is segregating at high frequency (0.58). Thus, our results confirm the findings of Tishkoff et al, that C-14010 contributes towards selection for lactase persistence in the MKK samples from HapMap.

In addition to lactase persistence, the GWAS studies of [43] and [44] indicate that, in Europeans, the locus on Chr2q21 is associated with cholesterol levels. As this locus is also identified by our analysis, it may be associated with cholesterol levels in the Maasai. However, the allelic variants of the GWAS SNPs of [43], [44] that have high frequency in MKK are associated with an *increase* in cholesterol levels in Europeans. This might reflect the possibility that Europeans and Maasai have different sets of functional polymorphisms at this locus responsible for lower cholesterol levels: indeed it is known that the Maasai have an African polymorphism associated with lactase persistence, different from the one found in Europeans. It could also be that in the Maasai, the SNPs identified in our study are not themselves functional, but linked to functional variants that are not genotyped. Given the extended linkage disequilibrium (LD) in this region due to a selective sweep in both Europeans and the Maasai, this last possibility is especially important. The differing effects of the SNPs identified in the Maasai, as compared with the Europeans, could arise from the effects of differing modifier alleles at different loci in this region. These possibilities emphasize the difficulties associated with identifying true functional polymorphisms because of potential population specificity of SNP based studies. However, given the GWAS findings, and the strong signal of selection in MKK seen in our analysis, the *LCT* locus is a candidate region for identifying genotypic variants associated with cholesterol regulation in the Maasai.

We also identified a 261 kb locus on Chr 7 (the CYP3A locus) to be under selection using Fst and iHS (Tables 1, 2, 4). This locus has been identified in re-sequencing studies and genome-wide scans to be under positive selection in Africans and non-Africans [33], [49], [50] and is also under positive selection for salt sensitivity in equatorial populations [49], [51]. This locus contains the *CYP3A* (cytochrome P450, subfamily 3A) family of genes which are involved in cholesterol metabolism and steroid biosynthesis [46]. This family contains *CYP3A5*, a gene involved in fatty acid oxidation in liver, as well as *CYP3A7*, a gene encoding a CYP enzyme expressed in fetal livers. Variants in *CYP3A5* have been shown to reduce the efficacy of certain statins, drugs used to lower cholesterol biosynthesis [52]. Thus, the selection pressure at this locus, as identified by our analysis, coupled with its role in cholesterol metabolism, suggests that the CYP3A locus is an important candidate for cholesterol homeostasis in the Maasai.

Several other clusters identified to be under selection in MKK contain genes related to cholesterol metabolism, cholesterol biosynthesis and atherosclerosis. On Chr12q13, we identified a region spanning many genes with one of the highest Fst signals (Table 1). This locus contains the Apolipoprotein F (*APOF*) gene, involved in cholesterol transport and esterification [46], whose over-expression in mice reduces high density lipoprotein (HDL) cholesterol levels [53]. A cluster identified by iHS on chromosome 11q13.5 contains the gene Diacylglycerol O-acyltransferase 2 (*DGAT2*) (Table 3). This gene is involved in biosynthesis of triglycerols [54], [55] and has been implicated in hyperlipidemia [56] and fatty liver disease [57]. Another cluster on Chr7p21.1 identified by iHS, contains the Integrin Beta 8 (*ITGB8)* gene (Table 3) implicated as a quantitative trait locus (QTL) for fibrinogen plasma levels in a study involving 3600 Native Americans [58]. Fibrinogen levels are associated with risks for several cardiovascular diseases [59], and play a role in the pathogenesis of atherosclerosis [58]. XP-EHH identified a genome-wide significant region on chromosome 16q22.2–22.3, containing the gene Craniofacial Development Protein 1 (*CFDP1*) (Table 4). A GWAS showed that this region is associated with low levels of HDL cholesterol in ∼400 French-Canadians [60].

Our results identified several genes and loci involved in cholesterol metabolism as selection candidates in the Maasai. Thus, our findings suggest that the Maasai are adapted for a high-cholesterol and high-fat diet. The traditional diet of the Maasai is rich in saturated fats and cholesterol, and low in carbohydrates. Similar ketogenic diets are often used to treat epileptic seizures in children [61], [62]. Early complications of these diets include hypertriglyceridemia, hypercholesterolemia, and low levels of HDL, and late complications include osteopenia, renal stones, and cardiomyopathy [62], [63]. This suggests that a diet rich in fat and cholesterol from childhood can exert a strong diet-induced selection pressure on survival and reproductive success.

Maasai social customs may also favor genetic selection against diseases of the elderly. Maasai are both polygynous and gerontocratic, and older men routinely marry nulliparous young women [64]–[70]. Maasai women are also permitted, at their discretion, to have sex with members of their husbands’ age set, a form of open marriage that provides older men with opportunities to reproduce [15], [64]–[67], [69]. Finally, extramarital sex between older men and the wives of younger men sometimes occurs [64], [68]. Such mating practices may facilitate the spread of protective adaptations for old-age diseases.

## Summary

Field studies showed that, in spite of a high fat and high cholesterol diet, the Maasai have low levels of cardiac disease and atherosclerosis. In this paper, we present results from a genome wide scan of the HapMap 3 SNP data using the Fst, iHS and XP-EHH statistics to identify genomic regions under selection in the Maasai. We identify regions containing genes involved in lactose and lipid metabolism which are under selection in the Maasai. Our analysis suggest that the identified regions harbor known and novel genetic polymorphisms responsible for the unusual lipid metabolism, cholesterol homeostasis, protection against cardiac diseases and adult lactase persistence in the Maasai.

## Methods

### 

#### Ethics statement

The data analyzed was public SNP data from the HapMap Consortium http://hapmap.ncbi.nlm.nih.gov/. No consent was required.

#### Data used

HapMap 3 release 3 SNP genotype data for founders from the Maasai in Kinyawa, Kenya (MKK) (n = 156), the Luhya in Webuye, Kenya (LWK) (n = 110), African-Americans in Southwest USA (ASW) (n = 53), the Yoruba in Ibadan, Nigeria (YRI) (n = 147), and Utah residents of Northern and Western European ancestry (CEU) (n = 112) was downloaded from http://snp.cshl.org/. Using PLINK (http://pngu.mgh.harvard.edu/~purcell/plink/), we filtered to retain only SNPs common to all populations.

Hapmap 3 release 2 autosomal haplotype data for the MKK (n = 143) and LWK (n = 90) was downloaded from http://hapmap.ncbi.nlm.nih.gov/downloads/phasing/2009-02_phaseIII/HapMap3_r2/. The data was phased using IMPUTE++ [71]. SNPs were pre-filtered for Hardy Weinberg equilibrium and for low frequency of Mendel errors (http://hapmap.ncbi.nlm.nih.gov/downloads/genotypes/2010-05_phaseIII/00README.txt). Genetic maps were downloaded from http://hapmap.ncbi.nlm.nih.gov/downloads/recombination/2008-03_rel22_B36/rates/to obtain the genetic map position of the SNPs in cM.

#### STRUCTURE computation

Using PLINK, genotype data for MKK, LWK, YRI, ASW and CEU was filtered to exclude SNPs with minor allele frequency <1% or SNPs where more than 1% of the genotype data was missing. Restricting the samples to founders resulted in 1,325,342 common SNPs for 578 individuals. We further restricted the genotype data to a random subset of ∼1% of these SNPs (12,999 SNPs) and ran the “no admixture” model in STRUCTURE [34] version 2.3. We found that k = 6 ancestral populations fit the data best. Further details are in Appendix S1.

#### Fst computation

Using PLINK, we retained 1,175,055 autosomal SNPs in Hardy Weinberg equilibrium (p>0.05) and with minor allele frequency >5% in either population (LWK and MKK). We then computed Fst using the method of [30]. Two tests were used to assess statistical significance, a Bonferroni corrected permutation test (p-value p_B_), and an empirical p-value that compared the Fst of a SNP to the Fst distribution of intergenic SNPs. Gene positions were from the human genome build 37 (GRCh37/hg19) available at http://hgdownload.cse.ucsc.edu/goldenPath/hg19/database/knownGene.txt.gz. To avoid linkage with genes and promoter regions, we define intergenic regions as those that are at least 50 kb away from the start or stop site of a gene. For the remaining genic or near-gene SNPs, we calculated an empirical p-value (p_E_) given by the fraction of intergenic SNPs with greater Fst. This procedure identified 1,232 SNPs with p_B_ <8.6E−6 and p_E_ <0.001 that are the top candidates for selection using Fst (Table S1). These SNPs were then clustered into regions of high linkage (Table 1, Table S2) using the method described below (details of the Fst calculation are in Appendix S2).

#### iHS computation

Autosomal haplotype data for 991,737 SNPs in MKK with minor allele frequency >10% were used to calculate raw iHS scores as in Voight et al [33]. These raw iHS scores were binned on the basis of derived allele-frequency, and the scores in each bin were standard normalized to zero mean and unit variance. Genomic sliding windows of 50 SNPs were ranked by the percentage of SNPs with |iHS|>2. The SNPs with |iHS|>2 that occured in the top 0.02% of non-overlapping windows were selected as top candidates for selection by iHS (Table S3). These were then clustered into regions of high linkage (Table 3, Table S4) using the method described below (details of the iHS calculation are in Appendix S3).

#### XP-EHH computation

Autosomal haplotype data for 1,373,755 SNPs in MKK and LWK was mapped to genomic locations in the human genome, build 37 (GRCh37). XP-EHH scores were calculated using the code at http://hgdp.uchicago.edu/Software/xpehh.tar. The XP-EHH scores were fit to a normal distribution, which identified the threshold for genome-wide significance to be XP-EHH ≥4.796 (Bonferroni corrected p<0.05, two-tailed test). The SNP that exceeded this threshold were chosen as top candidates for selection by XP-EHH (Table S5). These SNPs were clustered into regions of high linkage (Table 4, Table S6) using the method described below (details of the XP-EHH calculation are in Appendix S4).

#### LD clustering of SNPs

The SNPs identified as candidates for selection by each of the above methods were clustered using genotype R^2^ as an estimator of linkage disequilibrium. We used the criteria that for a SNP to be included in a cluster, it must have genotype R^2^≥0.25 with at least one other SNP in the cluster (the justification for this choice of cutoff is given in Appendix S2).

More concretely, for the SNPs identified by the methods above, we used PLINK to extract a file of raw genotype data from the HapMap genotype data file for MKK. These files contained a matrix of genotype values, whose columns were labeled by SNPs and rows labeled by individuals. We imported this genotype matrix into the statistical package R, to calculate a SNP x SNP Pearson correlation matrix. This correlation matrix was then used to construct a SNP x SNP adjacency matrix whose entries are 1 if R^2^≥0.25 and 0 if R^2^<0.25. The problem of finding linked clusters of SNPs then translates to identifying the connected components of the graph described by this adjacency matrix. This computation was performed in Python using the NetworkX package (http://networkx.lanl.gov/).

#### Sequencing loci in LCT/MCM6 and RAB3GAP1

Forward and reverse primers for Sanger sequencing were chosen using Primer3 (http://frodo.wi.mit.edu/primer3/), and checked for absence of homologies to other parts of the human genome using BLAT. The details of the primers, the loci sequenced and the samples used are in Appendix S5.

## Supporting Information

Table S1 1,232 genic or near-gene SNPs identified by Fst as top candidates for selection (p_B_<8.6E−6 and p_E_ <0.001). Significance was assessed using an exact permutation test (Bonferroni corrected p-value p_B_ shown in column Q) and an empirical test based on the Fst distribution of intergenic SNPs (p_E_ : column R). Columns H-M list the number of individuals with each genotype (A1 homozygous, heterozygous, A2 homozygous) in MKK and LWK.
(XLS)

Table S2 Genomic regions identified as selection candidates in MKK using Fst and clustering. SNPs having empirical p-value <0.001 with respect to the distribution of intergenic Fst scores were clustered into regions of high linkage disequilibrium using genotype R^2^ between SNPs. Clusters with maximum XP-EHH score >3 were identified as being MKK associated. Also listed are the maximum Fst score and the maximum XP-EHH score of any SNP in the genomic extent of the cluster.
(XLSX)

Table S3 SNPs identified as selection candidates using the iHS metric. Sliding windows of 50 SNPs each were scored for fraction of SNPs with |iHS| >2. SNPs with |iHS| >2 that occur in the top 0.02% of non-overlapping genomic windows are listed.
(XLS)

Table S4 Genomic regions identified as selection candidates in MKK using the iHS statistic. Sliding windows of 50 SNPs each were scored for the fraction of SNPs with |iHS| >2. The top 0.02% of non-overlapping windows were identified as candidates for selection. These windows were then merged on the basis of linkage disequilibrium (estimated using genotype R^2^ between SNPs with |iHS| >2).
(XLS)

Table S5 SNPs identified as candidates for selection in MKK using the XP-EHH statistic, with LWK as the reference population. All SNPs listed have scores exceeding the threshold for genome-wide significance (XP-EHH > = 4.796, two-tailed bonferroni corrected p< = 0.05).
(XLSX)

Table S6 Genomic regions identified as selection candidates in MKK using the XP-EHH statistic, with LWK as the reference population. SNPs with genome-wide significant scores (XP-EHH > = 4.796, two-tailed Bonferroni corrected p< = 0.05) were assigned to a cluster if they had genotype R^2^≥0.25 with another SNP in the cluster. This identified contiguous genomic regions as candidates for selective sweeps. Clusters that overlapped in genomic extent were merged. Column F and G list the number of significant SNPs occurring in the genomic extent of each cluster, and their rsids.
(XLSX)

Table S7 Common regions and SNPs identified to be under selection in MKK by our analysis (using Fst, iHS and XP-EHH) and by the International HapMap Consortium (using the CMS test). Only those SNPs identified by the HapMap Consortium which were also identified by our analysis (i.e. passed genome-wide significance thresholds for the Fst, iHS, and XP-EHH statistic respectively) are listed.
(XLSX)

Appendix S1 Details of STRUCTURE calculation.
(DOC)

Appendix S2 Details of Fst calculation, p-values and SNP clustering for Fst and XP-EHH.
(DOC)

Appendix S3 Details of iHS calculation.
(DOC)

Appendix S4 Details of XP-EHH calculation.
(PDF)

Appendix S5 Details of Sequencing in LCT/MCM6 locus
(DOC)

Appendix S6 Plots of Fst, XP-EHH and iHS for all chromosomes.
(PDF)

## References

[1] Biss K, Ho KJ, Mikkelson B, Lewis L, Taylor CB (1971) Some unique biologic characteristics of the Masai of East Africa. The New England journal of medicine 284: 694–699. Available: http://www.ncbi.nlm.nih.gov/pubmed/5107799. Accessed 2011 Oct 3. 10.1056/NEJM197104012841304 5107799

[2] Ho KJ, Biss K, Mikkelson B, Lewis LA, Taylor CB (1971) The Masai of East Africa: some unique biological characteristics. Archives of pathology 91: 387–410. Available: http://www.ncbi.nlm.nih.gov/pubmed/4103135. Accessed 2011 Oct 3. 4103135

[3] Taylor CB, Ho KJ (1971) Studies on the Masai. The American journal of clinical nutrition 24: 1291–1293. Available: http://www.ncbi.nlm.nih.gov/pubmed/5116471. Accessed 2011 Oct 4. 10.1093/ajcn/24.11.1291 5116471

[4] Århem K (1989) The Cultural Connotations of Milk, Meat, and Blood in the Pastoral Maasai Diet. Anthropos 84: 1–23.

[5] Kromhout D (1995) Dietary Saturated and transFatty Acids and Cholesterol and 25-Year Mortality from Coronary Heart Disease: The Seven Countries Study. Preventive Medicine 24: 308–315. Available: http://dx.doi.org/10.1006/pmed.1995.1049. Accessed 2012 Jul 21. 10.1006/pmed.1995.1049 7644455

[6] Ho KJ, Mikkelson B, Lewis LA, Feldman SA, Taylor CB (1972) Alaskan Arctic Eskimo: responses to a customary high fat diet. The American journal of clinical nutrition 25: 737–745. Available: http://www.ncbi.nlm.nih.gov/pubmed/5046723. Accessed 2012 Jul 21. 10.1093/ajcn/25.8.737 5046723

[7] Bang HO, Dyerberg J, Sinclair HM (1980) The composition of the Eskimo food in north western Greenland. The American journal of clinical nutrition 33: 2657–2661. Available: http://www.ncbi.nlm.nih.gov/pubmed/7435433. Accessed 2012 Jul 21. 10.1093/ajcn/33.12.2657 7435433

[8] Bang HO, Dyerberg J, Nielsen AB (1971) Plasma lipid and lipoprotein pattern in Greenlandic West-coast Eskimos. Lancet 1: 1143–1145. Available: http://www.ncbi.nlm.nih.gov/pubmed/4102857. Accessed 2012 Jul 21. 10.1016/s0140-6736(71)91658-8 4102857

[9] Brotons C, Ribera A, Perich RM, Abrodos D, Magaña P, et al. (1998) Worldwide distribution of blood lipids and lipoproteins in childhood and adolescence: a review study. Atherosclerosis 139: 1–9. Available: http://www.ncbi.nlm.nih.gov/pubmed/9699886. Accessed 2012 Jul 21. 10.1016/s0021-9150(98)00056-2 9699886

[10] Mann GV, Shaffer RD, Anderson RS, Sandstead HH (n.d.) Cardiovascular disease in the Masai. Journal of atherosclerosis research 4: 289–312. Available: http://www.ncbi.nlm.nih.gov/pubmed/14193818. Accessed 2011 Oct 3. 10.1016/s0368-1319(64)80041-7 14193818

[11] Mann GV, Shaffer RD, Rich A (1965) Physical fitness and immunity to heart-disease in Masai. Lancet 2: 1308–1310. Available: http://www.ncbi.nlm.nih.gov/pubmed/4165302. Accessed 2011 Oct 3. 10.1016/s0140-6736(65)92337-8 4165302

[12] Gibney MJ, Burstyn PG (1980) Milk, serum cholesterol, and the Maasai. A hypothesis. Atherosclerosis 35: 339–343. Available: http://www.ncbi.nlm.nih.gov/pubmed/6987994. Accessed 2011 Oct 3. 10.1016/0021-9150(80)90131-8 6987994

[13] Johns T, Mahunnah RL, Sanaya P, Chapman L, Ticktin T (1999) Saponins and phenolic content in plant dietary additives of a traditional subsistence community, the Batemi of Ngorongoro District, Tanzania. Journal of ethnopharmacology 66: 1–10. Available: http://www.ncbi.nlm.nih.gov/pubmed/10432201. Accessed 2011 Oct 3. 10.1016/s0378-8741(98)00179-2 10432201

[14] Coast E (2001) Maasai demography University of London, University College London. Available: http://eprints.lse.ac.uk/264/1/Maasai_Demography_PhD.pdf. Accessed 2011 Oct 3.

[15] Hollis AC (1910) A Note on the Masai System of Relationship and Other Matters Connected Therewith. The Journal of the Royal Anthropological Institute of Great Britain and Ireland 40: 473–482. Available: http://www.jstor.org/pss/2843267. Accessed 2011 Oct 3.

[16] Mattson FH, Erickson BA, Kligman AM (1972) Effect of dietary cholesterol on serum cholesterol in man. The American journal of clinical nutrition 25: 589–594. Available: http://www.ncbi.nlm.nih.gov/pubmed/5064235. Accessed 2012 Jul 21. 10.1093/ajcn/25.6.589 5064235

[17] Rader DJ, Cohen J, Hobbs HH (2003) Monogenic hypercholesterolemia: new insights in pathogenesis and treatment. The Journal of clinical investigation 111: 1795–1803. Available: http://www.pubmedcentral.nih.gov/articlerender.fcgi?artid=161432&tool=pmcentrez&rendertype=abstract. Accessed 2011 Oct 3. 10.1172/JCI18925 PMC161432 12813012

[18] Lusis AJ (2000) Atherosclerosis. Nature 407: 233–241. Available: http://www.pubmedcentral.nih.gov/articlerender.fcgi?artid=2826222&tool=pmcentrez&rendertype=abstract. Accessed 2011 Aug 26. 10.1038/35025203 PMC2826222 11001066

[19] Lusis AJ, Fogelman AM, Fonarow GC (2004) Genetic basis of atherosclerosis: part I: new genes and pathways. Circulation 110: 1868–1873. Available: http://www.ncbi.nlm.nih.gov/pubmed/15451808. Accessed 2011 Jun 23. 10.1161/01.CIR.0000143041.58692.CC 15451808

[20] Lusis AJ, Fogelman AM, Fonarow GC (2004) Genetic basis of atherosclerosis: part II: clinical implications. Circulation 110: 2066–2071. Available: http://www.ncbi.nlm.nih.gov/pubmed/15466657. Accessed 2011 Jul 15. 10.1161/01.CIR.0000143098.98869.F8 15466657

[21] Wang WYS, Barratt BJ, Clayton DG, Todd JA (2005) Genome-wide association studies: theoretical and practical concerns. Nature reviews Genetics 6: 109–118. Available: http://www.ncbi.nlm.nih.gov/pubmed/15716907. Accessed 2011 Jul 4. 10.1038/nrg1522 15716907

[22] Hardy J, Singleton A (2009) Genomewide association studies and human disease. The New England journal of medicine 360: 1759–1768. Available: http://www.ncbi.nlm.nih.gov/pubmed/19369657. Accessed 2011 Oct 3. 10.1056/NEJMra0808700 PMC3422859 19369657

[23] Ku CS, Loy EY, Pawitan Y, Chia KS (2010) The pursuit of genome-wide association studies: where are we now? Journal of human genetics 55: 195–206. Available: http://dx.doi.org/10.1038/jhg.2010.19. Accessed 2011 Jun 14. 10.1038/jhg.2010.19 20300123

[24] Johnson AD, O’Donnell CJ (2009) An open access database of genome-wide association results. BMC medical genetics 10: 6. Available: http://www.pubmedcentral.nih.gov/articlerender.fcgi?artid=2639349&tool=pmcentrez&rendertype=abstract. Accessed 2011 Jul 16. 10.1186/1471-2350-10-6 PMC2639349 19161620

[25] Manolio TA (2010) Genomewide association studies and assessment of the risk of disease. The New England journal of medicine 363: 166–176. Available: http://www.ncbi.nlm.nih.gov/pubmed/20647212. Accessed 2011 Jul 30. 10.1056/NEJMra0905980 20647212

[26] Itan Y, Powell A, Beaumont MA, Burger J, Thomas MG (2009) The origins of lactase persistence in Europe. PLoS computational biology 5: e1000491. Available: http://www.pubmedcentral.nih.gov/articlerender.fcgi?artid=2722739&tool=pmcentrez&rendertype=abstract. Accessed 2011 July 15. 10.1371/journal.pcbi.1000491 PMC2722739 19714206

[27] Hanotte O, Bradley DG, Ochieng JW, Verjee Y, Hill EW, et al. (2002) African pastoralism: genetic imprints of origins and migrations. Science (New York, NY) 296: 336–339. Available: http://www.ncbi.nlm.nih.gov/pubmed/11951043. Accessed 2011 Jul 28. 10.1126/science.1069878 11951043

[28] Tishkoff SA, Reed FA, Ranciaro A, Voight BF, Babbitt CC, et al. (2007) Convergent adaptation of human lactase persistence in Africa and Europe. Nature genetics 39: 31–40. Available: http://dx.doi.org/10.1038/ng1946. Accessed 2011 Jul 7. 10.1038/ng1946 PMC2672153 17159977

[29] Altshuler DM, Gibbs RA, Peltonen L, Dermitzakis E, Schaffner SF, et al. (2010) Integrating common and rare genetic variation in diverse human populations. Nature 467: 52–58. Available: http://www.pubmedcentral.nih.gov/articlerender.fcgi?artid=3173859&tool=pmcentrez&rendertype=abstract. Accessed 2011 Jul 18. 10.1038/nature09298 PMC3173859 20811451

[30] Reynolds J, Weir BS, Cockerham CC (1983) Estimation of the coancestry coefficient: basis for a short-term genetic distance. Genetics 105: 767–779. Available: http://www.pubmedcentral.nih.gov/articlerender.fcgi?artid=1202185&tool=pmcentrez&rendertype=abstract. Accessed 2011 Oct 3. 10.1093/genetics/105.3.767 PMC1202185 17246175

[31] Sabeti PC, Varilly P, Fry B, Lohmueller J, Hostetter E, et al. (2007) Genome-wide detection and characterization of positive selection in human populations. Nature 449: 913–918. Available: http://www.pubmedcentral.nih.gov/articlerender.fcgi?artid=2687721&tool=pmcentrez&rendertype=abstract. Accessed 2011 Jul 4. 10.1038/nature06250 PMC2687721 17943131

[32] Sabeti PC, Reich DE, Higgins JM, Levine HZP, Richter DJ, et al. (2002) Detecting recent positive selection in the human genome from haplotype structure. Nature 419: 832–837. Available: http://dx.doi.org/10.1038/nature01140. Accessed 2011 Jul 22. 10.1038/nature01140 12397357

[33] Voight BF, Kudaravalli S, Wen X, Pritchard JK (2006) A map of recent positive selection in the human genome. PLoS biology 4: e72. Available: http://dx.plos.org/10.1371/journal.pbio.0040072. Accessed 2011 Jun 10. 10.1371/journal.pbio.0040072 PMC1382018 16494531

[34] Hubisz MJ, Falush D, Stephens M, Pritchard JK (2009) Inferring weak population structure with the assistance of sample group information. Molecular Ecology Resources 9: 1322–1332 Available: http://blackwell-synergy.com/doi/abs/10.1111/j.1755-0998.2009.02591.x. 21564903 10.1111/j.1755-0998.2009.02591.x PMC3518025

[35] Tishkoff SA, Reed FA, Friedlaender FR, Ehret C, Ranciaro A, et al. (2009) The genetic structure and history of Africans and African Americans. Science (New York, NY) 324: 1035–1044. Available: http://www.sciencemag.org/content/324/5930/1035.abstract. Accessed 2011 Jun 17. 10.1126/science.1172257 PMC2947357 19407144

[36] Fisher E, Weikert C, Klapper M, Lindner I, Möhlig M, et al. (2007) L-FABP T94A is associated with fasting triglycerides and LDL-cholesterol in women. Molecular genetics and metabolism 91: 278–284. Available: http://www.ncbi.nlm.nih.gov/pubmed/17485234. Accessed 2011 Oct 3. 10.1016/j.ymgme.2007.03.002 17485234

[37] Robitaille J, Brouillette C, Lemieux S, Pérusse L, Gaudet D, et al. (2004) Plasma concentrations of apolipoprotein B are modulated by a gene–diet interaction effect between the LFABP T94A polymorphism and dietary fat intake in French-Canadian men. Molecular genetics and metabolism 82: 296–303. Available: http://www.ncbi.nlm.nih.gov/pubmed/15308127. Accessed 2011 Oct 3. 10.1016/j.ymgme.2004.06.002 15308127

[38] Enattah NS, Sahi T, Savilahti E, Terwilliger JD, Peltonen L, et al. (2002) Identification of a variant associated with adult-type hypolactasia. Nature genetics 30: 233–237. Available: http://dx.doi.org/10.1038/ng826. Accessed 2011 Aug 8. 10.1038/ng826 11788828

[39] Ingram CJE, Elamin MF, Mulcare CA, Weale ME, Tarekegn A, et al. (2007) A novel polymorphism associated with lactose tolerance in Africa: multiple causes for lactase persistence? Human genetics 120: 779–788. Available: http://www.ncbi.nlm.nih.gov/pubmed/17120047. Accessed 2012 Jul 21. 10.1007/s00439-006-0291-1 17120047

[40] Enattah NS, Jensen TGK, Nielsen M, Lewinski R, Kuokkanen M, et al. (2008) Independent introduction of two lactase-persistence alleles into human populations reflects different history of adaptation to milk culture. American journal of human genetics 82: 57–72. Available: http://www.pubmedcentral.nih.gov/articlerender.fcgi?artid=2253962&tool=pmcentrez&rendertype=abstract. Accessed 2012 Jul 21. 10.1016/j.ajhg.2007.09.012 PMC2253962 18179885

[41] Bersaglieri T, Sabeti PC, Patterson N, Vanderploeg T, Schaffner SF, et al. (2004) Genetic signatures of strong recent positive selection at the lactase gene. American journal of human genetics 74: 1111–1120. Available: http://www.pubmedcentral.nih.gov/articlerender.fcgi?artid=1182075&tool=pmcentrez&rendertype=abstract. Accessed 2011 Oct 3. 10.1086/421051 PMC1182075 15114531

[42] Coelho M, Luiselli D, Bertorelle G, Lopes AI, Seixas S, et al. (2005) Microsatellite variation and evolution of human lactase persistence. Human genetics 117: 329–339. Available: http://www.ncbi.nlm.nih.gov/pubmed/15928901. Accessed 2011 Sep 7. 10.1007/s00439-005-1322-z 15928901

[43] Teslovich TM, Musunuru K, Smith AV, Edmondson AC, Stylianou IM, et al. (2010) Biological, clinical and population relevance of 95 loci for blood lipids. Nature 466: 707–713. Available: http://www.pubmedcentral.nih.gov/articlerender.fcgi?artid=3039276&tool=pmcentrez&rendertype=abstract. Accessed 2011 Jun 14. 10.1038/nature09270 PMC3039276 20686565

[44] Silander K, Alanne M, Kristiansson K, Saarela O, Ripatti S, et al. (2008) Gender differences in genetic risk profiles for cardiovascular disease. PloS one 3: e3615. Available: http://www.pubmedcentral.nih.gov/articlerender.fcgi?artid=2574036&tool=pmcentrez&rendertype=abstract. Accessed 2011 Oct 3. 10.1371/journal.pone.0003615 PMC2574036 18974842

[45] Ma L, Yang J, Runesha HB, Tanaka T, Ferrucci L, et al. (2010) Genome-wide association analysis of total cholesterol and high-density lipoprotein cholesterol levels using the Framingham heart study data. BMC medical genetics 11: 55. Available: http://www.mendeley.com/research/genomewide-association-analysis-of-total-cholesterol-and-highdensity-lipoprotein-cholesterol-levels-using-the-framingham-heart-study-data-3/. Accessed 2011 Jul 29. 10.1186/1471-2350-11-55 PMC2867786 20370913

[46] Pruitt KD, Tatusova T, Maglott DR (2005) NCBI Reference Sequence (RefSeq): a curated non-redundant sequence database of genomes, transcripts and proteins. Nucleic acids research 33: D501–D504. Available: http://www.pubmedcentral.nih.gov/articlerender.fcgi?artid=539979&tool=pmcentrez&rendertype=abstract. Accessed 2011 Jul 30. 10.1093/nar/gki025 PMC539979 15608248

[47] Newberry EP, Xie Y, Kennedy SM, Luo J, Davidson NO (2006) Protection against Western diet-induced obesity and hepatic steatosis in liver fatty acid-binding protein knockout mice. Hepatology (Baltimore, Md) 44: 1191–1205. Available: http://www.ncbi.nlm.nih.gov/pubmed/17058218. Accessed 2011 Oct 3. 10.1002/hep.21369 17058218

[48] Newberry EP, Kennedy SM, Xie Y, Luo J, Davidson NO (2009) Diet-induced alterations in intestinal and extrahepatic lipid metabolism in liver fatty acid binding protein knockout mice. Molecular and cellular biochemistry 326: 79–86. Available: http://www.pubmedcentral.nih.gov/articlerender.fcgi?artid=3004673&tool=pmcentrez&rendertype=abstract. Accessed 2011 Oct 3. 10.1007/s11010-008-0002-4 PMC3004673 19116776

[49] Thompson EE, Kuttab-Boulos H, Witonsky D, Yang L, Roe BA, et al. (2004) CYP3A variation and the evolution of salt-sensitivity variants. American journal of human genetics 75: 1059–1069. Available: http://www.pubmedcentral.nih.gov/articlerender.fcgi?artid=1182141&tool=pmcentrez&rendertype=abstract. Accessed 2011 Jul 30. 10.1086/426406 PMC1182141 15492926

[50] Chen X, Wang H, Zhou G, Zhang X, Dong X, et al. (2009) Molecular population genetics of human CYP3A locus: signatures of positive selection and implications for evolutionary environmental medicine. Environmental health perspectives 117: 1541–1548. Available:http://www.pubmedcentral.nih.gov/articlerender.fcgi?artid=2790508&tool=pmcentrez&rendertype=abstract. Accessed 2011 Aug 10. 10.1289/ehp.0800528 PMC2790508 20019904

[51] Kuehl P, Zhang J, Lin Y, Lamba J, Assem M, et al. (2001) Sequence diversity in CYP3A promoters and characterization of the genetic basis of polymorphic CYP3A5 expression. Nature genetics 27: 383–391. Available: http://www.ncbi.nlm.nih.gov/pubmed/11279519. Accessed 2011 Aug 13. 10.1038/86882 11279519

[52] Kivistö KT, Niemi M, Schaeffeler E, Pitkälä K, Tilvis R, et al. (2004) Lipid-lowering response to statins is affected by CYP3A5 polymorphism. Pharmacogenetics 14: 523–525. Available: http://www.ncbi.nlm.nih.gov/pubmed/15284534. Accessed 2011 Oct 3. 10.1097/01.fpc.0000114762.78957.a5 15284534

[53] Lagor WR, Brown RJ, Toh S-A, Millar JS, Fuki IV, et al. (2009) Overexpression of apolipoprotein F reduces HDL cholesterol levels in vivo. Arteriosclerosis, thrombosis, and vascular biology 29: 40–46. Available: http://www.pubmedcentral.nih.gov/articlerender.fcgi?artid=2766561&tool=pmcentrez&rendertype=abstract. Accessed 2011 Aug 25. 10.1161/ATVBAHA.108.177105 PMC2766561 19008531

[54] Lardizabal KD, Mai JT, Wagner NW, Wyrick A, Voelker T, et al. (2001) DGAT2 is a new diacylglycerol acyltransferase gene family: purification, cloning, and expression in insect cells of two polypeptides from Mortierella ramanniana with diacylglycerol acyltransferase activity. The Journal of biological chemistry 276: 38862–38869. Available: http://www.ncbi.nlm.nih.gov/pubmed/11481333. Accessed 2011 Aug 1. 10.1074/jbc.M106168200 11481333

[55] Cases S, Stone SJ, Zhou P, Yen E, Tow B, et al. (2001) Cloning of DGAT2, a second mammalian diacylglycerol acyltransferase, and related family members. The Journal of biological chemistry 276: 38870–38876. Available: http://www.ncbi.nlm.nih.gov/pubmed/11481335. Accessed 2011 Aug 2. 10.1074/jbc.M106219200 11481335

[56] Yu XX, Murray SF, Pandey SK, Booten SL, Bao D, et al. (2005) Antisense oligonucleotide reduction of DGAT2 expression improves hepatic steatosis and hyperlipidemia in obese mice. Hepatology (Baltimore, Md) 42: 362–371. Available: http://www.ncbi.nlm.nih.gov/pubmed/16001399. Accessed 2011 Oct 3. 10.1002/hep.20783 16001399

[57] Kantartzis K, Machicao F, Machann J, Schick F, Fritsche A, et al. (2009) The DGAT2 gene is a candidate for the dissociation between fatty liver and insulin resistance in humans. Clinical science (London, England: 1979) 116: 531–537. Available: http://www.ncbi.nlm.nih.gov/pubmed/18980578. Accessed 2011 Oct 3. 10.1042/CS20080306 18980578

[58] Best LG, North KE, Li X, Palmieri V, Umans JG, et al. (2008) Linkage study of fibrinogen levels: the Strong Heart Family Study. BMC medical genetics 9: 77. Available: http://www.pubmedcentral.nih.gov/articlerender.fcgi?artid=2518547&tool=pmcentrez&rendertype=abstract. Accessed 2011 Oct 3. 10.1186/1471-2350-9-77 PMC2518547 18700015

[59] Danesh J, Lewington S, Thompson SG, Lowe GDO, Collins R, et al. (2005) Plasma fibrinogen level and the risk of major cardiovascular diseases and nonvascular mortality: an individual participant meta-analysis. JAMA: the journal of the American Medical Association 294: 1799–1809. Available: http://www.ncbi.nlm.nih.gov/pubmed/16219884. Accessed 2011 Aug 9. 10.1001/jama.294.14.1799 16219884

[60] Dastani Z, Pajukanta P, Marcil M, Rudzicz N, Ruel I, et al.. (2010) Fine mapping and association studies of a high-density lipoprotein cholesterol linkage region on chromosome 16 in French-Canadian subjects. European journal of human genetics: EJHG 18: 342–347. Available:/pmc/articles/PMC2824775/?report = abstract. Accessed 2012 Mar 22. 10.1038/ejhg.2009.157 PMC2824775 19844255

[61] Neal EG, Chaffe H, Schwartz RH, Lawson MS, Edwards N, et al. (2008) The ketogenic diet for the treatment of childhood epilepsy: a randomised controlled trial. Lancet neurology 7: 500–506. Available: http://www.ncbi.nlm.nih.gov/pubmed/18456557. Accessed 2011 Sep 14. 10.1016/S1474-4422(08)70092-9 18456557

[62] Shorvon SD, Perucca E, Fish D, Dodson WE, editors (2004) The Treatment of Epilepsy. 2nd ed. Wiley-Blackwell. p. Available: http://doi.wiley.com/10.1002/9780470752463. Accessed 2011 Oct 3.

[63] Kang HC, Chung DE, Kim DW, Kim HD (2004) Early- and late-onset complications of the ketogenic diet for intractable epilepsy. Epilepsia 45: 1116–1123. Available: http://www.ncbi.nlm.nih.gov/pubmed/15329077. Accessed 2011 Oct 3. 10.1111/j.0013-9580.2004.10004.x 15329077

[64] Spencer P (1988) The Maasai of Matapato: A Study of Rituals of Rebellion (International African Library). Indiana University Press. p. Available: http://www.amazon.com/Maasai-Matapato-Rituals-Rebellion-International/dp/0253336252. Accessed 2011 Oct 4.

[65] Spencer P (2003) Time, Space and the Unknown: Maasai Configurations of Power and Providence. Routledge. p. Available: http://www.amazon.com/Time-Space-Maasai-Configurations-Providence/dp/041531724X. Accessed 2011 Oct 4.

[66] Sankan SSO (1973) The Maasai. East African Literature Bureau, Nairobi. p. Available: http://www.amazon.com/Maasai-S-S-Ole-Sankan/dp/B00158DG6G. Accessed 2011 Oct 3.

[67] Mitzlaff U von (1994) Maasai women: Life in a patriarchal society: field research among the Parakuyo, Tanzania. Tanzania Publishing House. p. Available: http://www.amazon.com/Maasai-women-patriarchal-research-Parakuyo/dp/B0006F65DK. Accessed 2011 Oct 3.

[68] Jacobs AH (1965) The traditional political organization of the pastoral Masai Oxford University. Available: http://www.opengrey.eu/item/display/10068/528207. Accessed 2011 Oct 4.

[69] Jacobs AH (1973) The pastoral Masai of Kenya and Tanzania, in Cultural Source Materials for Population Planning in East Africa Vol 3. Molnos A, editor Nairobi: East African Publishing House. p. Available: http://catalogue.nla.gov.au/Record/758095.

[70] Cronk L (2004) From Mukogodo To Maasai: Ethnicity And Cultural Change In Kenya (Westview Case Studies in Anthropology). Boulder, CO: Westview Press. p. Available: http://www.amazon.com/Mukogodo-Maasai-Ethnicity-Cultural-Anthropology/dp/0813340942. Accessed 2011 Oct 3.

[71] Howie BN, Donnelly P, Marchini J (2009) A flexible and accurate genotype imputation method for the next generation of genome-wide association studies. PLoS genetics 5: e1000529. Available: http://dx.plos.org/10.1371/journal.pgen.1000529. Accessed 2012 Mar 14. 10.1371/journal.pgen.1000529 PMC2689936 19543373

