# Supplementary material for: Lactase Persistence and Lipid Pathway Selection in the Maasai
Source: PLoS One. 2012 Sep 28;7(9):e44751. doi: 10.1371/journal.pone.0044751 (PMC3461017; doi:10.1371/journal.pone.0044751)
Supplement: Appendix S1 — Details of STRUCTURE calculation. (DOC) [file pone.0044751.s008.doc]

**Appendix S1: Details of *Structure* calculation**

Using PLINK [R.A1.1], genotype data from HapMap 3 release 3 [R.A1.2] for the four populations with African ancestry (MKK, LWK, YRI, ASW) and Central Europeans (CEU) was processed to exclude SNPs with minor allele frequency < 0.01 or SNPs where more than 1% of the genotype data was missing. Restricting the samples to founders resulted in 1,325,342 SNPs on 578 individuals. This data was further reduced by retaining only genotype data for a random subset of ~1% of these SNPs using *thin* option in PLINK, leaving a dataset for 578 samples for 12,999 SNPs.

On this data, we used *Structure* [R.A1.3] version 2.3 using the “no admixture” option. Thinning the dataset as described was necessary to reduce the likelihood of having many SNPs in linkage disequilibrium, which is a necessary requirement for the “no admixture” model to be valid. Thinning also has an additional benefit of computational speed. The number of SNPs we used was more or less optimal because adding more SNPs did not result in any gain in analytical power.

We used 10,000 steps in the “burnin” period, and 20,000 steps as the number of MCMC iterations. We ran the simulation over several values for k = number of inferred (ancestral) populations, and obtained the log likelihoods for the fits as shown in Table T.A1.1 below:

Table T.A1.1: The log likelihood values of the fits from *Structure* [R.A1.3] for various values of k=number of inferred (ancestral) populations.

| k = Number of Inferred (Ancestral) Populations | ln P(X|K) = Estimated Log Probability of Fit | Relative Log Probability of Fit |
| --- | --- | --- |
| 1 | -7124377.3 | -382966.4 |
| 2 | -6792102.1 | -50691.2 |
| 3 | -6756124.2 | -14713.3 |
| 4 | -6752204.2 | -10793.3 |
| 5 | -6769565.8 | -28154.9 |
| 6 | -6741410.9 | 0 |
| 7 | -7247376.2 | -505965.3 |
| 8 | -7242335.3 | -500924.4 |

This analysis shows that k=6 populations is overwhelmingly the most likely fit to the data under the no admixture model. We then used *distruct* [R.A1.4] to create the images shown in Figures F.A1.1a and F.A1.1b. The ~20% European admixture in ASW is clearly visible. It is also clear that the Maasai are highly admixed, with a clear African admixture from YRI and LWK as well as a 15% admixture with two other populations not represented in HapMap.

Table T.A1.2: The percentage contributions to CEU, ASW, LWK, MKK and YRI from the 6 ancestral population groups inferred by STRUCTURE. The ancestral groups are labeled as different colors, indicated on Figure F.A1.

| HapMap Population ID | Teal  (ancestral European) | Brown | Purple | Pink  (ancestral Yoruban) | Green | Yellow | Number of founders |
| --- | --- | --- | --- | --- | --- | --- | --- |
| CEU | 0.998 | 0 | 0 | 0 | 0 | 0 | 112 |
| ASW | 0.202 | 0.003 | 0.002 | 0.759 | 0.026 | 0.009 | 53 |
| LWK | 0.001 | 0.004 | 0.002 | 0.655 | 0.32 | 0.018 | 110 |
| MKK | 0.02 | 0.118 | 0.028 | 0.101 | 0.046 | 0.687 | 156 |
| YRI | 0.001 | 0.002 | 0.001 | 0.992 | 0.003 | 0.002 | 147 |

Figure F.A1.1a: The result of the *Structure* analysis for CEU, ASW, LWK, MKK and YRI. Columns are samples and rows show population admixture. The six colors represent the k=6 ancestral populations for which the fits were optimal.


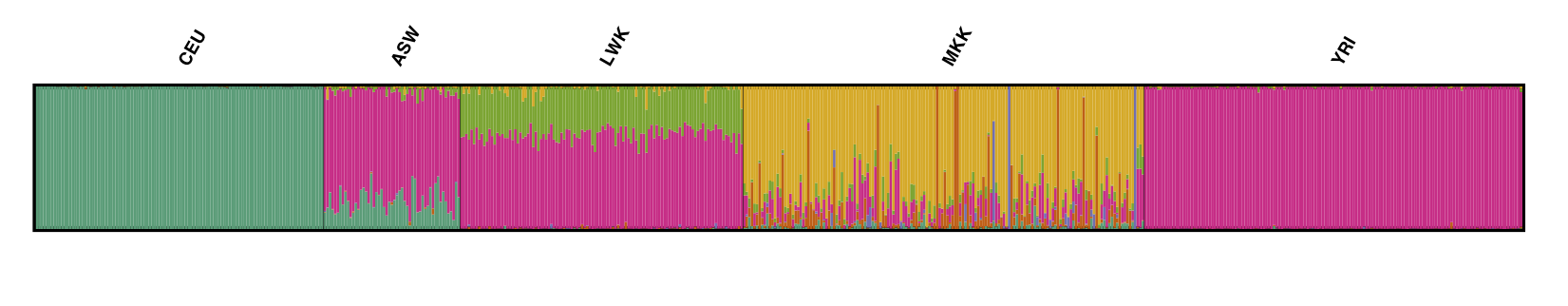


Figure F.A1.1b: The same data as Figure F.A1.1a is shown here but it is pooled over samples within each population to illustrate fractional admixture of k=6 populations within each of them.


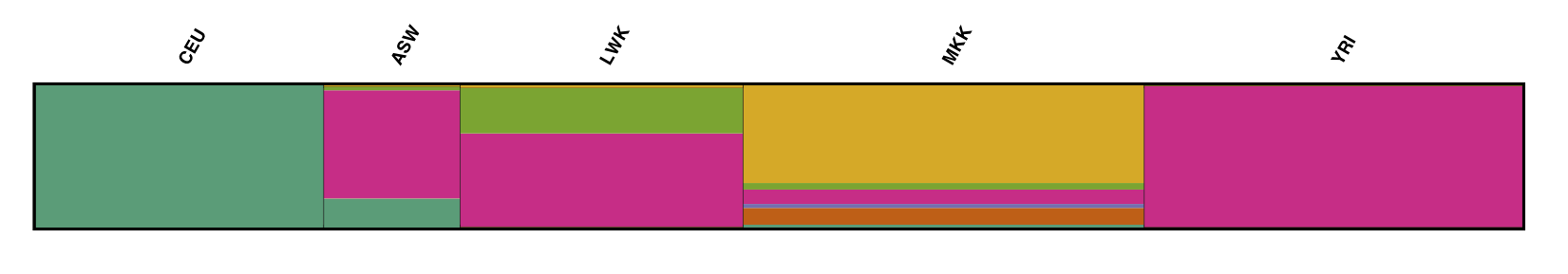


**References for Appendix S:**

[R.A1.1] <http://pngu.mgh.harvard.edu/~purcell/plink/>

[R.A1.2] The International HapMap 3 Consortium. *Integrating common and rare genetic variation in diverse human populations*. Nature 467: 52-58 (2010).

[R.A1.3] Pritchard J K, et al. Inference of population structure using multilocus genotype data. Genetics (2010) <http://pritch.bsd.uchicago.edu/structure.html>
[R.A1.4] http://rosenberglab.bioinformatics.med.umich.edu/distruct.html
