# Supplementary material for: Lactase Persistence and Lipid Pathway Selection in the Maasai
Source: PLoS One. 2012 Sep 28;7(9):e44751. doi: 10.1371/journal.pone.0044751 (PMC3461017; doi:10.1371/journal.pone.0044751)
Supplement: Appendix S2 — Details of Fst calculation, p-values and SNP clustering for Fst and XP-EHH. (DOC) [file pone.0044751.s009.doc]

**Appendix S2: Details of Fst calculation, p-values and SNP clustering for Fst and XP-EHH.**

The overall workflow used in the Fst analysis is shown in Figure F.A2.1. Genotype data from HapMap 3 release 3 [R.A2.1, R.A2.2] was downloaded and pruned to 1,175,055 autosomal SNPs with minor allele frequency > 0.05 in MKK (n = 143 founders) and LWK (n = 100 founders). To reduce the chance of incorporating SNPs with genotyping errors, we imposed a Hardy-Weinberg equilibrium p-value cutoff < 0.05 in either population (as calculated by PLINK [R.A2.3]), and excluded SNPs with genotype missing in > 5% of samples. Fst was computed using the method of Reynolds, Weir and Cockerham [R.A2.4].


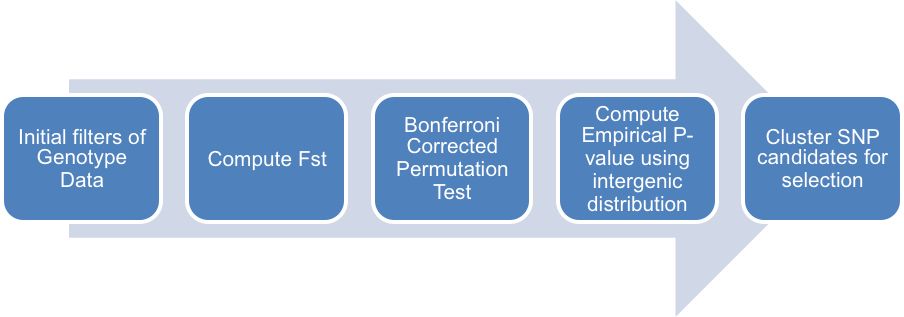


Figure F.A2.1: Workflow illustrating the stages in the Fst analysis of the genotype data

***Fst computation details (summarized from [R.A2.4]):*** If *n_1_* and *n_2_* are the number of MKK and LWK individuals measured at a locus l, and *p_1_* and *p_2_* are the derived allele frequencies at this locus in the two populations, define *a_l_* and *b_l_* as:

*a_l_ = (p_1_-p_2_)^2^ – [(n_1_+n_2_) (2n_1_p_1_(1-p_1_) + 2n_2_p_2_(1-p_2_)]/[4n_1_n_2_(n_1_+n_2_-1)] Eq A.2.1*

*b_l_ = [2n_1_p_1_(1-p_1_)+2n_2_p_2_(1-p_2_)]/(n_1_+n_2_-1) Eq A.2.2*

Then:

*Fst = a_l_ /(a_l_ + b_l_ ) Eq A.2.3*

***P-value computations:***

***1.*** ***Exact Bonferroni corrected permutation p-value p_B_ for Fst***

At every SNP we compute a p-value for Fst using a permutation test, which we can calculate exactly. The null hypothesis is that all rearrangements of the alleles among the two populations are equally probable. The Bonferroni corrected permutation p-value p_B_ is then n times the probability that the value of Fst in the null-distribution exceeds the observed value of Fst, where n = number of hypotheses (SNPs) tested. The details of this calculation are as follows:

For each SNP, there are *2(n_1_+n_2_)* alleles in the combined population. We define a partition of the data by assigning *2n_1_* alleles to MKK and the rest to LWK. The permutation p-value *p(x)* is the sum, over all such partitions, of the probability that the Fst value obtained in a partition is greater than or equal to the Fst value *x* obtained in the actual data. Thus,

*p(x) = Σ_part_ Prob(Fst(part)>x) Eq A.2.4*

Let *n_1_(--),n_2_(--)* and *n_1_(-+),n_2_(-+)* be the number of mutant homozygous and heterozygous individuals in the MKK and LWK cohorts. Then the total number of mutant alleles *N* in the combined population is given by:

*N = 2(n_1_(--)+n_2_(--)) + n_1_(-+)+n_2_(-+) Eq A.2.5*

Since we know the genotypes of the samples, *N* is known from the data. In the 2*n_1_* alleles assigned to MKK, let there be *n* mutant alleles. Then, *p_1_ (part) = n/(2n_1_)* and *p_2_(part) = (N-n)/(2n_2_).* Using these values of *p_1_(part)* and *p_2_(part)* (which are specific to the partition), one can compute *Fst(part)* using the formulae in the previous section. The values that *n* take are limited to the open interval: *[n_min_,n_max_]*, with *n_min_ = max(0,N-2n_2_)* and *n_max_ = min(2n_1_,N)*. Hence, we can rewrite *Eq A.2.4* as:

*p(x) = Σ_n=[nmin,nmax]_ {^N^C_n_ ^(L-N)^C_L1-n_ }/ ^L^C_L1_ | fst(n)>x Eq A.2.6*

where, *L = 2n_1_+2n_2_*, *L1 = 2n_1_* and *L2 = 2n_2_*. The factor in curly brackets *{}* is the number of ways of assigning *n* mutant alleles and N-n non-mutant alleles to the *2n_1_* loci in the MKK samples. The normalization factor in the denominator accounts for all possible ways of choosing *2n_1_* alleles from *2(n_1_+n_2_)* alleles. For each of the 1,175,055 SNPs tested, we computed the sum in *Eq A.2.6* to obtain the permutation p-value *p(x)* for each measured value of Fst = *x*. From this, we obtained a Bonferroni corrected p-value: p_B_*(x) = p(x)*1175055.*

***2. Empirical p-value p_E_ using the Fst distribution of intergenic-genic SNPs***

As a further filter, the Fst values of SNPs in non-intergenic regions were compared to the Fst distribution of intergenic SNPs. SNP annotations were obtained for version hg19 of the human genome from (<http://hgdownload.cse.ucsc.edu/goldenPath/hg19/database/knownGene.txt.gz>). Intergenic SNPs were defined as those located more than 50 Kb away from the start and stop sites of all known genes. This 50Kb buffer was used to exclude promoter regions (possibly conserved due to purifying selection) and to minimize the effect of LD with genic SNPs.

The Fst distribution of 351,254 intergenic SNPs was used to compute an empirical p-value *p_E_* for the remaining non-intergenic SNPs as the probability of finding an intergenic SNPs with higher Fst. **1,232 SNPs within genes or within 50 kb of genes with *pB <* *8.6E-6* and *p_E_* *<0.001* were retained for clustering**. These are shown in Supplementary Table 3a.

***Clustering and population identification:***

***3. Clustering significant SNPs using Linkage Disequilibrium***

The SNPs thus identified as selection candidates do not all represent independent selection events. During a selective sweep, many neighboring linked SNPs can hitchhike along with the selected allele, and thus show correspondingly high scores for selection. In order to identify such linked regions in which high Fst SNPs occur, the 1,232 SNPs identified above were clustered into contiguous genomic regions using genotypic R^2^ in MKK as a measure of linkage disequilibrium. For each population, a SNP was assigned to a cluster if it had a genotype R^2^ >= 0.25 with at least one other SNP in the cluster.

The value R^2^ >=0.25 has been shown to correspond to a genetic distance of 0.01-0.02 cM across a varied set of population growth models [R.A2.5]. Assuming a genomic average recombination rate of 1 cM/Mb, this is equivalent to a physical distance on the order of 10 kb in each direction. The probability for two or more SNPs from a randomly chosen set of 1,232 SNPs to occur within 10kb is close to one percent, hence we conclude that R^2^ >= 0.25 is a reasonably stringent cutoff for linkage.

***4. Using XP-EHH to identify the population in which a sweep has occurred***

Assuming that only one of the two populations has undergone a selective sweep at a given locus, we identified the population in which the sweep is more likely to have occurred by comparing the local haplotype diversity across populations. Concretely, for each cluster identified by Fst, we label it as selection candidate in MKK if the maximum normalized XP-EHH score of a SNP in the cluster is > 3. A positive value for XP-EHH indicates that the MKK carry the longer-range haplotypes.

This procedure identified 26 clusters (containing 318 SNPs) as selection candidates in MKK (Supplementary Table 1a). 9 of these clusters include SNPs that exceed the genome-wide significance threshold for XP-EHH (XP-EHH > 4.79580, Bonferroni corrected p < 0.05, two-tailed). In Table 1, we list the intersection of clusters that are identified as genome-wide significant by at least two out of the three methods used (Fst, iHS, XP-EHH). Supplementary Table 2 shows the concordance of our results with those of the HapMap consortium [R.A2.1].

The remaining SNPs were either singletons (did not occur in clusters) or were in clusters that could not be confidently assigned to the MKK. In Table 2, we list the non-synonymous SNPs with most significant genome-wide Fst. These are our top candidates for possible functional polymorphisms. The validation test of the association of these SNPs in the Maasai with protection against hyperlipidemia and atherosclerosis remains an open question. This issue can only be resolved by a prospective field study (measurement of cholesterol levels and polymorphisms in Maasai) or the analysis of data collected within a relevant biological/environmental context. One possibility is to do an association study of these polymorphisms with lipid levels or atherosclerotic index in Maasai under treatment for heart disease (case group) vs Maasai under treatment for other diseases not related to lipidemia (control group) in hospitals.

**References for Appendix 2:**

[R.A2.1] The International HapMap 3 Consortium. *Integrating common and rare genetic variation in diverse human populations*. Nature 467: 52-58 (2010).

[R.A2.2] <http://snp.cshl.org/>

[R.A2.3] <http://pngu.mgh.harvard.edu/~purcell/plink/>

[R.A2.4] Reynolds J, Weir BS, Cockerham CC, *Estimation of the coancestry coefficient: basis for a short-term genetic distance.* Genetics, 105(3): 767-779 (1983).

[R.A2.5] Pritchard JK, Przeworski M, Linkage disequilibrium in humans: models and data. Am J Hum Genet, 69(1):1-14 (2001).
