# Supplementary material for: Lactase Persistence and Lipid Pathway Selection in the Maasai
Source: PLoS One. 2012 Sep 28;7(9):e44751. doi: 10.1371/journal.pone.0044751 (PMC3461017; doi:10.1371/journal.pone.0044751)
Supplement: Appendix S3 — Details of iHS calculation. (DOC) [file pone.0044751.s010.doc]

**Appendix S3: Details of iHS calculation**

Selection events not only sweep functional loci to high frequency, they also reduce haplotype diversity in the region around the selected locus because of hitchhiking. The Extended Haplotypic Homozygosity (EHH) statistic [R.A3.1] exploits this principle and provides a criterion for detecting SNPs under selection within a population *without* reference to another population. EHH measures the diversity of extended haplotypes containing a chosen core haplotype, as a function of the distance from the core. EHH is defined as the probability, as a function of distance from the core region, that two randomly selected haplotypes that share the core, will be identical. Recently, much work has focused on using EHH to define various measures (such as iHS [R.A3.2] or iES [R.A3.3]) which can identify recent selection. Here, we use iHS to detect recent selective sweeps in the Maasai.

***The iHS statistic:***

iHS is defined as the log of the ratio of the integrated EHH score for haplotypes containing the ancestral allele (the allele under selection) to the integrated EHH score for haplotypes containing the derived allele [R.A3.2]. Since iHS is a local measure and does not use a reference population, haplotypes for both alleles share the same genomic environment and are subject to identical population demography. Hence iHS does not suffer from ambiguities associated with genomic and population structure specific variations such as differences in recombination rates, demographic history, population bottlenecks etc. As a result, significantly large values of iHS are more likely to be due to selection.

High values of iHS occur when haplotype diversity is reduced because of selection induced hitchhiking (genetic draft), which leads to more extended haplotypes for the selected allele and a consequent slower fall-off of EHH on either side of the selected locus. A high iHS scoring SNP typically has one allele (not necessarily derived) which is associated with longer haplotypes and lower neighborhood diversity compared to the other allele (see Figure 1(A) in [R.A3.2] which shows a simulation of a selected locus with a beneficial derived allele). In a selective sweep, hitchhiking causes both functional SNPs as well as any SNPs in their neighborhood to have amplified iHS scores. In fact, simulations show that a high density of high-scoring SNPs is a better indicator of a selective sweep than high iHS score of a single SNP [R.A3.2].

***Computational details and p-value significance***

Autosomal haplotype data phased with IMPUTE++ [R.A3.4] was downloaded on 10.24.2010 from: [http://hapmap.ncbi.nlm.nih.gov/downloads/phasing/2009-02_phaseIII/HapMap3_r2/](http://www.google.com/url?q=http%3A%2F%2Fhapmap.ncbi.nlm.nih.gov%2Fdownloads%2Fphasing%2F2009-02_phaseIII%2FHapMap3_r2%2F&sa=D&sntz=1&usg=AFQjCNH0OY0JnKYDZWNUbAFcrw7Ru6AaJg). SNPs were pre-filtered for Hardy Weinberg equilibrium and had low frequency of Mendel errors (see [http://hapmap.ncbi.nlm.nih.gov/downloads/genotypes/2010-05_phaseIII/00README.txt](http://www.google.com/url?q=http%3A%2F%2Fhapmap.ncbi.nlm.nih.gov%2Fdownloads%2Fgenotypes%2F2010-05_phaseIII%2F00README.txt&sa=D&sntz=1&usg=AFQjCNE_byZ5DSu-k6guG73lIatQW_CYOA)). We further pruned the data by removing SNPs with MAF < 0.1. After applying these filters, we analyzed 991,737 SNPs. The ancestral allele information was downloaded from the NCBI ftp server ( <ftp://ftp.ncbi.nih.gov/snp/database/shared_data/>) on 7.5.2011. Genetic maps were downloaded from HapMap website (<http://hapmap.ncbi.nlm.nih.gov/downloads/recombination/2008-03_rel22_B36/rates/> as accessed on 9/22/2010). These maps did not contain the genetic distances for all the SNPs used, and hence we interpolated the genetic distances. For the final results genetic distances were converted to GRCh37.

To identify potential genomic regions under selection in the MKK data, we followed the protocol described in [R.A3.2]. We calculated the integrals of EHH for each SNP using the genetic distances over a domain of integration such that atleast one allele of the SNP has an EHH > 0.05. We then binned these raw scores according to derived allele frequency.
Since the total number of haplotypes is 286, the frequencies of alleles do not form a continuous spectrum but are integral multiples of 1/286 (=0.0035). Hence, we used each frequency to comprise a single bin, and got 229 bins spanning frequencies 0.1 to 0.9 (SNPs with minor allele frequency < 0.1 were filtered out). The number of points in each bin ranged from 9920 to 1579. We computed the mean and standard deviation of iHS values for each frequency bin and standard normalized the raw iHS scores to mean zero and variance unity. We then looked at sliding windows of 50 consecutive SNPs and noted the fraction of SNPs with normalized-iHS (simply called iHS from now on) ≥ 2. This fraction of high-scoring SNPs in a window is the statistic used for detecting selective SNPs [R.A3.2]. Using sliding windows is advantageous over using fixed, gene-centric windows as done in [R.A3.2], since we can locate a strong signal in an unbiased way. In [R.A3.2], the top 1% of non-overlapping windows were candidates for selective sweep. Since here we used sliding windows of size 50 SNPs, we corrected for this by looking at 1/50th of the top 1% of such windows with the constraint that they do not overlap. Overall we analyzed 990,659 windows and chose top 196 non-overlapping windows. Some of these chosen windows were adjacent to each other, and are likely to represent sweeps extending more than 50 SNPs. To merge such windows, we listed all SNPs with |iHS| ≥ 2 (high-scoring SNPs) in these top windows (listed in Supplementary Table 2b), and clustered them using a genotype R^2^ cutoff of 0.5. This clustering has the advantage that it does not impose an ad-hoc window size, but is based on local patterns of LD. We also noticed that some windows had high-scoring SNPs that formed two or more smaller clusters. Since these clusters of SNPs are not in LD with each other, they do not belong to the same sweep. This means that these windows were selected erroneously (the rationale behind selecting windows with high density of high-scoring SNPs was that they all belong to the same selective sweep). We kept clusters with size greater than the least number of high-scoring SNPs in the top windows (this number turns out to be 20). These clusters are candidate regions for selective sweeps in the Maasai, and are given in Supplementary Table 2b. We then used the UCSC genome browser to identify genes and Genome-Wide Association Studies' (GWAS) SNPs which lie in these regions.

**References for Supplementary Appendix 3:**

[R.A3.1] Sabeti, P. C. *et al.* Detecting recent positive selection in the human genome from haplotype structure. *Nature* **419**, 832-837 (2002).

[R.A3.2] Voight, B. F., Kudaravalli, S., Wen, X. & Pritchard, J. K. A map of recent positive selection in the human genome. *PLoS biology* **4**, e72+ (2006).

[R.A3.3] Tang, K., Thornton, K. R. & Stoneking, M. A new approach for using genome scans to detect recent positive selection in the human genome. *PLoS biology* **5**, e171+ (2007).

[R.A3.4] Howie, B. N., Donnelly P., Marchini J. A Flexible and Accurate Genotype Imputation Method for the Next Generation of Genome-Wide Association Studies. *PLoS Genet* **5**, e1000529 (2009).
