# Supplementary material for: Lactase Persistence and Lipid Pathway Selection in the Maasai
Source: PLoS One. 2012 Sep 28;7(9):e44751. doi: 10.1371/journal.pone.0044751 (PMC3461017; doi:10.1371/journal.pone.0044751)
Supplement: Appendix S4 — Details of XP-EHH calculation. (PDF) [file pone.0044751.s011.pdf]

Appendix S4: Details of XP-EHH calculation

A selective sweep results in the rapid rise in the frequency of beneficial alleles accompanied by a reduction in haplotype diversity in the neighborhood of functional mutations due to a "hitch-hiking" effect (for example, see [R.S4.1] for a discussion). The key idea behind methods to identify selective sweeps is to use metrics that probe such reduced haplotype diversity. The statistic EHH (Extended Haplotype Homozygosity) [R.S4.2] is one such metric. It measures the reduction in haplotype diversity by computing the probability that two extended haplotypes around a given locus are the same, given that they have the same allele at the locus. While selection decreases haplotype diversity, recombination increases it. Since recombination rates vary widely across the genome within and between populations, the EHH statistic can be interpreted as a measure of selection only after suitable normalization. The iHS statistic [R.S4.3] compares the integrated EHH profiles *b*etween two alleles at a given SNP in the same population (iHS is discussed in more detail in Supplementary Appendix 3). On the other hand, the XP-EHH (Cross Population Extended Haplotype Homozygosity) statistic (defined below) compares the integrated EHH profiles *b*etween two populations at the same SNP [R.S4.4].

The iHS statistic is expected to be more reliable when one cannot find a good reference population (i.e. when the demographic history of potential reference populations is unknown or very different from the target population), but has low power when the selected allele is close to fixation. On the other hand, XP-EHH is expected to be more reliable if a reference population with a similar demographic history is available, and if the allele under selection is close to fixation in one of the populations. For XP-EHH, we used the Luhya (LWK) samples as the reference population to compare to the Maasai (MKK) samples. The motivation to choose the LWK samples was that they were closest to MKK with respect to overall population structure (as discussed in the main text).

Computing XP-EHH requires the computation of EHH in each population. For a bi-allelic SNP with alleles a and A, the EHH is defined as follows:

$$EHH(x) = \frac{\sum_{i=1}^{h_x} \binom{n_i}{2}}{\binom{n_a}{2} + \binom{n_A}{2}} \tag{1}$$

Here n_a and n_A are the number of haplotypes with alleles a and A respectively, n_i is the count of the ith haplotype in a population and h_x represents the number of distinct haplotypes in a genomic region up to a distance x from the locus. The unstandardized XP-EHH statistic is then defined as:

$$unstandardized\ XP - EHH = \log\left(\frac{\int_D EHH_{pop1}(x)dx}{\int_D EHH_{pop2}(x)dx}\right) \tag{2}$$

In Eq. (2), $pop1$ and $pop2$ represent the two populations ($pop1$ = MKK and $pop2$ = LWK in our case). The integration domain D (cutoff over the x integration) was chosen so that

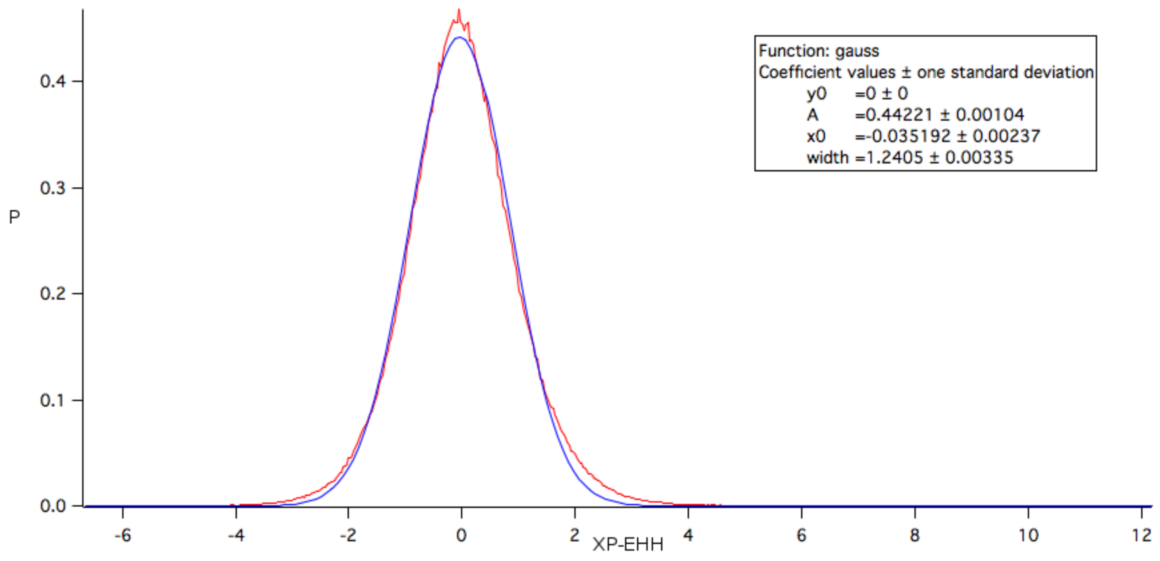

Figure 1: Distribution of normalized XP-EHH scores (red) with the Gaussian fit (blue). The Gaussian function used is $P(x) = A\exp((x-x_0)^2/width^2)+y_0$, the values for the parameters are given in the legend.

the EHH values for both populations have fallen to sufficiently small values. We chose the cutoff as the distance at which EHH for both the populations combined was $0.03-0.04$. The unstandardized XP-EHH scores from Eq. (2) were standard normalized and p-value cutoffs were obtained (after correcting for multiple hypothesis testing) from a Gaussian fit to the resulting data. Since XP-EHH (unlike iHS) is not sensitive to allele frequencies, there is no need to stratify the data into frequency bins before determining significance.

Autosomal haplotype data phased with IMPUTE++ was downloaded on 10.24.2010 from: `http://hapmap.ncbi.nlm.nih.gov/downloads/phasing/2009-02_phaseIII/HapMap3_r2/`. SNPs were pre-filtered for Hardy Weinberg equilibrium and had low frequency of Mendel errors (see `http://hapmap.ncbi.nlm.nih.gov/downloads/genotypes/2010-05_phaseIII/00README.txt`). Common SNPs between the MKK and the LWK were retained, and unstandardized XP-EHH scores were computed using the program by Joe Pickerell at `http://hgdp.uchicago.edu/Software/`. SNPs with unique positions in the dbSNP build 131 (GRCh37) were retained, leaving 1,373,756 SNPs. As expected, the distribution of XP-EHH was close to Gaussian (Fig. 1). We used IGOR Pro (`http://www.wavemetrics.com/products/igorpro/igorpro.htm`) to fit the data to a Gaussian, using the Levenberg-Marquardt method for curve-fitting. Using this fit, we obtained the cutoff of XPEHH > 4.7958 at 95% genome-wide significance levels (two-tailed Bonferroni corrected $p = 0.05$, $n = 1,373,756$). SNPs passing this threshold are candidates for selection in MKK and are listed in Supplementary Table 3a. High scoring XP-EHH SNPs seemed to naturally form clusters when mapped to chromosomal regions. To identify regions associated with selective sweeps, high scoring SNPs were clustered using the same scheme as was used for Fst and iHS. Clusters of SNPs were defined as sets of SNPs that had genotype $R^2 \geq 0.25$ for at least two SNPs in the cluster. These clusters of SNPs are listed in Supplementary Table 3b.

References

[R.S4.1] Gillespie J H (1998) *Population Genetics: A Concise Guide* (Johns Hopkins Univ. Press, Baltimore).

[R.S4.2] Sabeti, P.C. et al. (2002) Detecting recent positive selection in the human genome from haplotype structure. Nature, 419, 832-837.

[R.S4.3] Voight, B.F. et al. (2006) A Map of Recent Positive Selection in the Human Genome L. Hurst, ed. PLoS Biology, 4(3), p.e72.

[R.S4.4] Sabeti, P.C. et al. (2007) Genome-wide detection and characterization of positive selection in human populations. Nature, 449(7164), 913-918.
