# Supplementary material for: Lactase Persistence and Lipid Pathway Selection in the Maasai
Source: PLoS One. 2012 Sep 28;7(9):e44751. doi: 10.1371/journal.pone.0044751 (PMC3461017; doi:10.1371/journal.pone.0044751)
Supplement: Appendix S5 — Details of Sequencing in LCT/MCM6 locus (DOC) [file pone.0044751.s012.doc]

**Appendix S5: Details of Sequencing in LCT/MCM6 locus**

All our methods of detecting selection (Fst, iHS and Allele Frequency Spectrum analysis) suggested that the locus on chromosome 2 containing LCT, MCM6, RAB3GAP1 and other genes was under strong selection in MKK.

Table T.A5.1 shows the SNPs in MCM6 known to be associated with lactase persistence in Africans and Europeans [R.A5.1] and one SNP in RAB3GAP1 that has been associated with total cholesterol levels in a GWAS of >100,000 individuals of European ancestry. To test whether any of these SNPs are segregating in the Maasai, 50 μg of DNA samples for 6 randomly selected MKK samples: (HapMap ids: NA21367, NA21379, NA21454, NA21519, NA21522, NA21650) were purchased from Coriell (http://www.coriell.org/). Forward and reverse primers (shown in Table T.A5.2) for these five regions were identified (using Primer3 [R.A5.3]) and used to perform Sanger sequencing of these regions to infer the genotypes for the six MKK samples. BLAT [R.A5.4] was used to ensure that the primers were unique.

**Table T.A5.1: SNPs genotyped**

| G/C-14010 | MCM6 | Associated with Lactase Persistence in Africans[R.A5.1] |
| --- | --- | --- |
| rs4988235 | MCM6 | Associated with Lactase Persistence in Europeans[R.A5.1] |
| rs41380347 | MCM6 | Associated with Lactase Persistence in Africans[R.A5.1] |
| rs182549 | MCM6 | Associated with Lactase Persistence in Europeans[R.A5.1] |
| rs7570971 | RAB3GAP1 | Associated with Total Cholesterol levels in Europeans[R.A5.2] |

Table T.A5.2: Forward and Reverse Primers used

| Forward Primer | Reverse Primer | SNPs contained in the region |
| --- | --- | --- |
| TGCTCATACGACCATGGAAT | GCAGGGCTCAAAGAACAATC | rs41525747, rs4988235, rs41380347, G/C-14010 |
| GACATGCTGATCAACTATCACAA | AAGAAGTCAGAATACCCCTACCC | rs182549 |
| ATGCACAGGGGTACACACAC | TGGCTTCATTTTAAGCCTGTG | rs7570971 |

The results showed that all but one of the SNPs in MCM6 were wildtype homozygous in all 6 MKK samples. The exception was the SNP G/C-14010 which was found to be variant. Furthermore, SNP rs7570971 in RAB3GAP1 was found to be mutant homozygous. (Wildtype and mutant here being defined with respect to the human genome) Table T.A5.3 summarizes the genotypes at the 6 SNPs.

**Table T.A5.3: Genotypes measured by sequencing**

|  | G/C - 14010 | rs41380347  T/G - 13915 | rs4988235  C/T - 13910 | rs41525747  C/G – 13907 | rs182549  G/A - 22018 | rs7570971 |
| --- | --- | --- | --- | --- | --- | --- |
| NA21367 | CC | TT | CC | CC | GG | AA |
| NA21379 | GC | TT | CC | CC | GG | AA |
| NA21454 | CG | TT | CC | CC | GG | AA |
| NA21519 | CC | TT | CC | CC | GG | AA |
| NA21522 | GG | TT | CC | CC | GG | AA |
| NA21650 | GC | TT | CC | CC | GG | AA |

At the SNP G/C-14010, the genotype of one of the six samples was wild type (GG), three genotypes were heterozygous (GC) and two were mutant homozygous (CC) thus giving the frequency of the C allele to be p=7/12 = 0.58. Using the standard formulae for error in mean due to sampling bias we find

Var(p) = p(1-p)/(2n) = 0.02025 which gives, σ_p_ = Sqrt(Var(p)) = 0.14.

Hence, a 68% confidence interval for p is given by :

p = 0.58 +/- 0.14.

**References**

R.A5.1. Tishkoff et al. Convergent adaptation of human lactase persistence in Africa and Europe. Nature Genetics 39, 31-40 (2007).

R.A5.2 Teslovich TMet al.Biological, clinical and population relevance of 95 loci for blood lipids.Nature,466:707-713 (2010).

R.A5.3. http://frodo.wi.mit.edu/primer3/

R.A5.4. Kent, W. J. BLAT -- The BLAST-Like Alignment Tool. Genome Research 4: 656-664 (2002)

<http://genome.ucsc.edu/cgi-bin/hgBlat>
