# Supplementary material for: Lactase Persistence and Lipid Pathway Selection in the Maasai
Source: PLoS One. 2012 Sep 28;7(9):e44751. doi: 10.1371/journal.pone.0044751 (PMC3461017; doi:10.1371/journal.pone.0044751)
Supplement: Appendix S6 — Plots of Fst, XP-EHH and iHS for all chromosomes. (PDF) [file pone.0044751.s013.pdf]

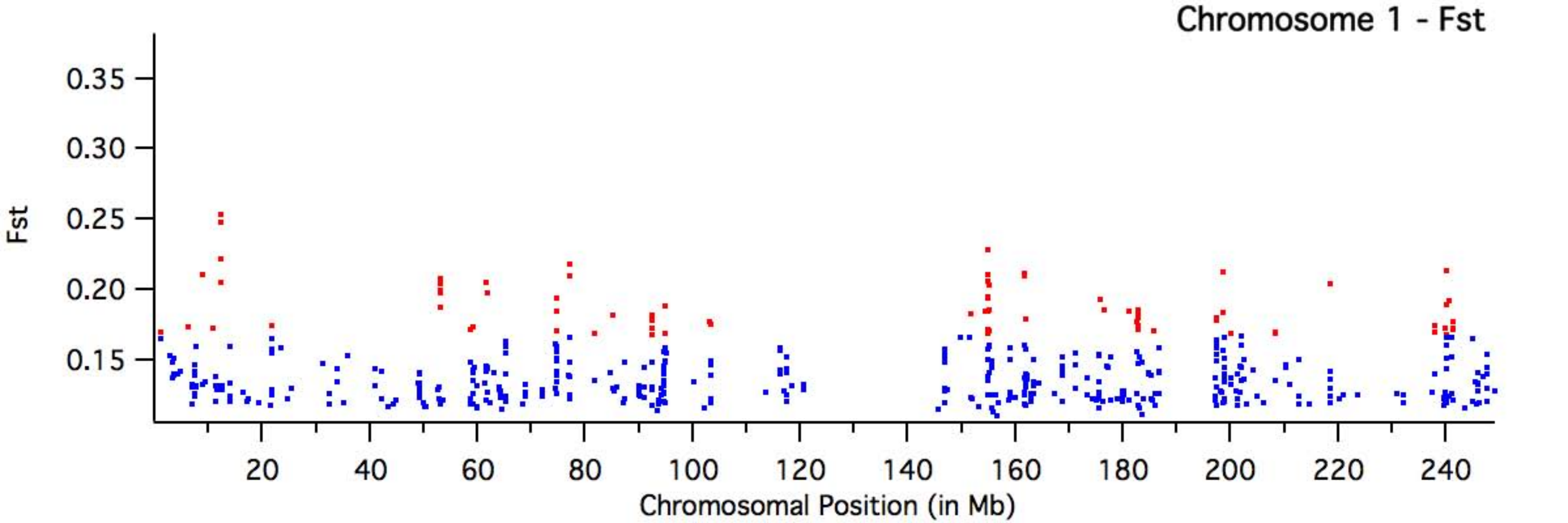

Chromosome 1 - Fst
Fst
0.35
0.30
0.25
0.20
0.15
20
40
60
80
100
120
140
160
180
200
220
240
Chromosomal Position (in Mb)

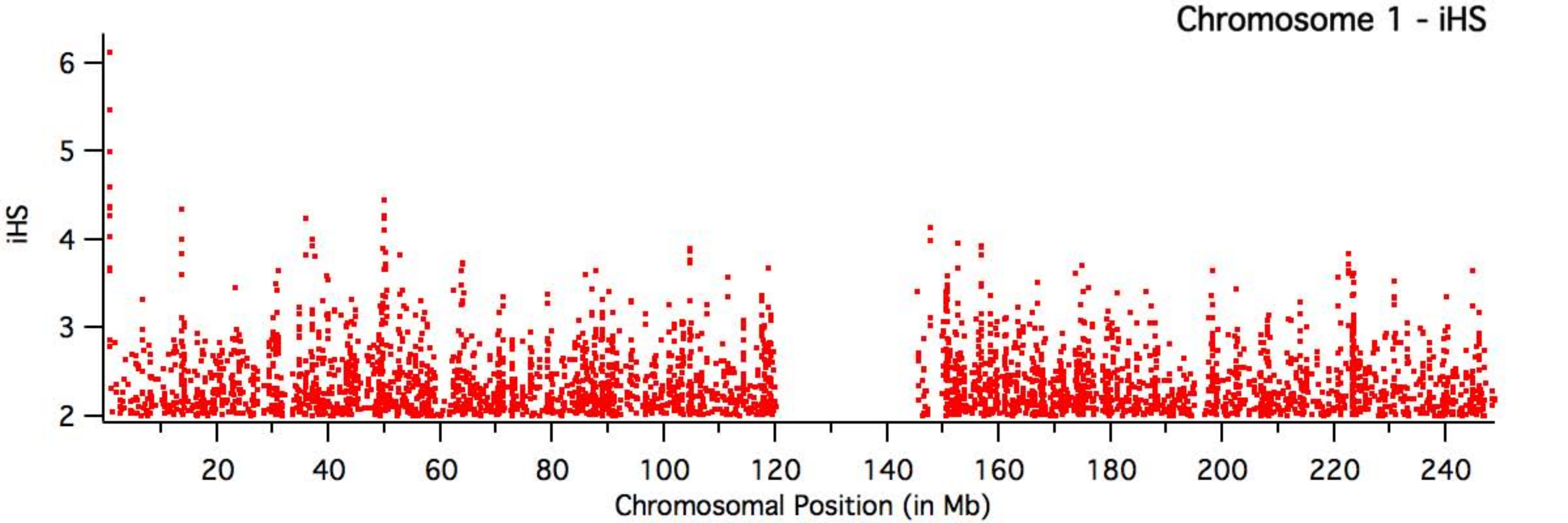

Chromosome 1 - iHS
iHS
6
5
4
3
2
20
40
60
80
100
120
140
160
180
200
220
240
Chromosomal Position (in Mb)

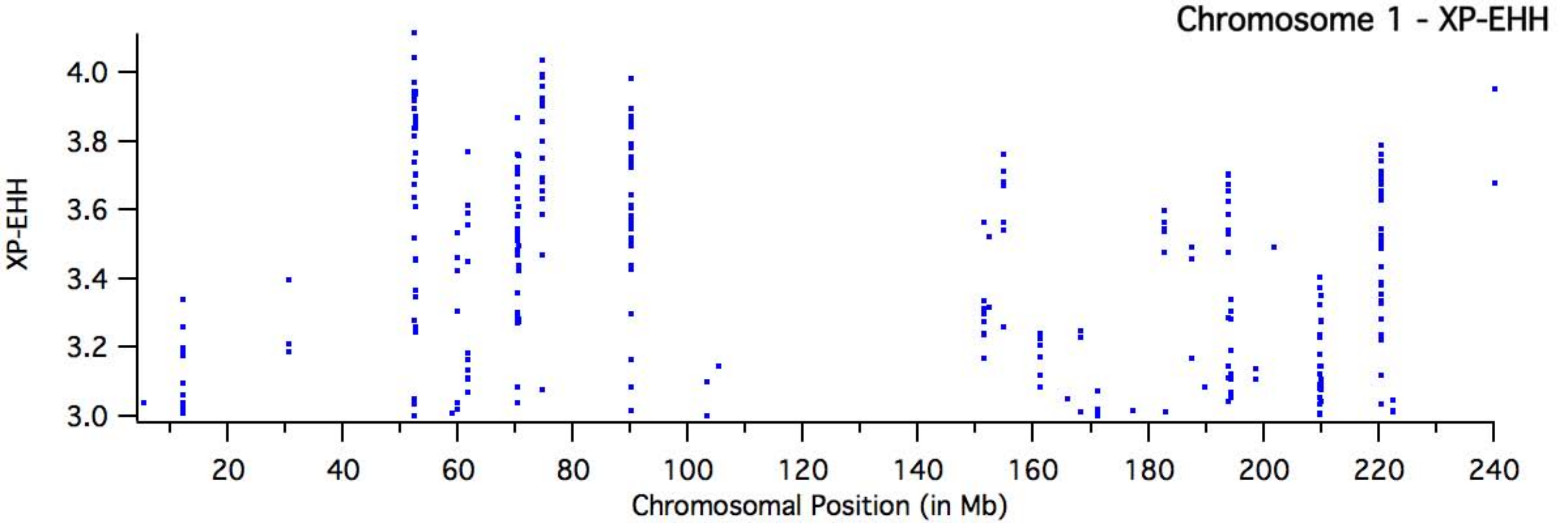

Chromosome 1 - XP-EHH
XP-EHH
4.0
3.8
3.6
3.4
3.2
3.0
20
40
60
80
100
120
140
160
180
200
220
240
Chromosomal Position (in Mb)

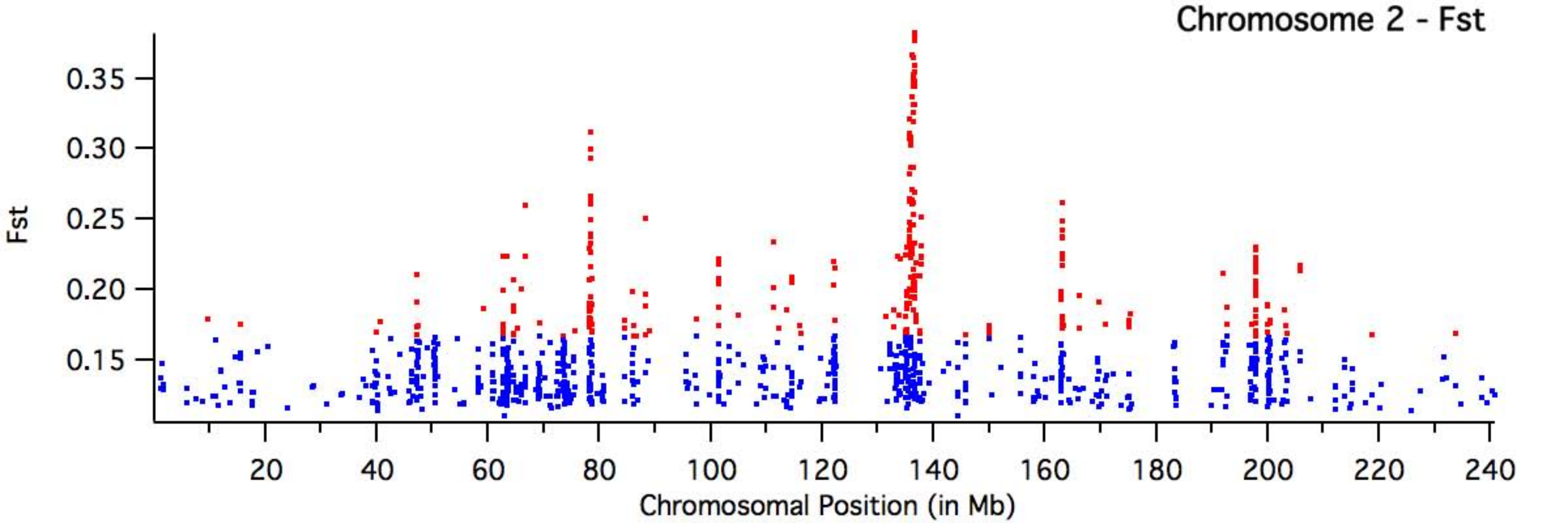

Chromosome 2 - Fst
Fst
0.35
0.30
0.25
0.20
0.15
20
40
60
80
100
120
140
160
180
200
220
240
Chromosomal Position (in Mb)

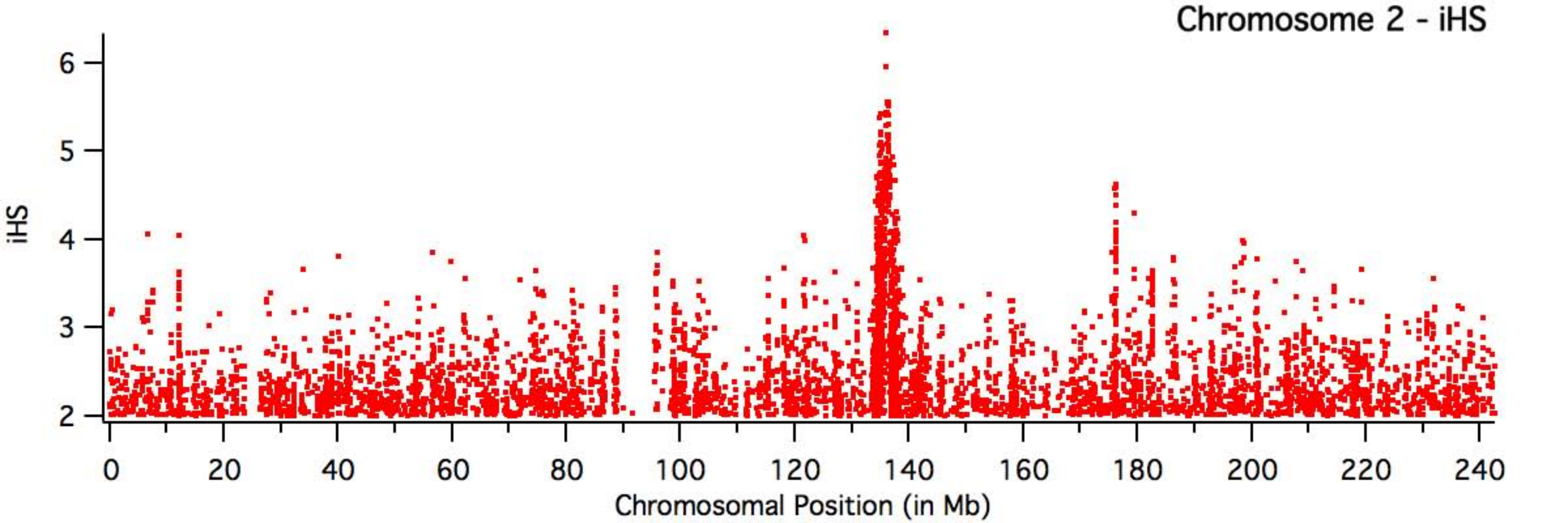
Chromosome 2 - iHS
iHS
6
5
4
3
2
0
20
40
60
80
100
120
140
160
180
200
220
240
Chromosomal Position (in Mb)

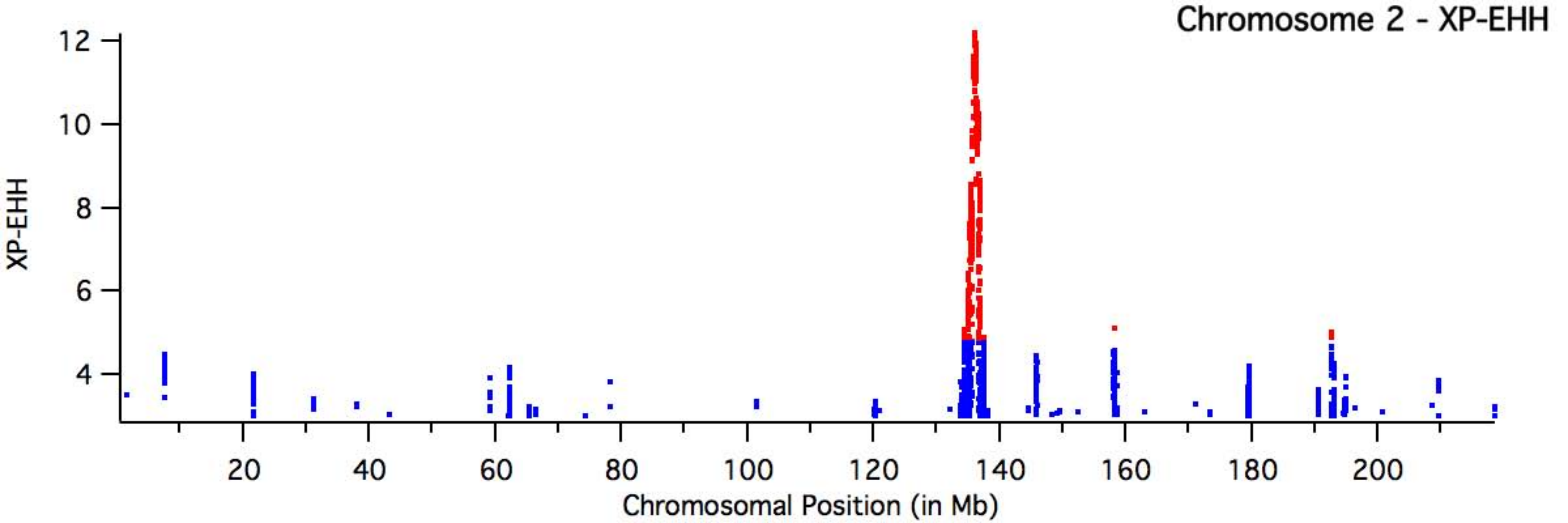
Chromosome 2 - XP-EHH
XP-EHH
12
10
8
6
4
20
40
60
80
100
120
140
160
180
200
Chromosomal Position (in Mb)

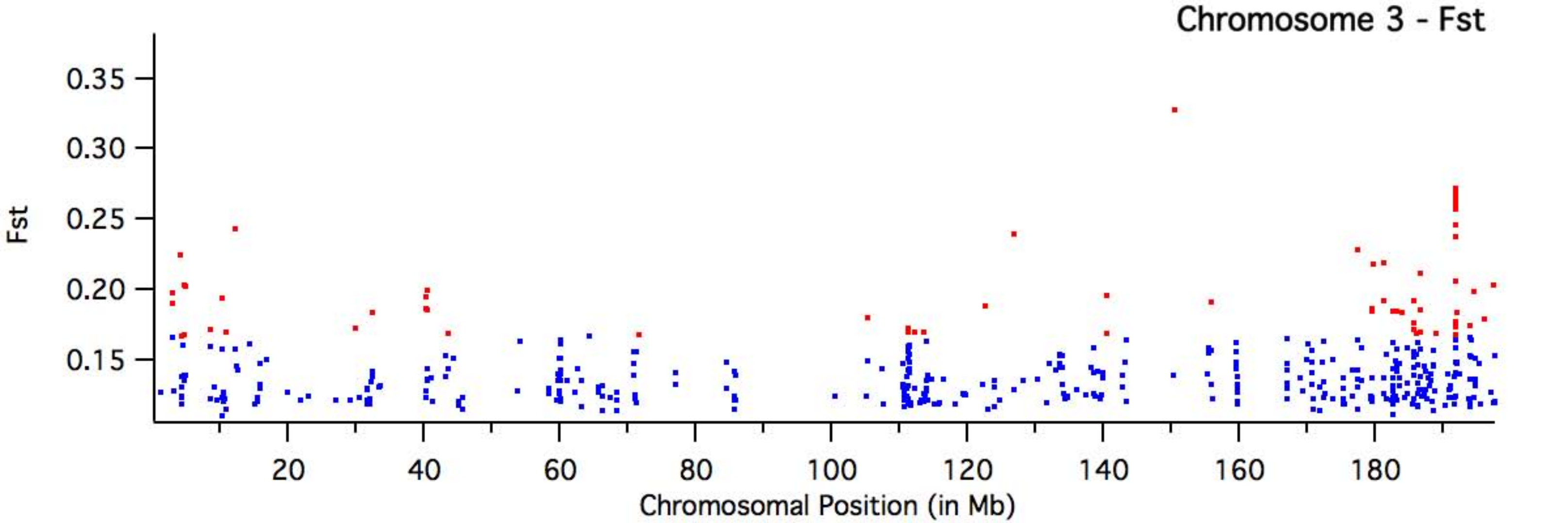
Chromosome 3 - Fst
Fst
0.35
0.30
0.25
0.20
0.15
20
40
60
80
100
120
140
160
180
Chromosomal Position (in Mb)

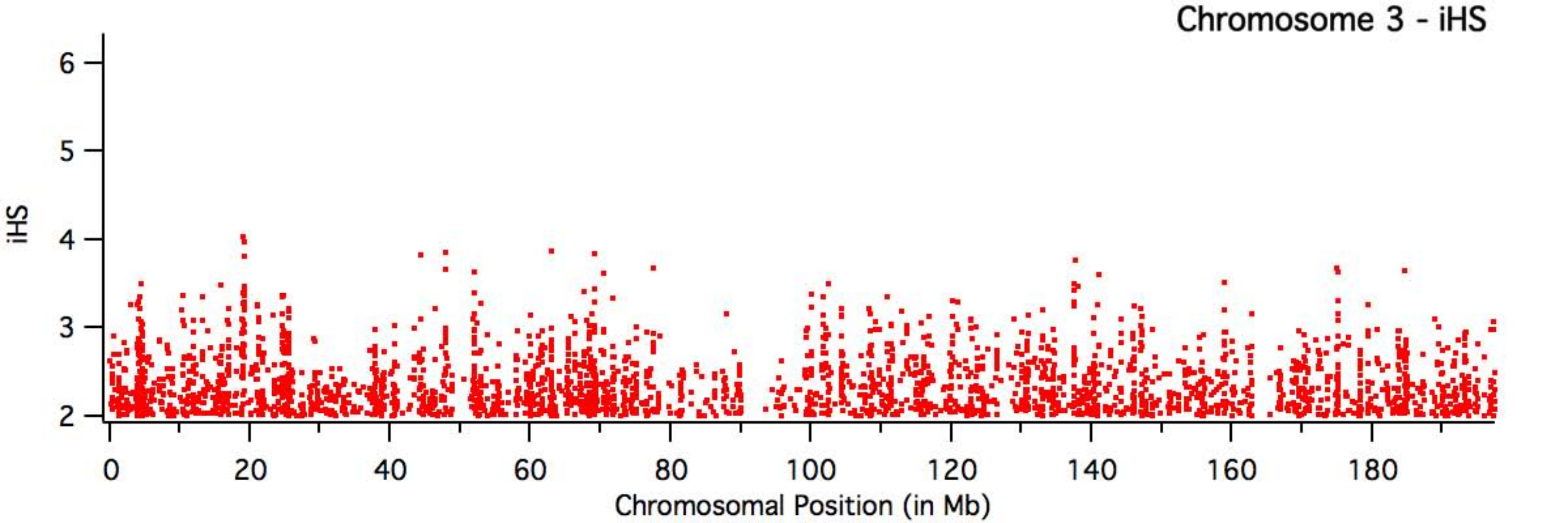
Chromosome 3 - iHS
iHS
6
5
4
3
2
0
20
40
60
80
100
120
140
160
180
Chromosomal Position (in Mb)

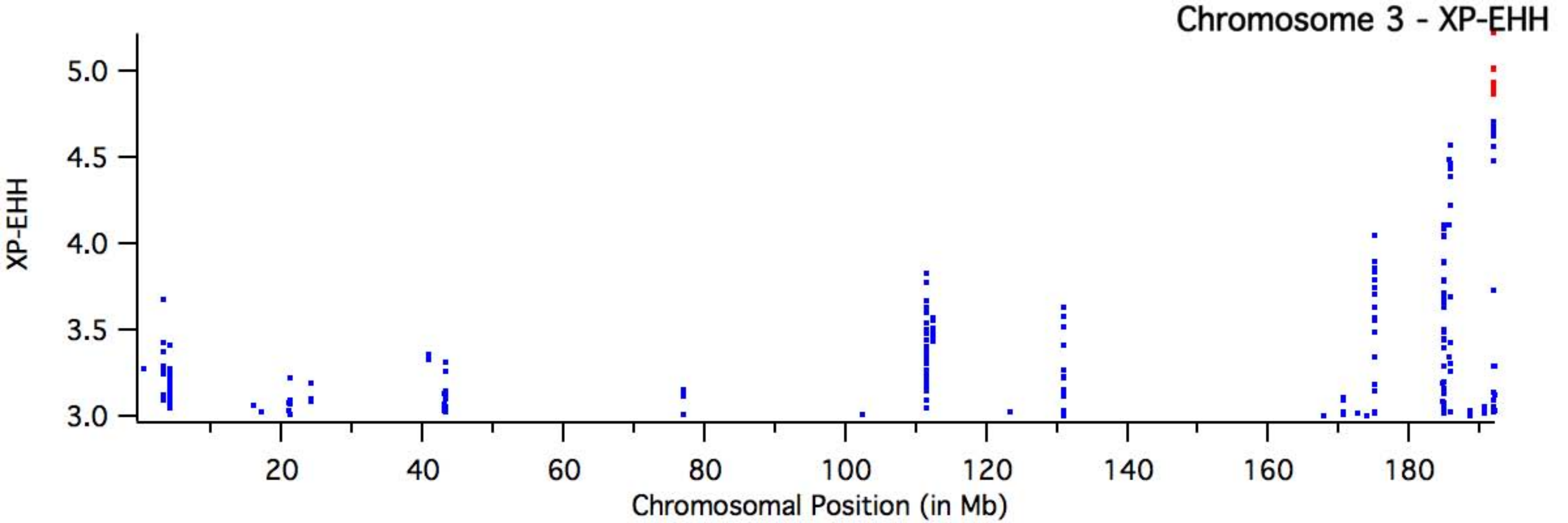
Chromosome 3 - XP-EHH
XP-EHH
5.0
4.5
4.0
3.5
3.0
20
40
60
80
100
120
140
160
180
Chromosomal Position (in Mb)

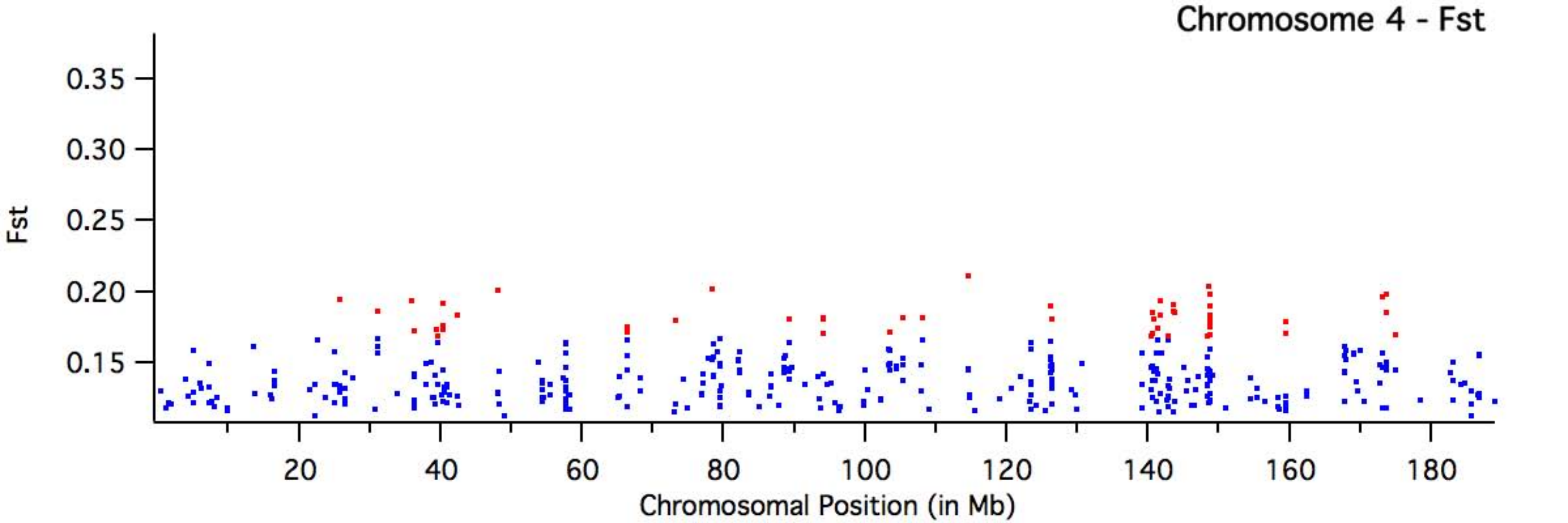
Chromosome 4 - Fst
Fst
0.35
0.30
0.25
0.20
0.15
20
40
60
80
100
120
140
160
180
Chromosomal Position (in Mb)

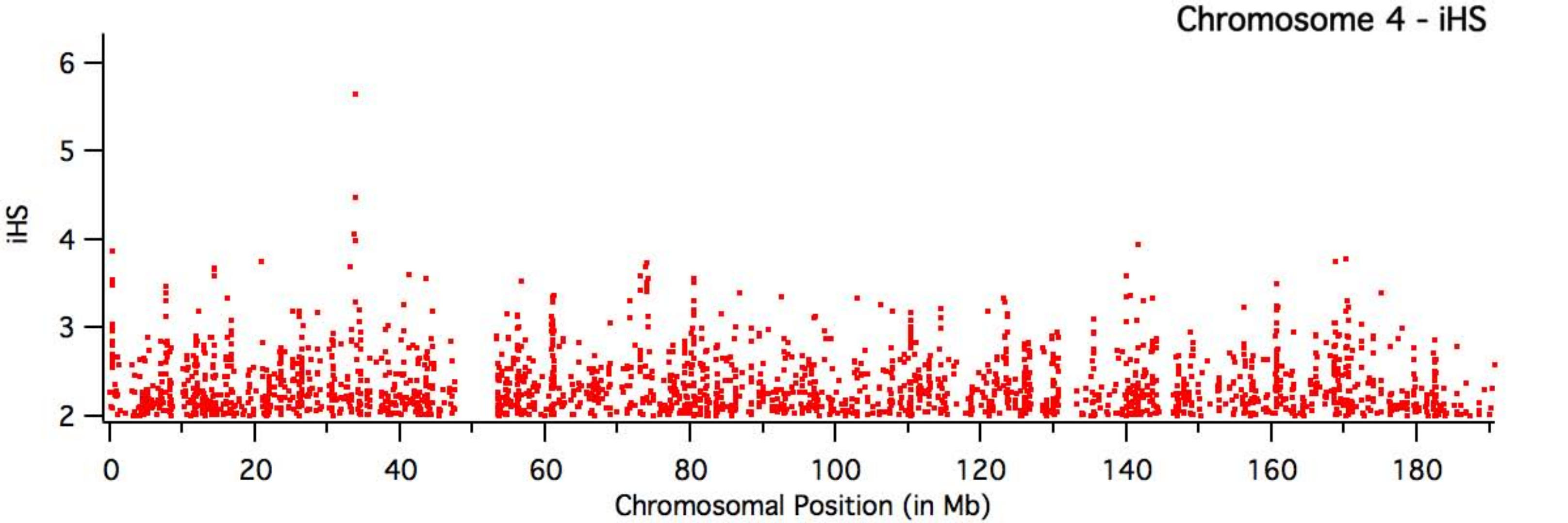

Chromosome 4 - iHS
iHS
6
5
4
3
2
0
20
40
60
80
100
120
140
160
180
Chromosomal Position (in Mb)

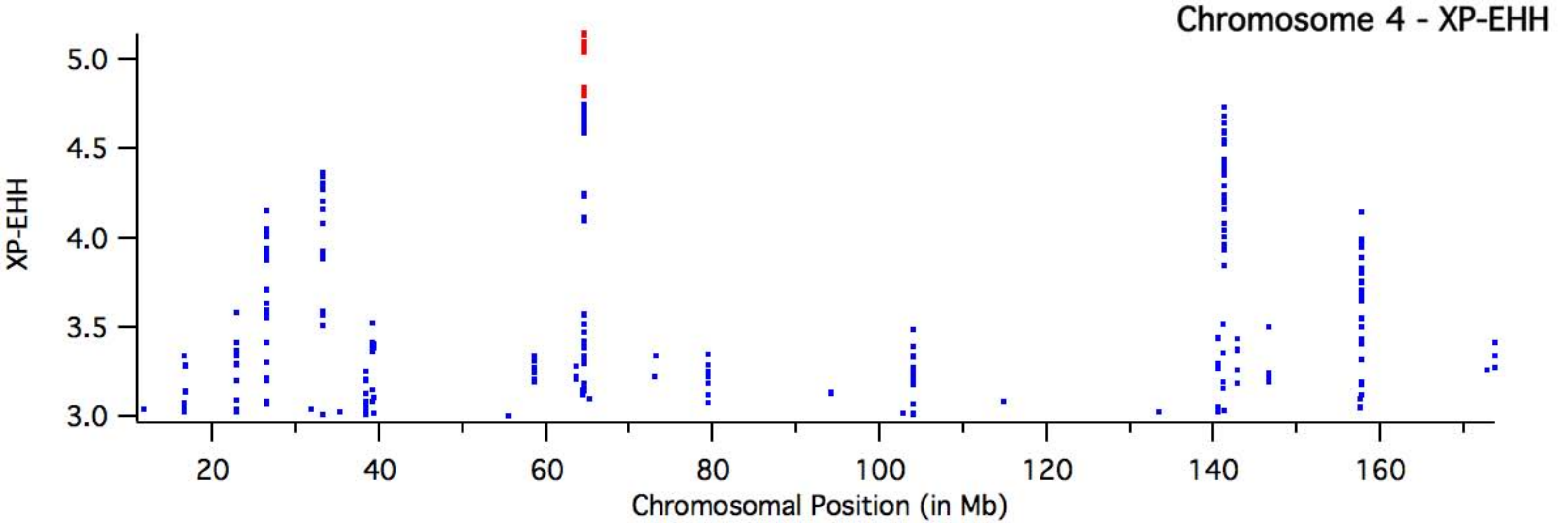
Chromosome 4 - XP-EHH
XP-EHH
5.0
4.5
4.0
3.5
3.0
20
40
60
80
100
120
140
160
Chromosomal Position (in Mb)

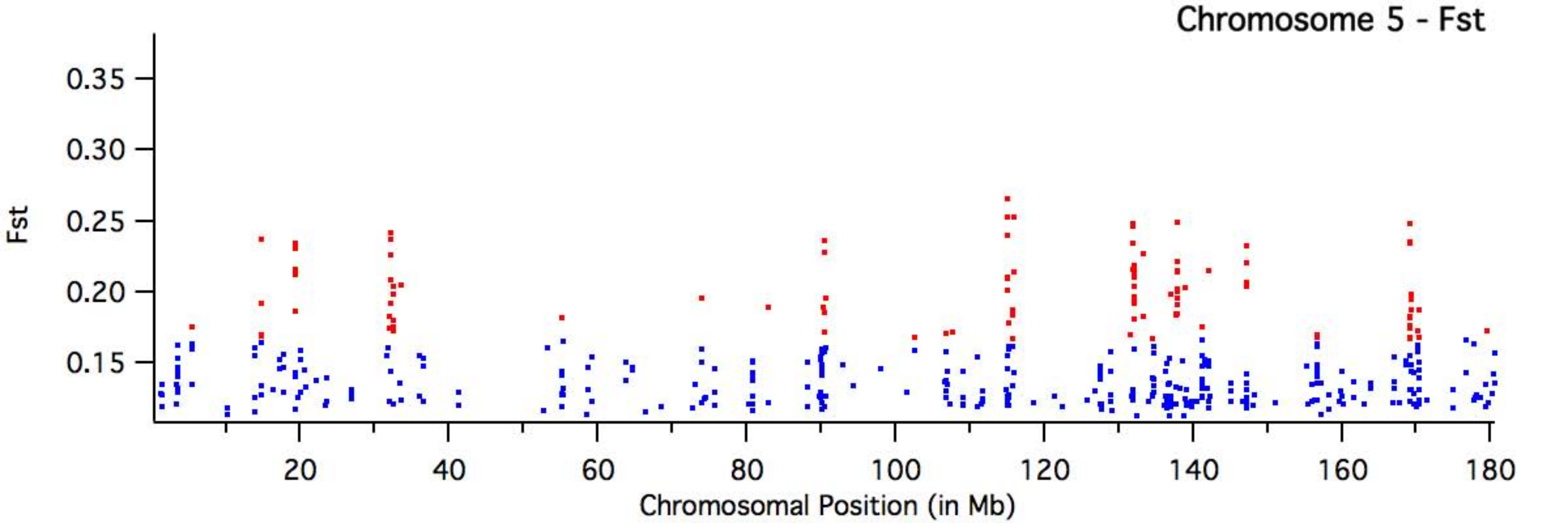
Chromosome 5 - Fst
Fst
0.35
0.30
0.25
0.20
0.15
20
40
60
80
100
120
140
160
180
Chromosomal Position (in Mb)

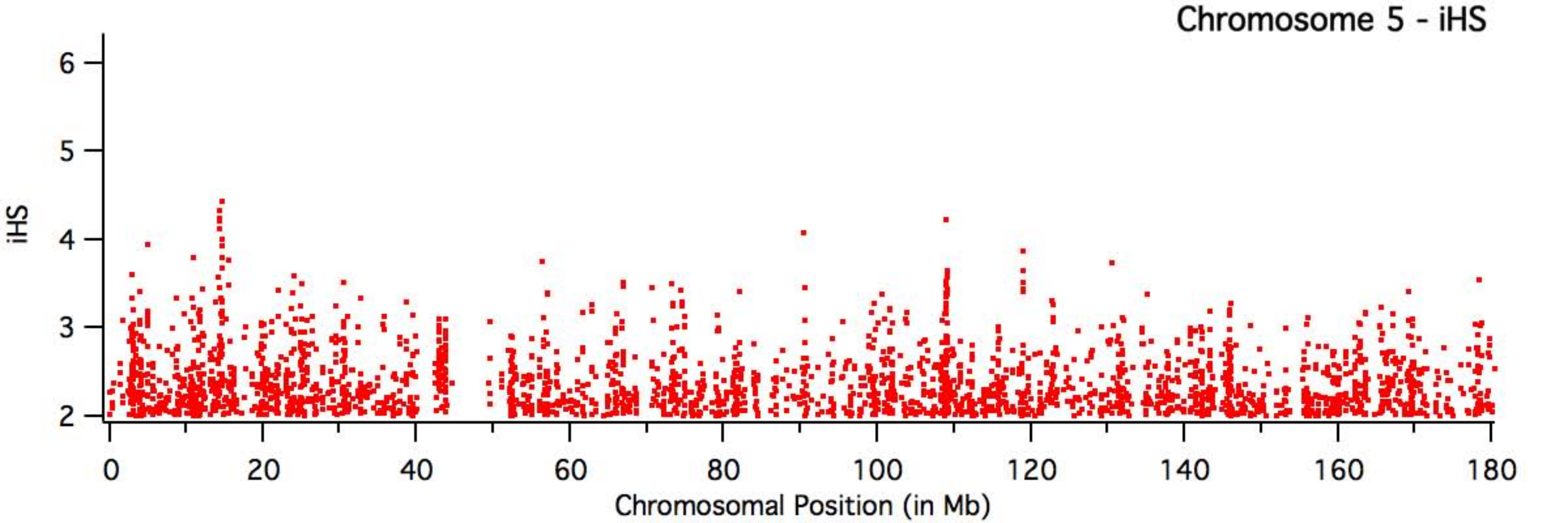
Chromosome 5 - iHS
iHS
6
5
4
3
2
0
20
40
60
80
100
120
140
160
180
Chromosomal Position (in Mb)

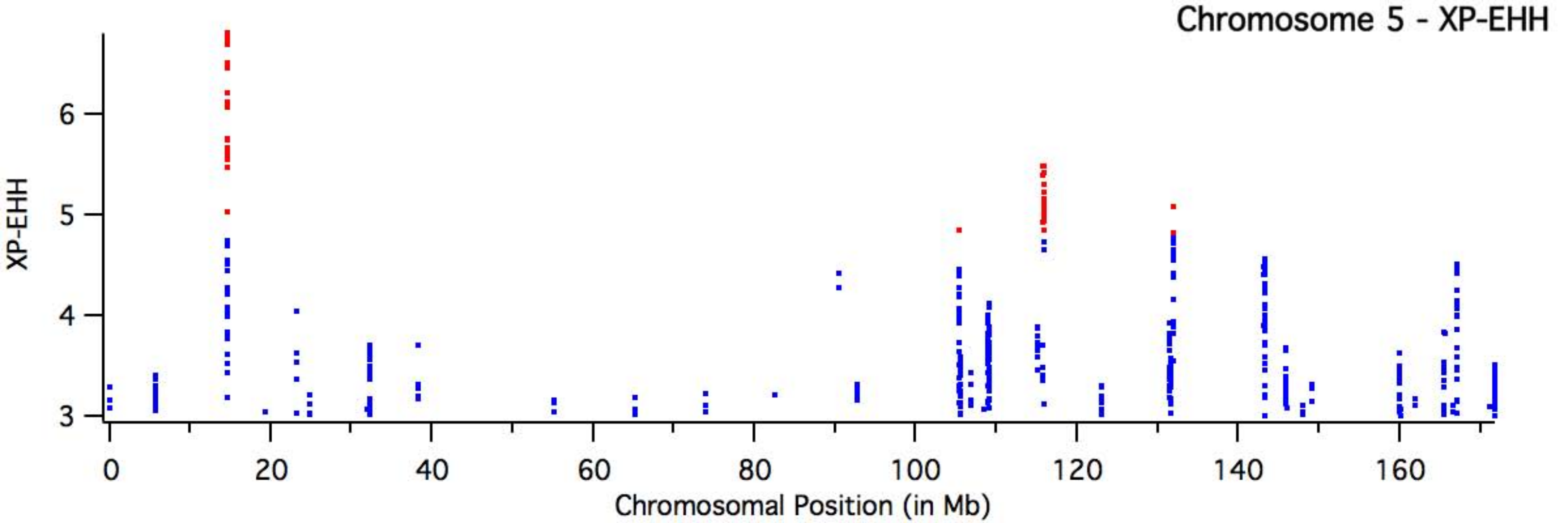
Chromosome 5 - XP-EHH
XP-EHH
3
4
5
6
0
20
40
60
80
100
120
140
160
Chromosomal Position (in Mb)

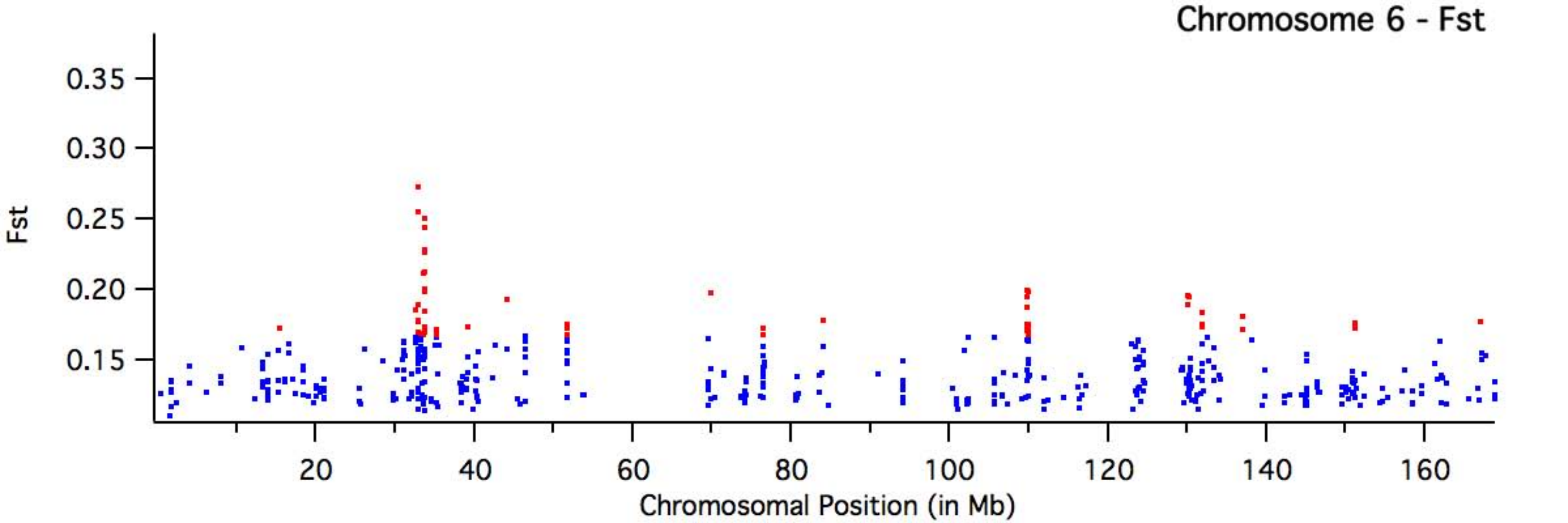
Chromosome 6 - Fst
Fst
0.35
0.30
0.25
0.20
0.15
20
40
60
80
100
120
140
160
Chromosomal Position (in Mb)

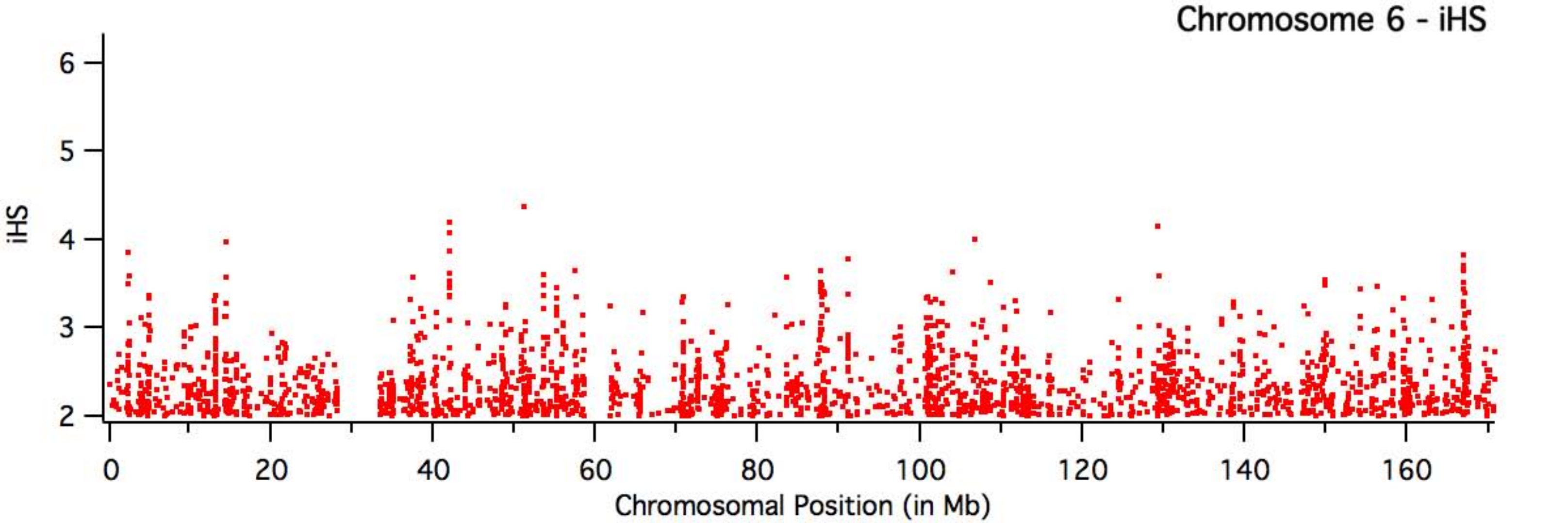

Chromosome 6 - iHS
iHS
6
5
4
3
2
0
20
40
60
80
100
120
140
160
Chromosomal Position (in Mb)

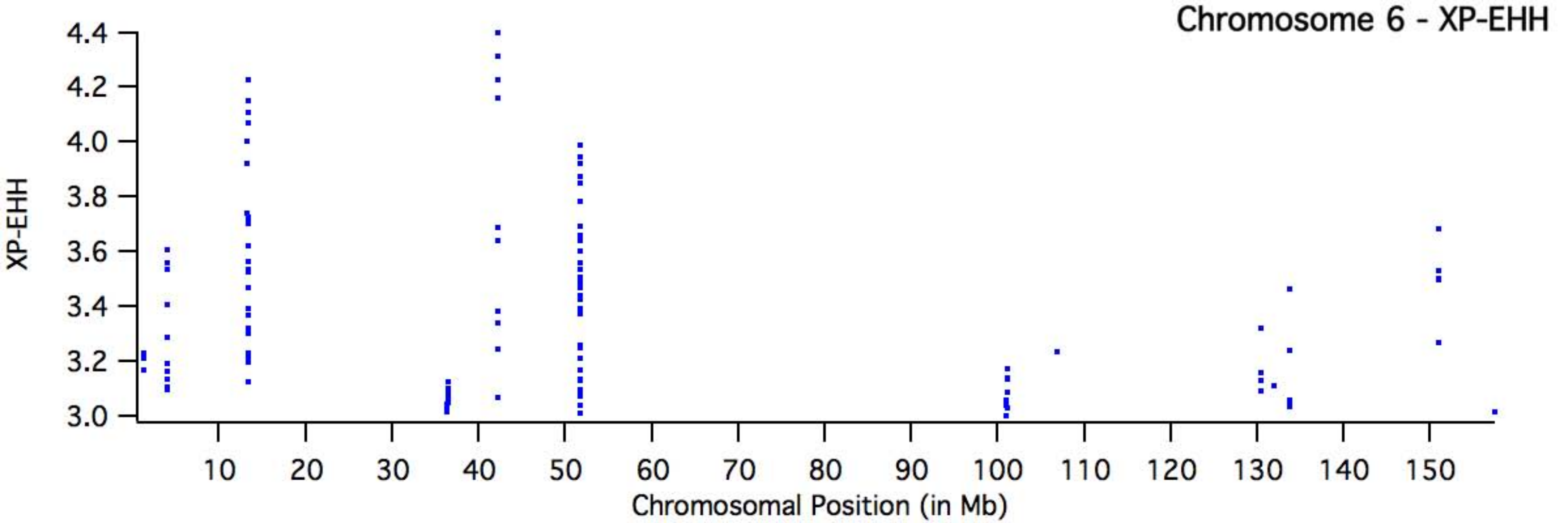
Chromosome 6 - XP-EHH
XP-EHH
4.4
4.2
4.0
3.8
3.6
3.4
3.2
3.0
10
20
30
40
50
60
70
80
90
100
110
120
130
140
150
Chromosomal Position (in Mb)

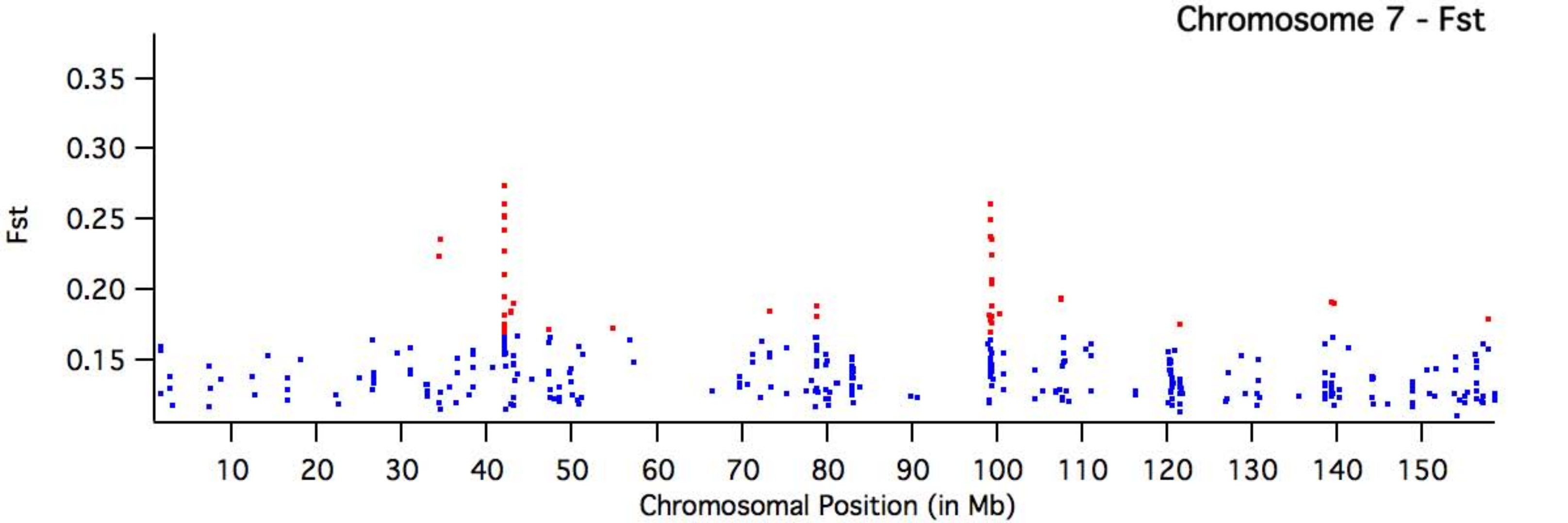

Chromosome 7 - Fst
Fst
0.35
0.30
0.25
0.20
0.15
10
20
30
40
50
60
70
80
90
100
110
120
130
140
150
Chromosomal Position (in Mb)

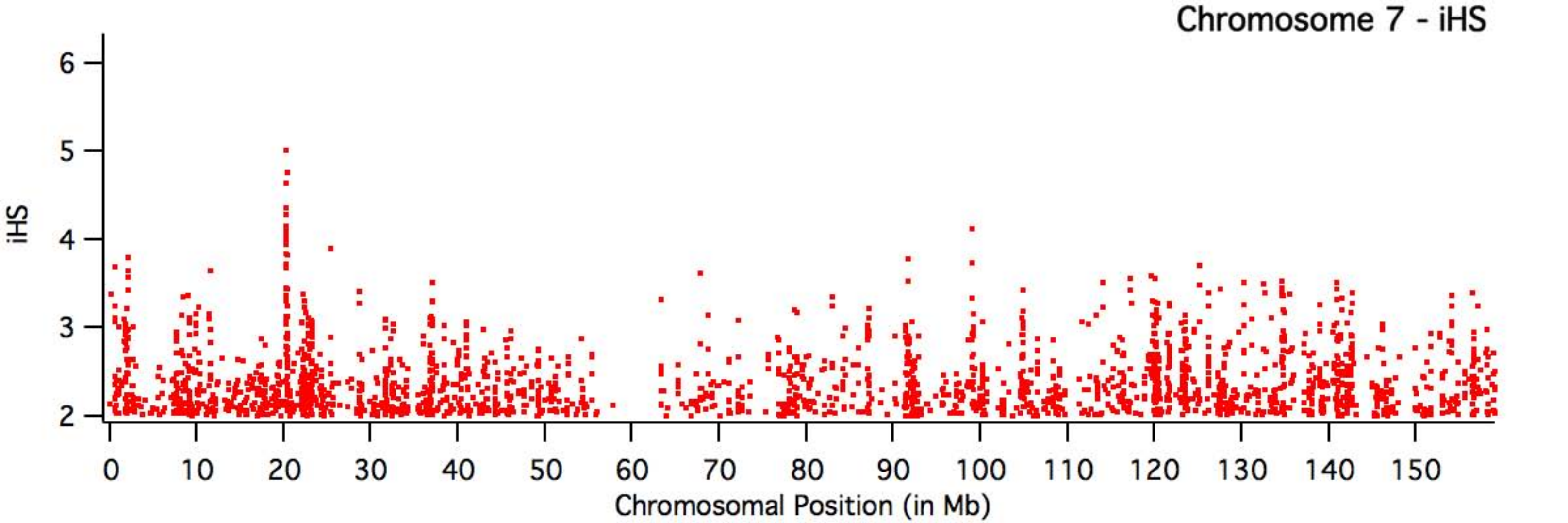

Chromosome 7 - iHS
iHS
6
5
4
3
2
0
10
20
30
40
50
60
70
80
90
100
110
120
130
140
150
Chromosomal Position (in Mb)

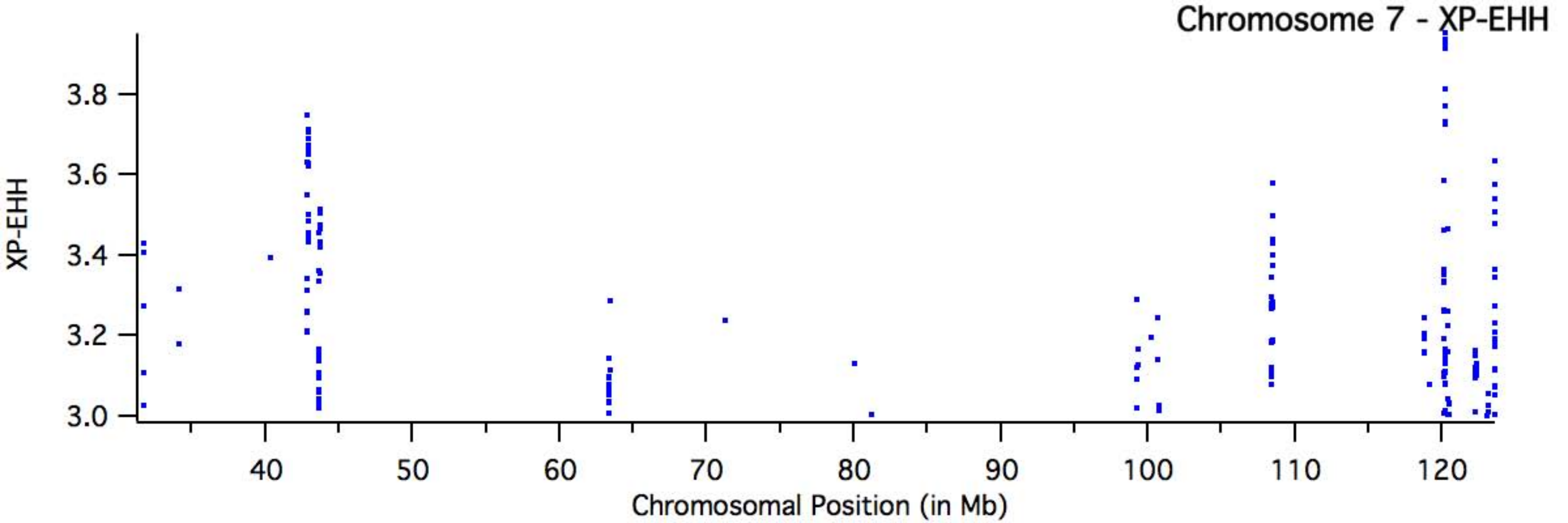
Chromosome 7 - XP-EHH
XP-EHH
3.8
3.6
3.4
3.2
3.0
40
50
60
70
80
90
100
110
120
Chromosomal Position (in Mb)

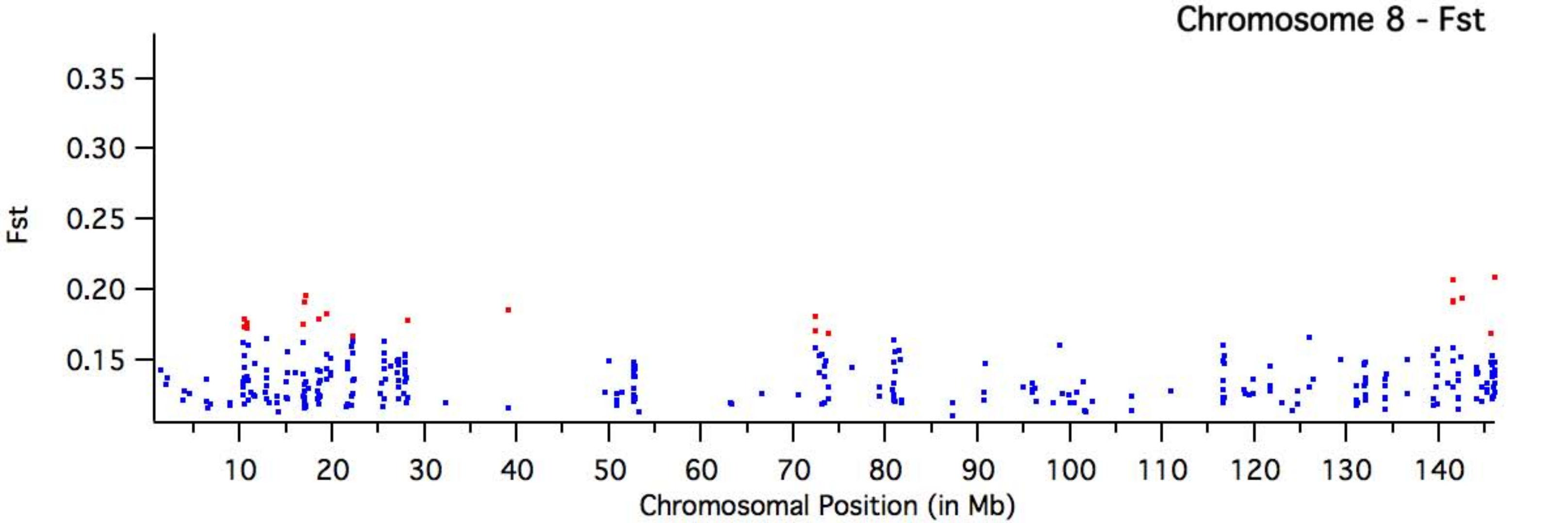
Chromosome 8 - Fst
Fst
0.35
0.30
0.25
0.20
0.15
10
20
30
40
50
60
70
80
90
100
110
120
130
140
Chromosomal Position (in Mb)

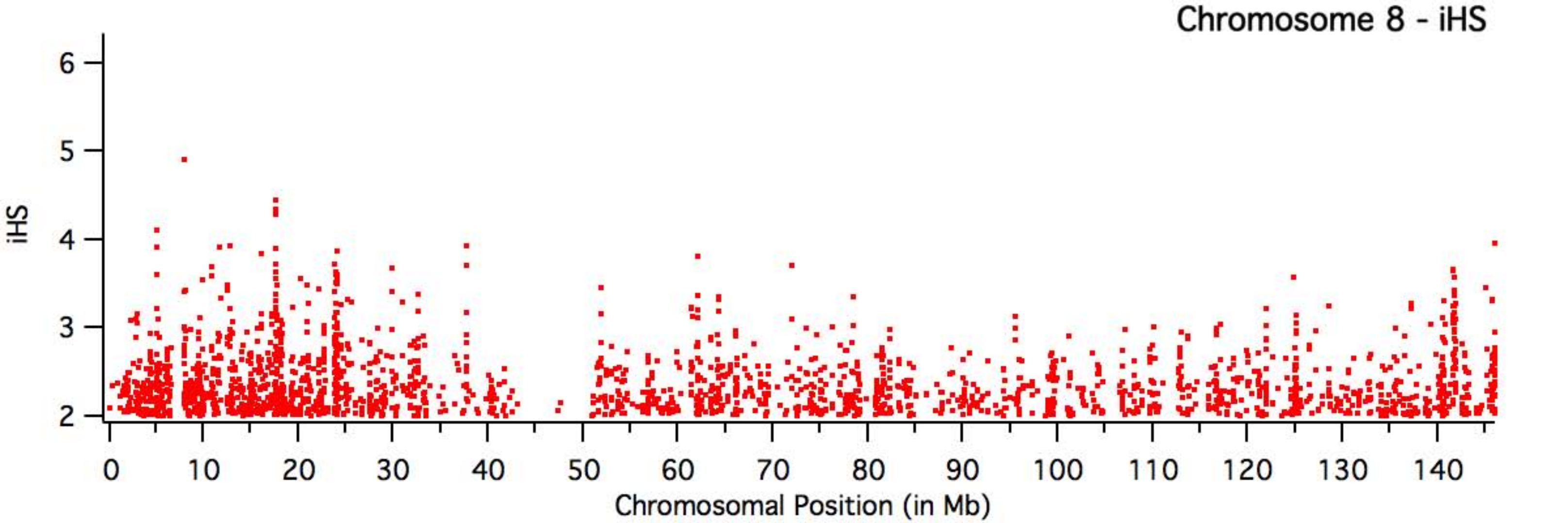
Chromosome 8 - iHS
iHS
6
5
4
3
2
0
10
20
30
40
50
60
70
80
90
100
110
120
130
140
Chromosomal Position (in Mb)

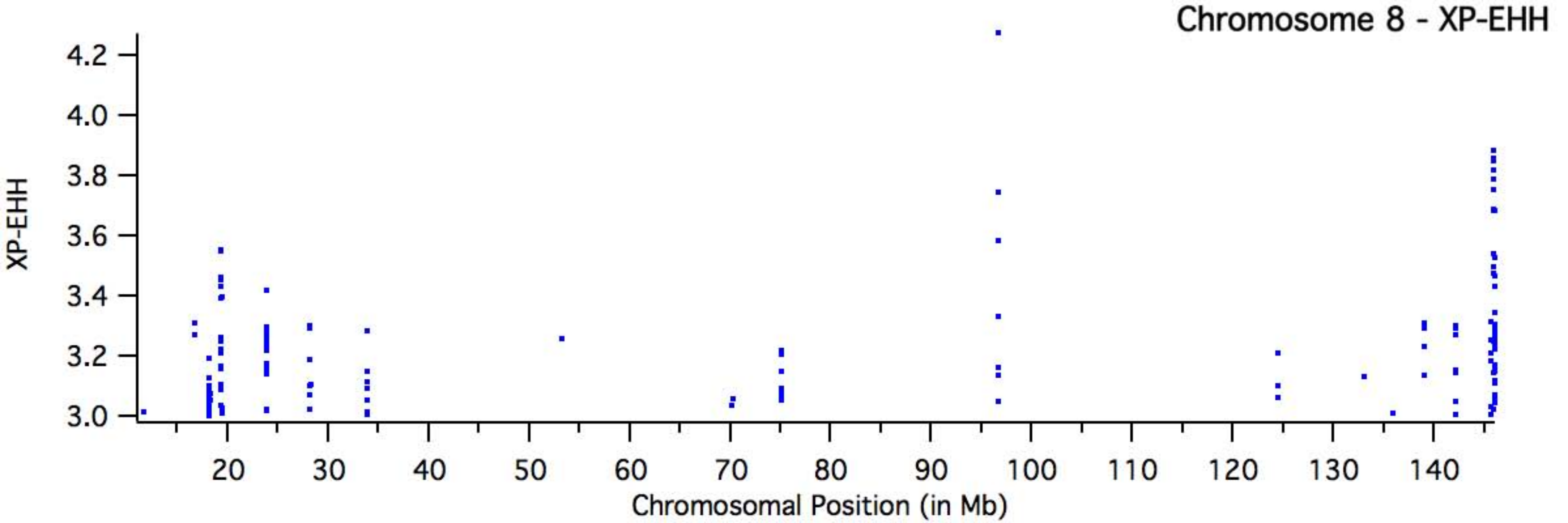

Chromosome 8 - XP-EHH
XP-EHH
4.2
4.0
3.8
3.6
3.4
3.2
3.0
20
30
40
50
60
70
80
90
100
110
120
130
140
Chromosomal Position (in Mb)

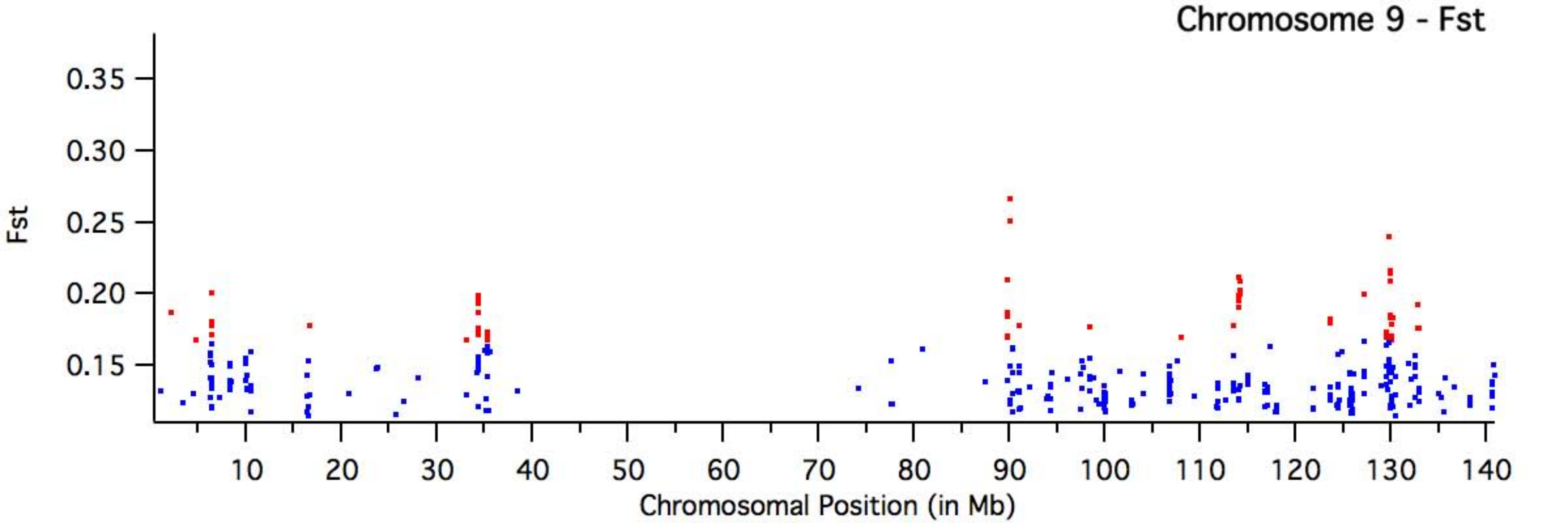
Chromosome 9 - Fst
Fst
0.35
0.30
0.25
0.20
0.15
10
20
30
40
50
60
70
80
90
100
110
120
130
140
Chromosomal Position (in Mb)

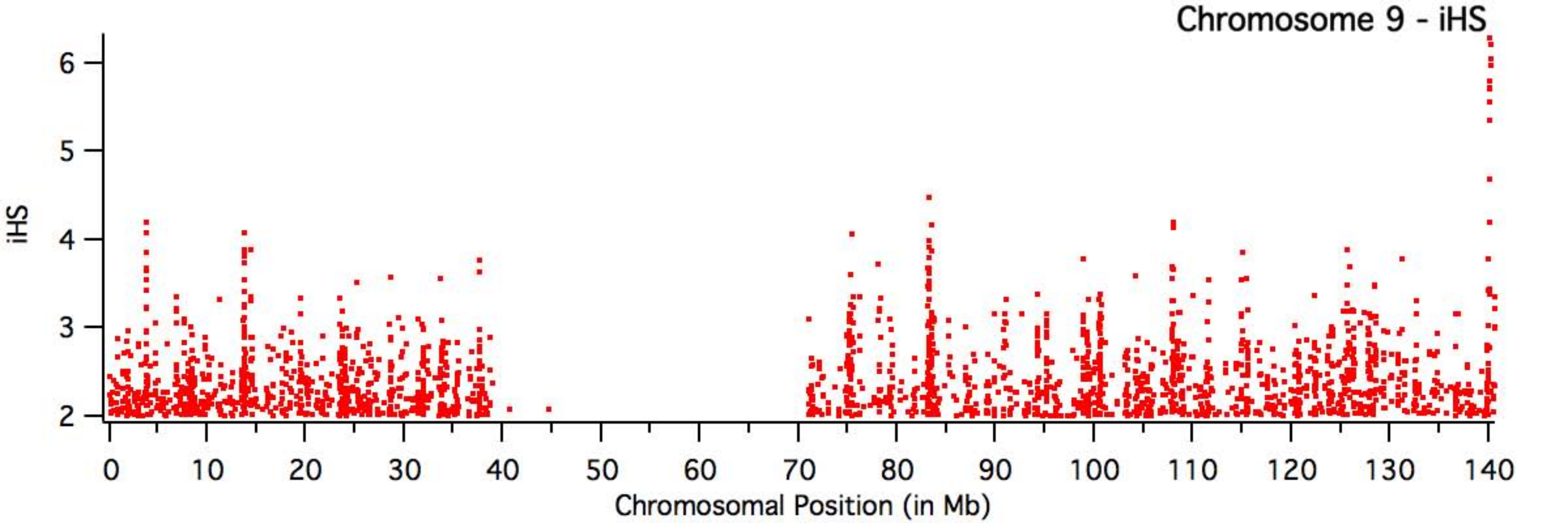
Chromosome 9 - iHS
iHS
2
3
4
5
6
0
10
20
30
40
50
60
70
80
90
100
110
120
130
140
Chromosomal Position (in Mb)

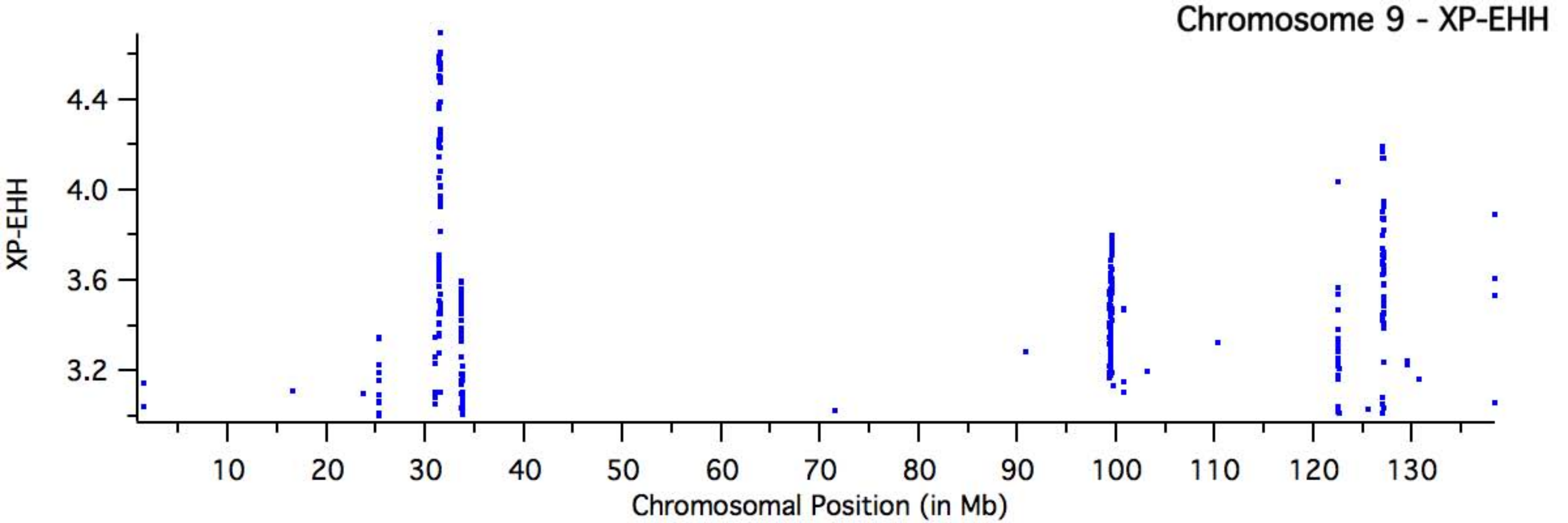
Chromosome 9 - XP-EHH
XP-EHH
4.4
4.0
3.6
3.2
10
20
30
40
50
60
70
80
90
100
110
120
130
Chromosomal Position (in Mb)

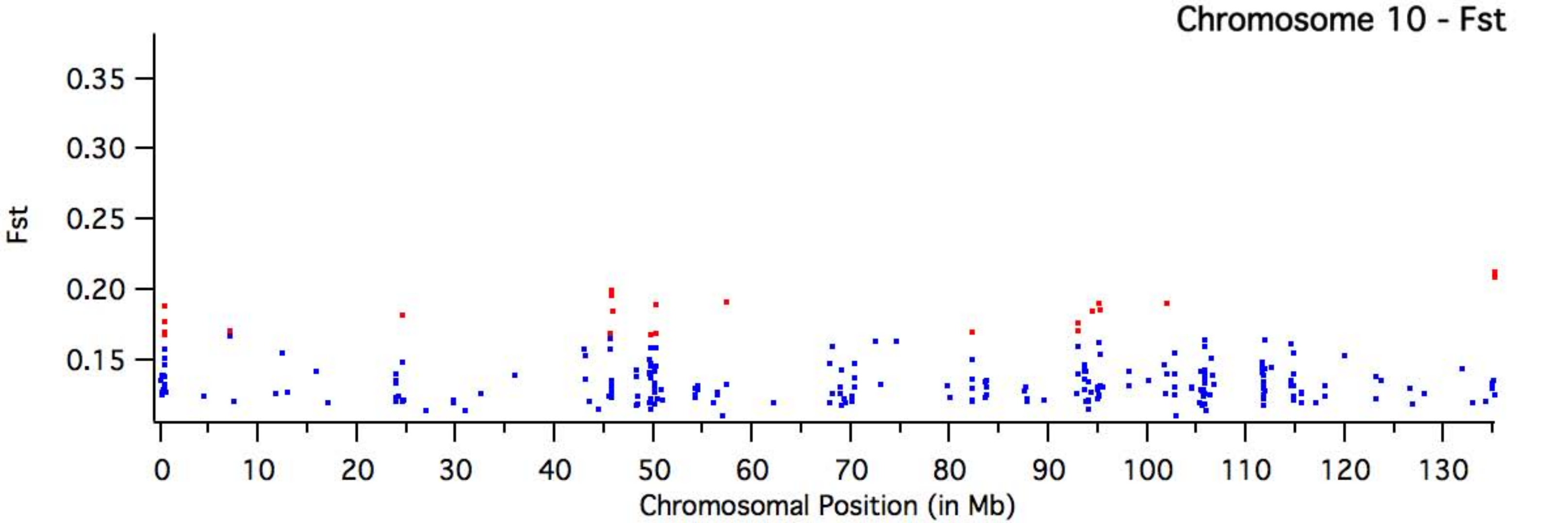

Chromosome 10 - Fst
Fst
0.35
0.30
0.25
0.20
0.15
0
10
20
30
40
50
60
70
80
90
100
110
120
130
Chromosomal Position (in Mb)

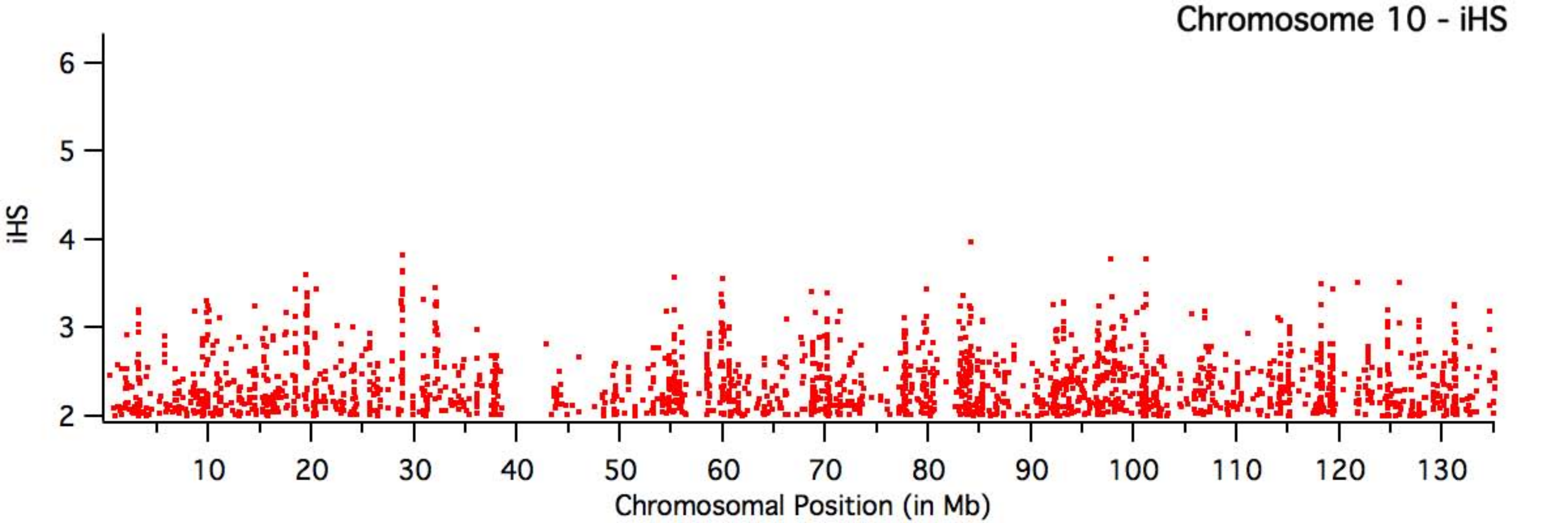
Chromosome 10 - iHS
iHS
6
5
4
3
2
10
20
30
40
50
60
70
80
90
100
110
120
130
Chromosomal Position (in Mb)

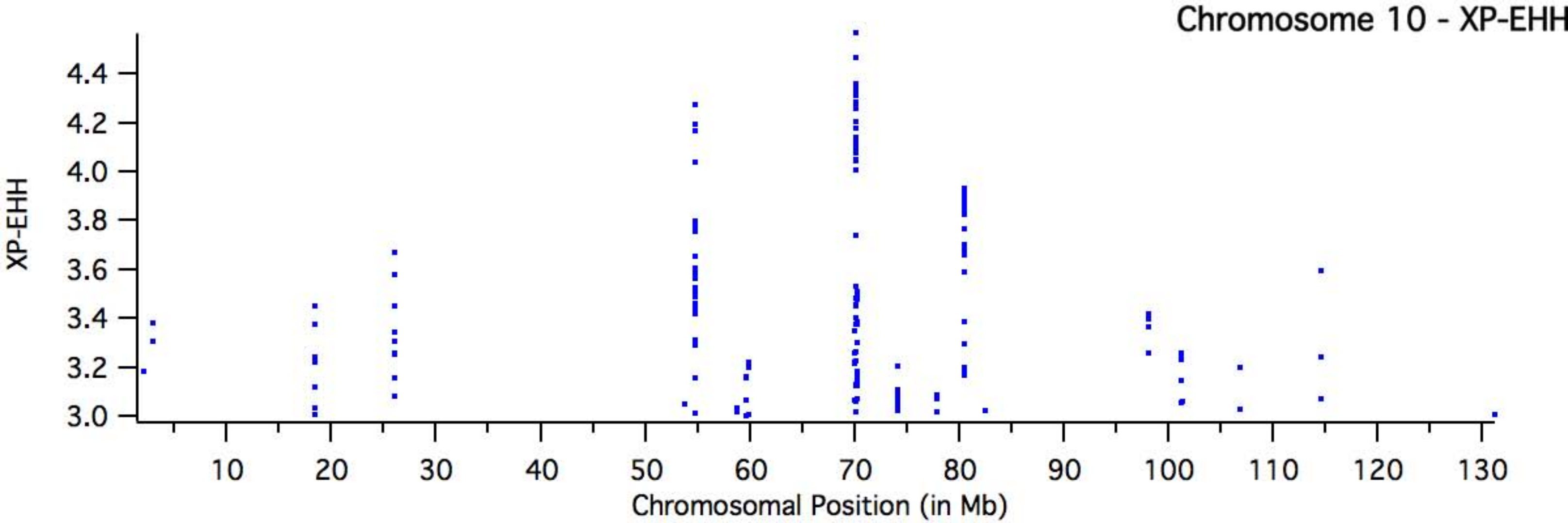
Chromosome 10 - XP-EHH
XP-EHH
4.4
4.2
4.0
3.8
3.6
3.4
3.2
3.0
10
20
30
40
50
60
70
80
90
100
110
120
130
Chromosomal Position (in Mb)

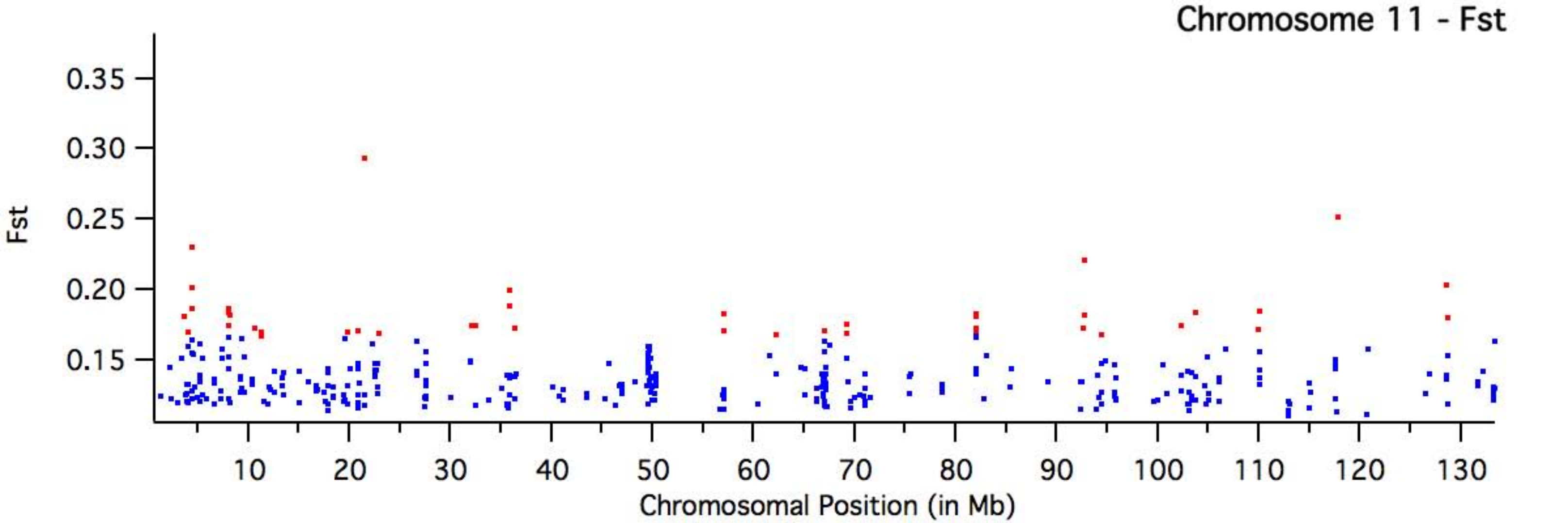
Chromosome 11 - Fst
Fst
0.35
0.30
0.25
0.20
0.15
10
20
30
40
50
60
70
80
90
100
110
120
130
Chromosomal Position (in Mb)

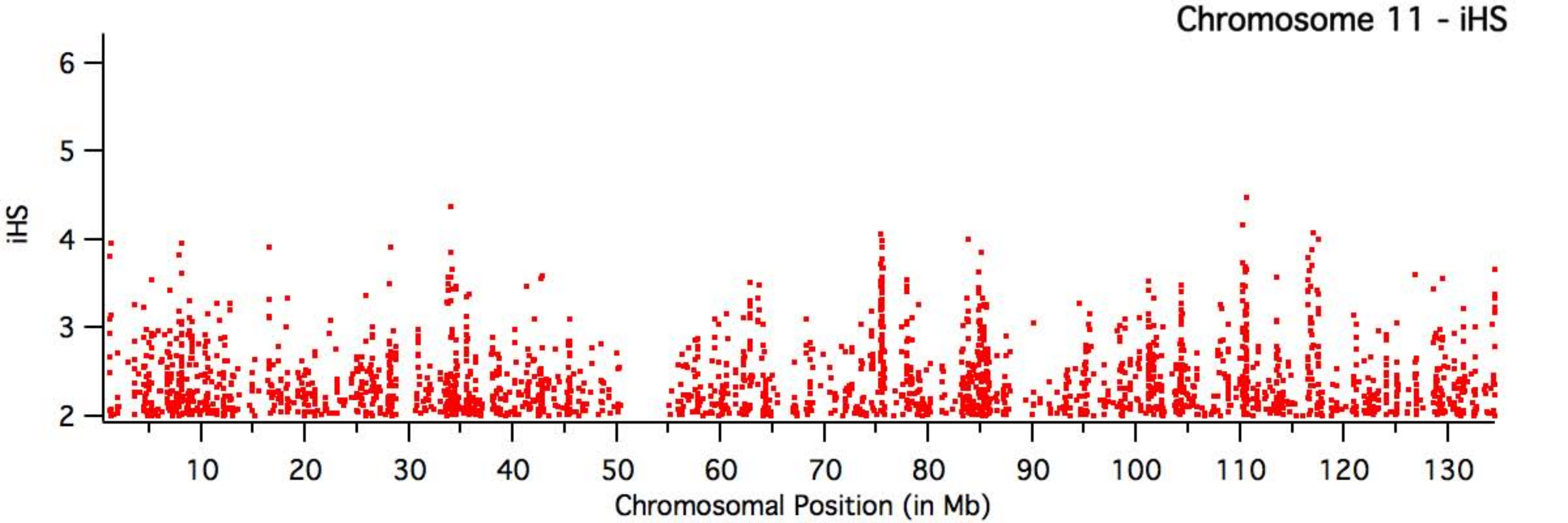

Chromosome 11 - iHS
iHS
6
5
4
3
2
10
20
30
40
50
60
70
80
90
100
110
120
130
Chromosomal Position (in Mb)

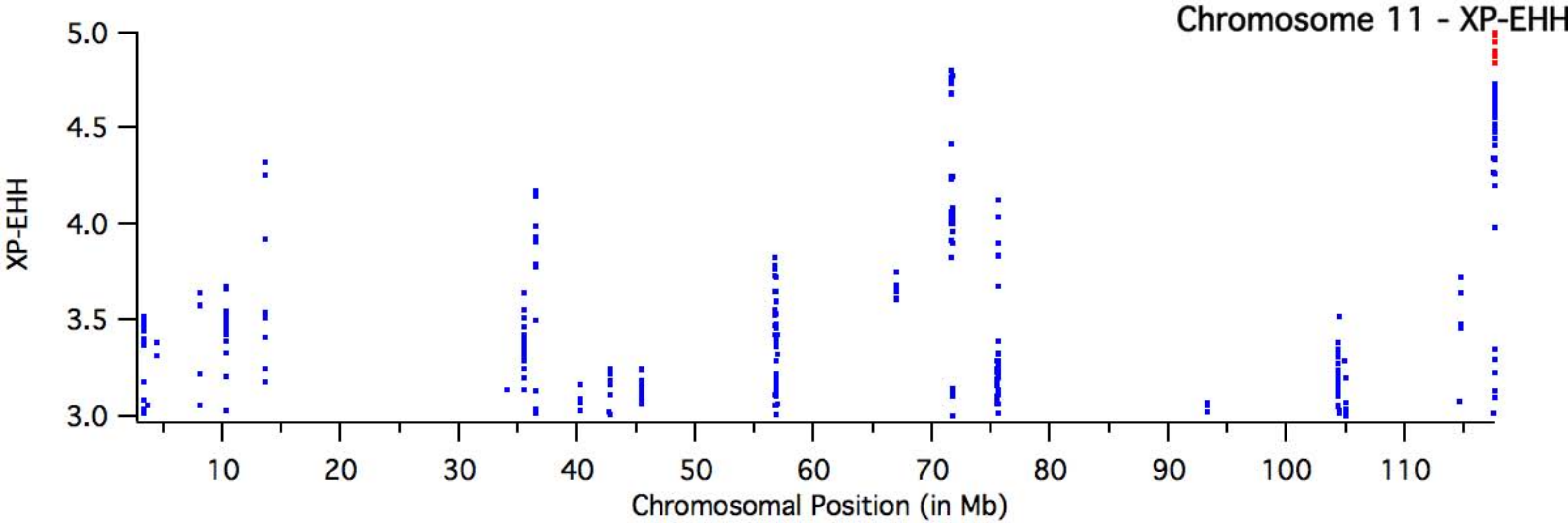
Chromosome 11 - XP-EHH
XP-EHH
5.0
4.5
4.0
3.5
3.0
10
20
30
40
50
60
70
80
90
100
110
Chromosomal Position (in Mb)

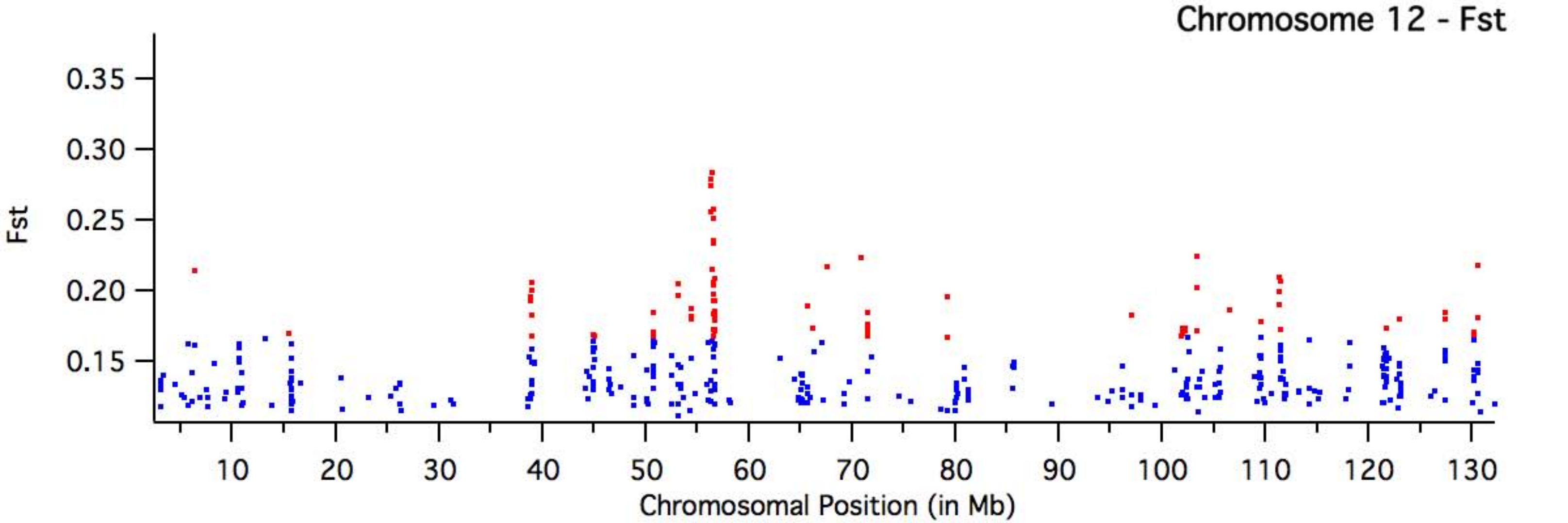
Chromosome 12 - Fst
Fst
0.35
0.30
0.25
0.20
0.15
10
20
30
40
50
60
70
80
90
100
110
120
130
Chromosomal Position (in Mb)

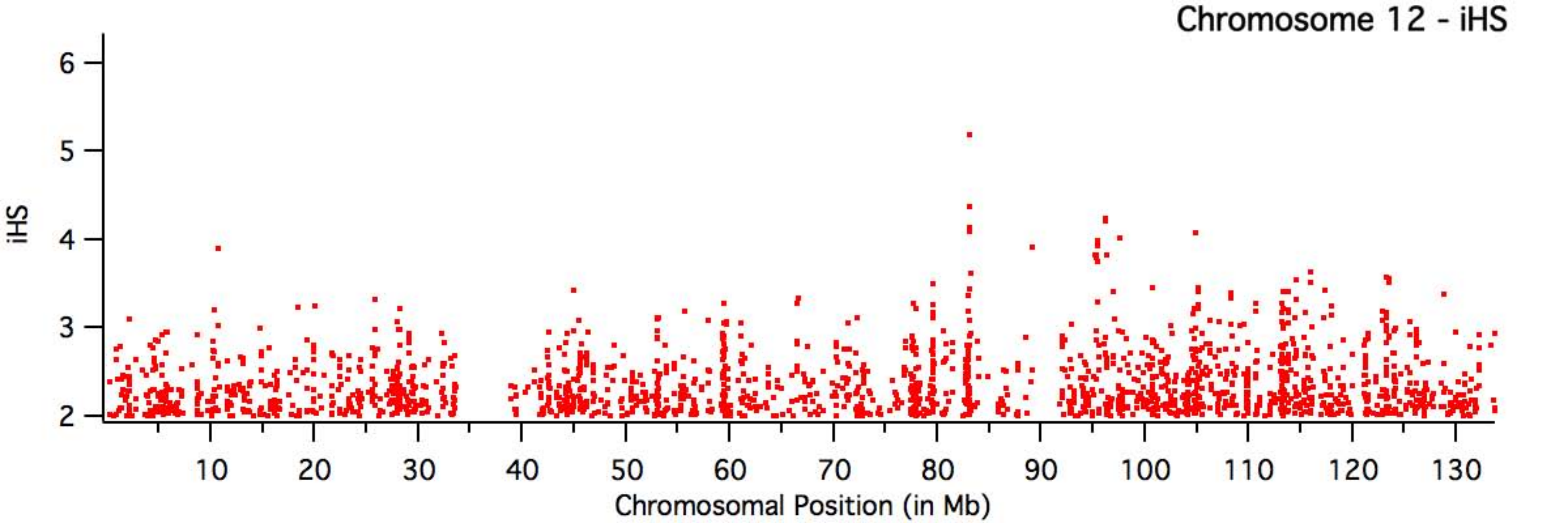

Chromosome 12 - iHS
iHS
6
5
4
3
2
10
20
30
40
50
60
70
80
90
100
110
120
130
Chromosomal Position (in Mb)

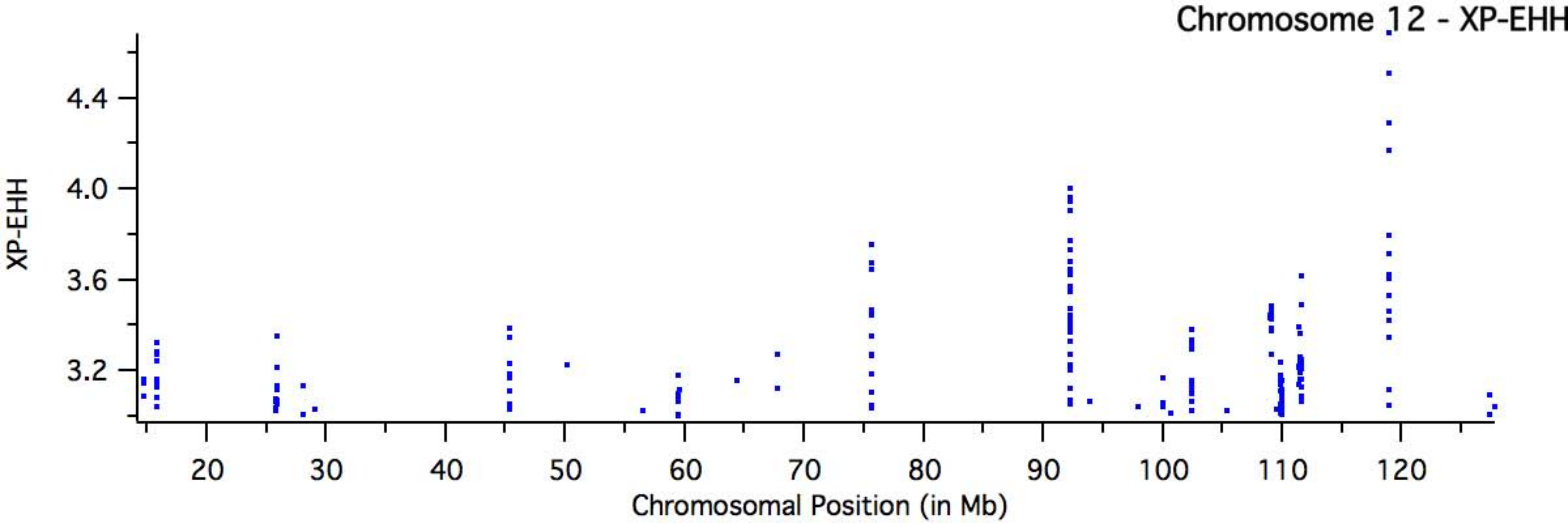
Chromosome 12 - XP-EHH
XP-EHH
4.4
4.0
3.6
3.2
20
30
40
50
60
70
80
90
100
110
120
Chromosomal Position (in Mb)

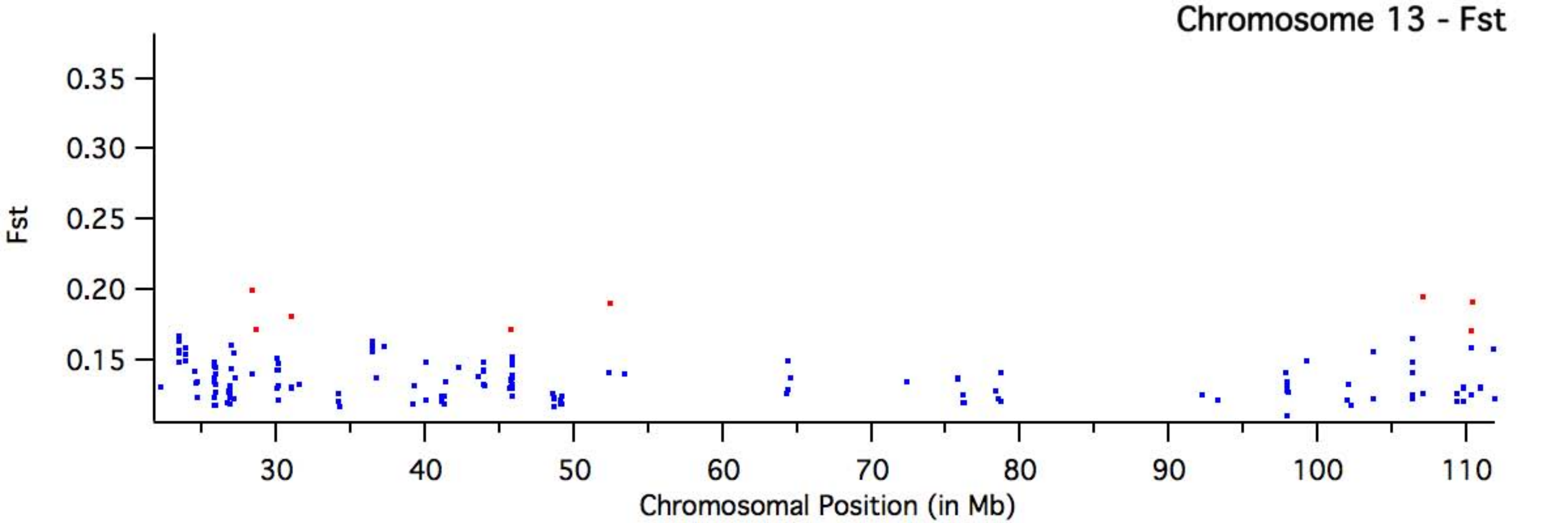
Chromosome 13 - Fst
Fst
0.35
0.30
0.25
0.20
0.15
30
40
50
60
70
80
90
100
110
Chromosomal Position (in Mb)

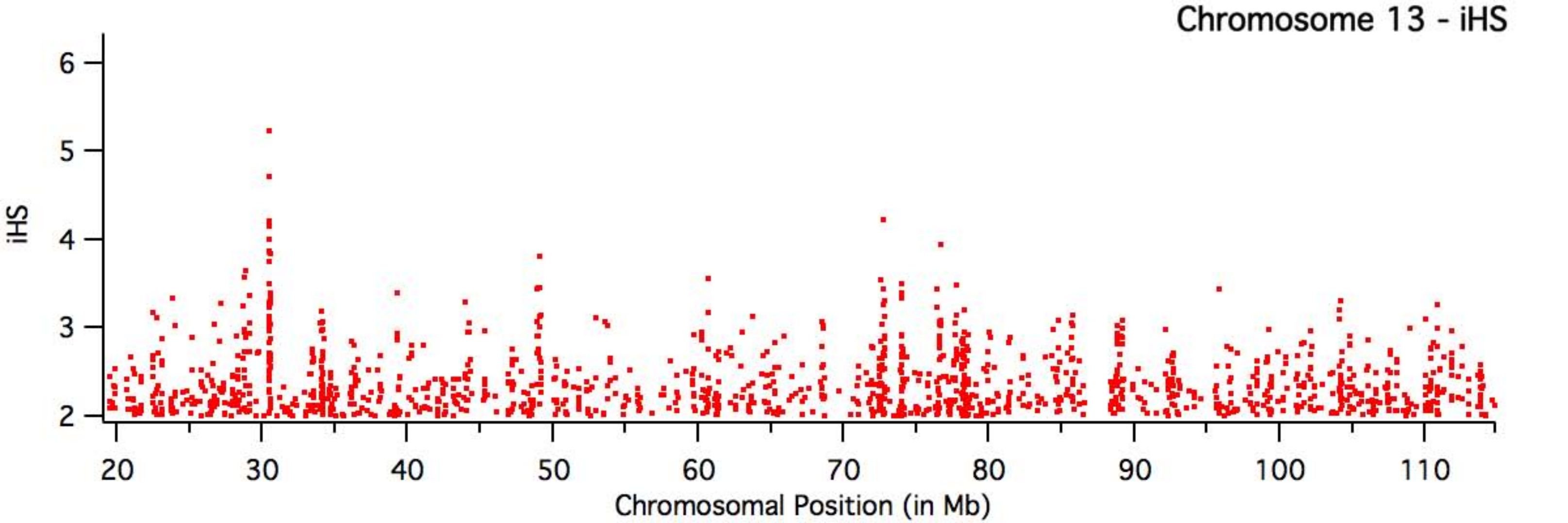
Chromosome 13 - iHS
iHS
6
5
4
3
2
20
30
40
50
60
70
80
90
100
110
Chromosomal Position (in Mb)

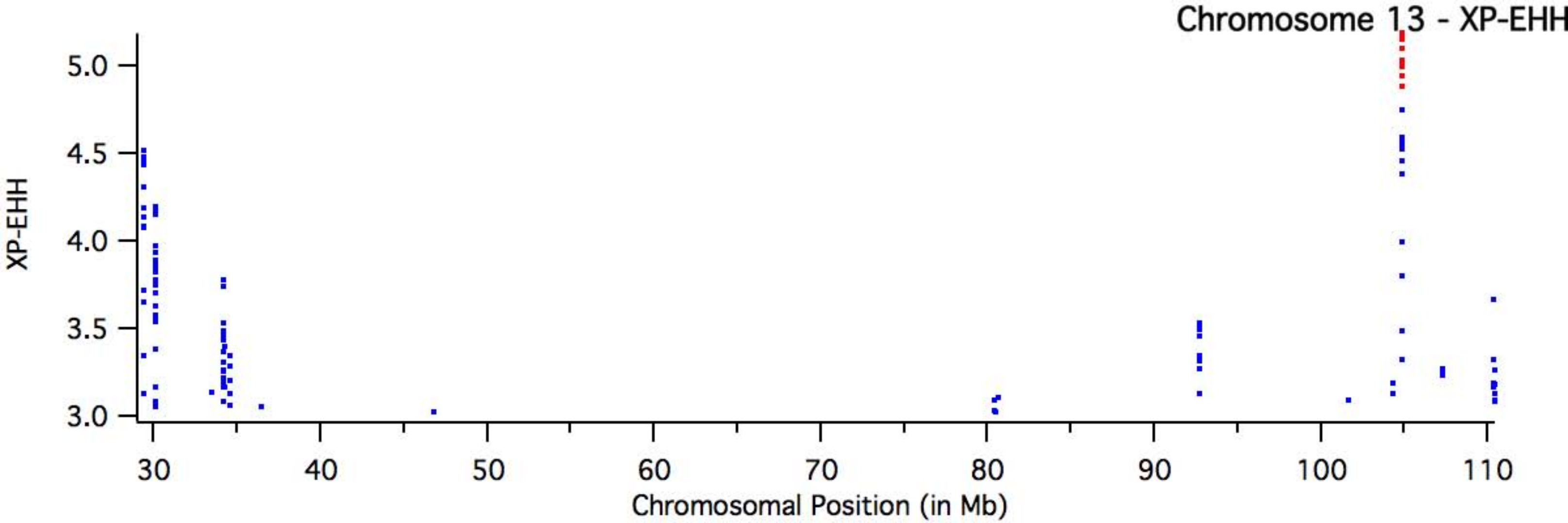
Chromosome 13 - XP-EHH
XP-EHH
5.0
4.5
4.0
3.5
3.0
30
40
50
60
70
80
90
100
110
Chromosomal Position (in Mb)

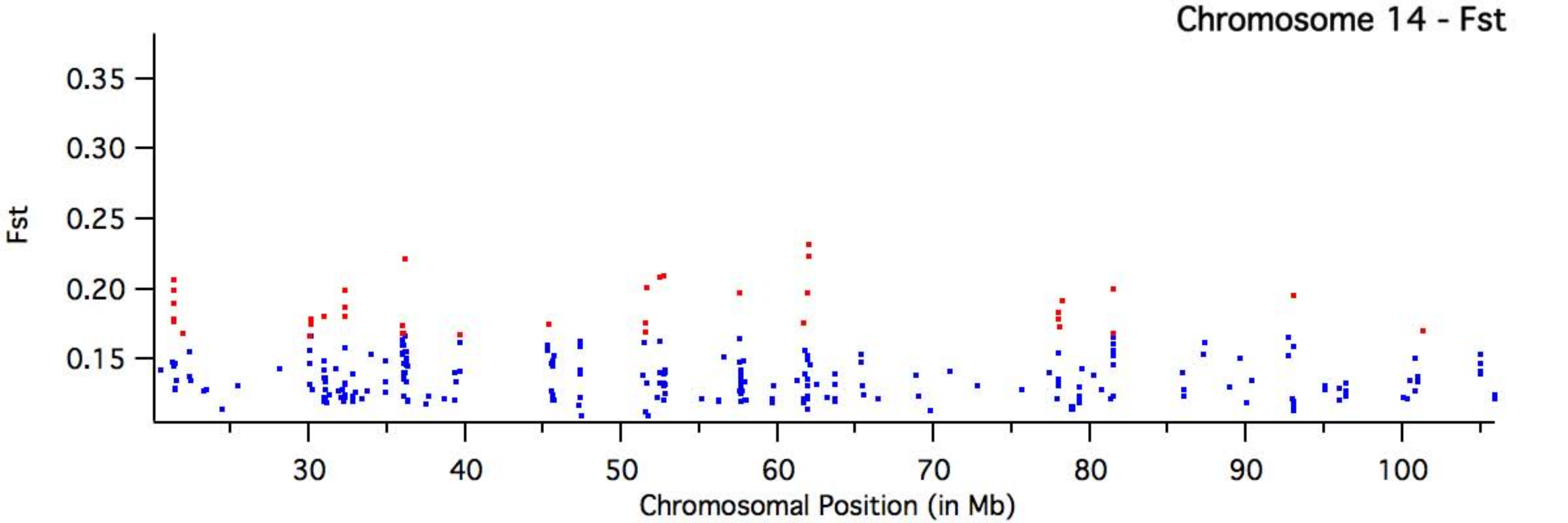
Chromosome 14 - Fst
Fst
0.35
0.30
0.25
0.20
0.15
30
40
50
60
70
80
90
100
Chromosomal Position (in Mb)

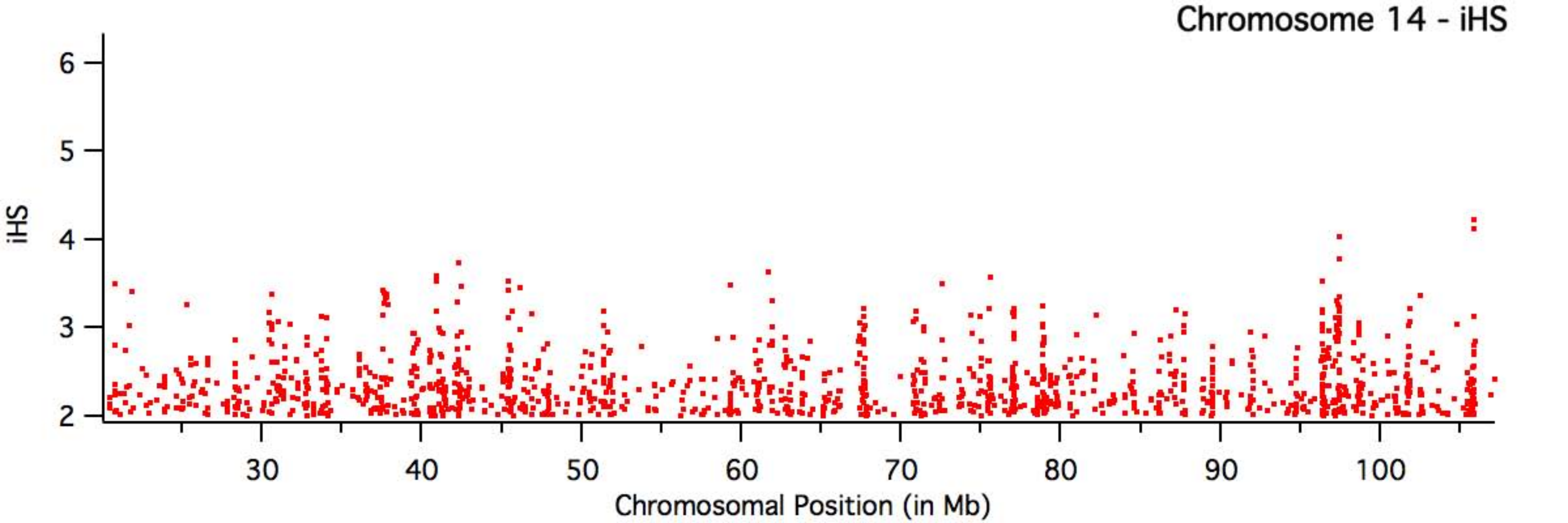
Chromosome 14 - iHS
iHS
6
5
4
3
2
30
40
50
60
70
80
90
100
Chromosomal Position (in Mb)

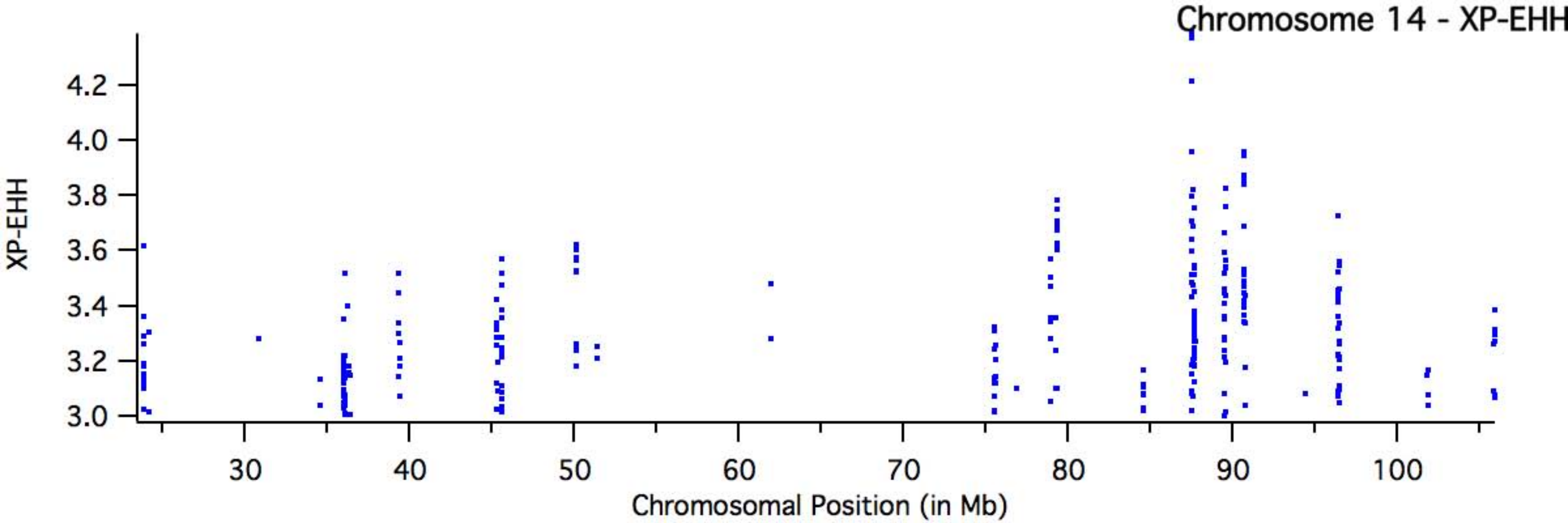

Chromosome 14 - XP-EHH
XP-EHH
4.2
4.0
3.8
3.6
3.4
3.2
3.0
30
40
50
60
70
80
90
100
Chromosomal Position (in Mb)

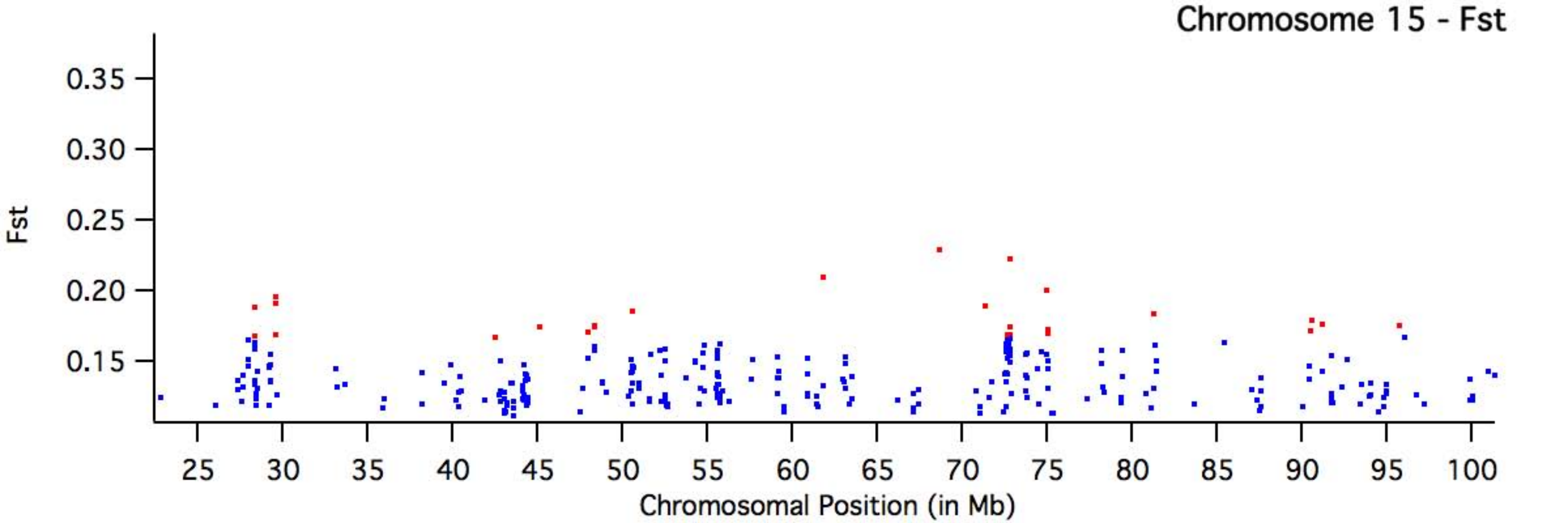

Chromosome 15 - Fst
Fst
0.35
0.30
0.25
0.20
0.15
25
30
35
40
45
50
55
60
65
70
75
80
85
90
95
100
Chromosomal Position (in Mb)

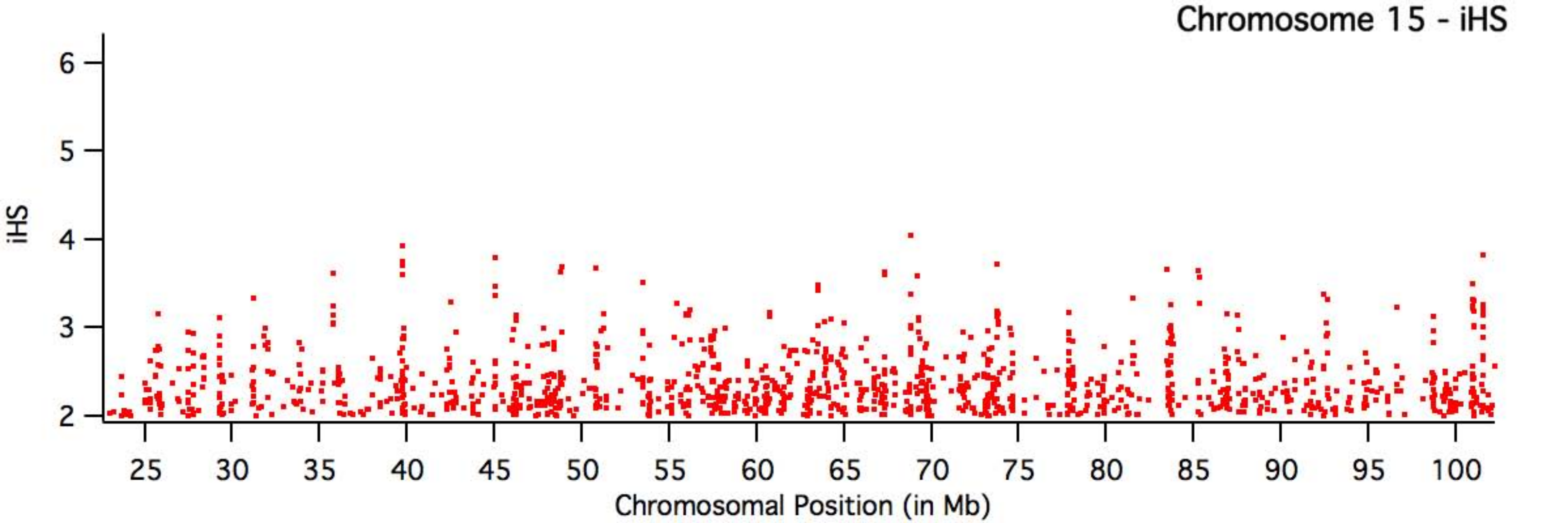
Chromosome 15 - iHS
iHS
6
5
4
3
2
25
30
35
40
45
50
55
60
65
70
75
80
85
90
95
100
Chromosomal Position (in Mb)

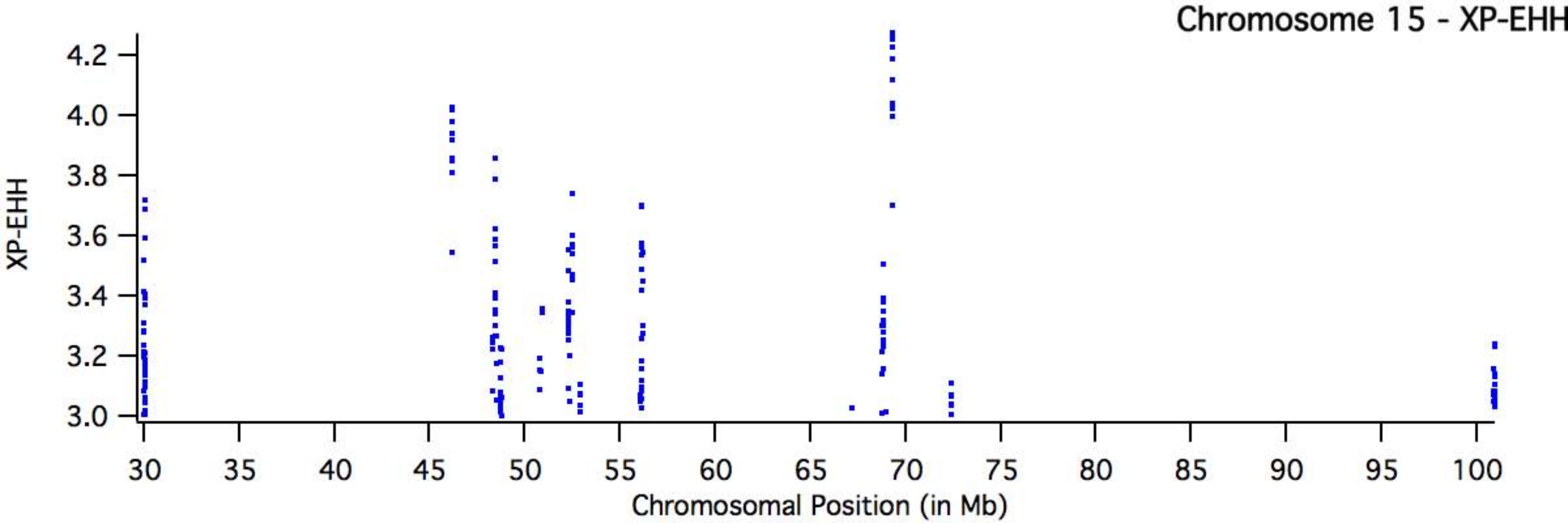

Chromosome 15 - XP-EHH
XP-EHH
4.2
4.0
3.8
3.6
3.4
3.2
3.0
30
35
40
45
50
55
60
65
70
75
80
85
90
95
100
Chromosomal Position (in Mb)

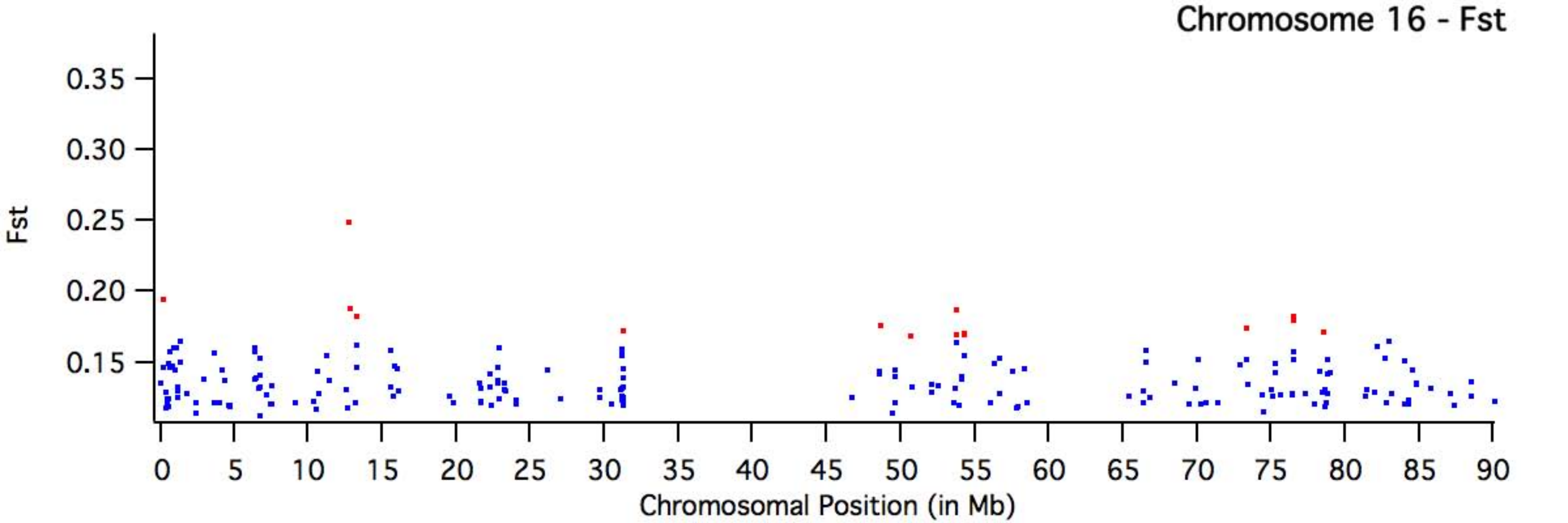
Chromosome 16 - Fst
Fst
0.35
0.30
0.25
0.20
0.15
0
5
10
15
20
25
30
35
40
45
50
55
60
65
70
75
80
85
90
Chromosomal Position (in Mb)

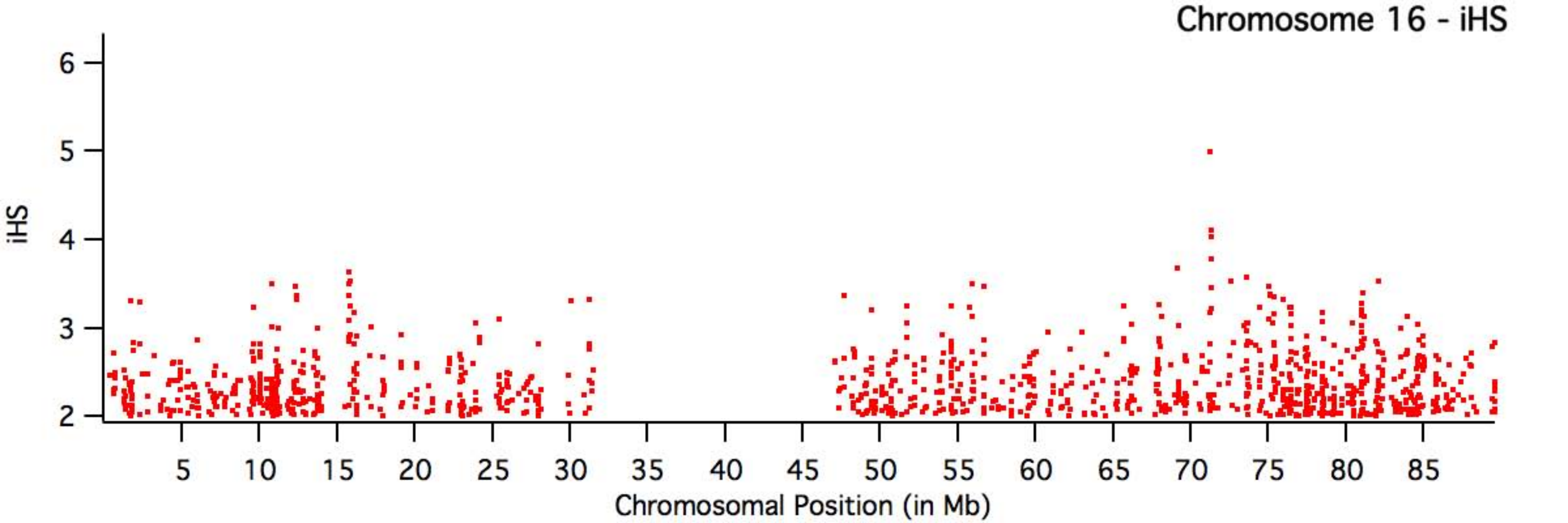

Chromosome 16 - iHS
iHS
6
5
4
3
2
5
10
15
20
25
30
35
40
45
50
55
60
65
70
75
80
85
Chromosomal Position (in Mb)

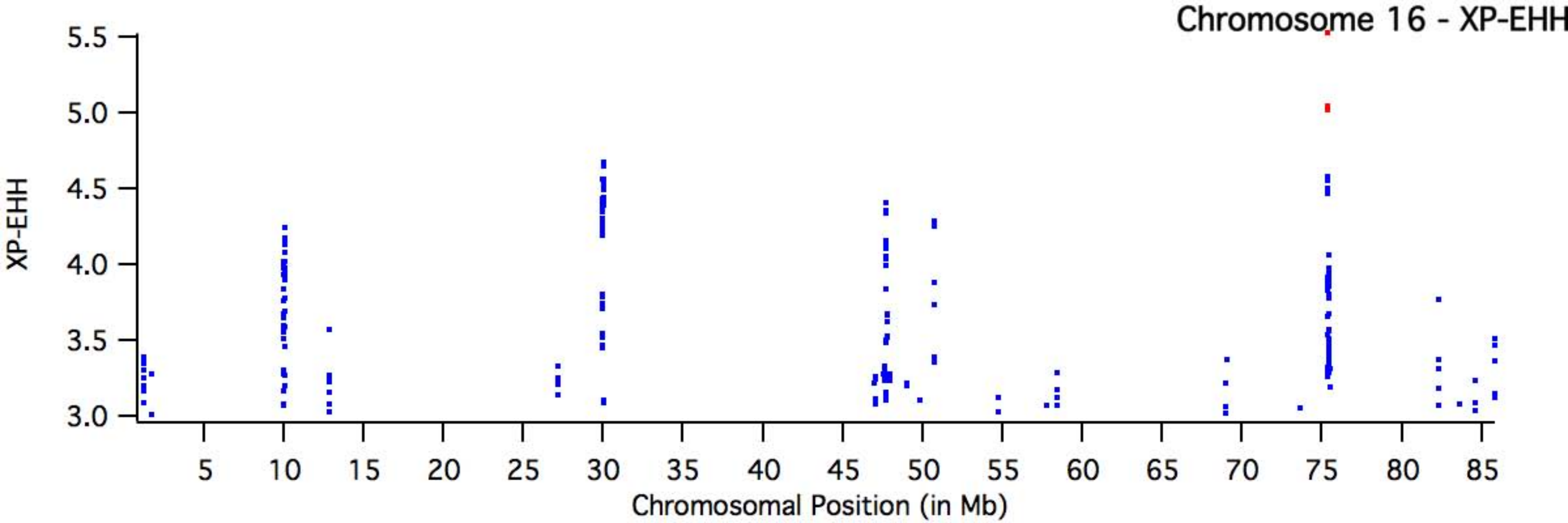
Chromosome 16 - XP-EHH
XP-EHH
5.5
5.0
4.5
4.0
3.5
3.0
5
10
15
20
25
30
35
40
45
50
55
60
65
70
75
80
85
Chromosomal Position (in Mb)

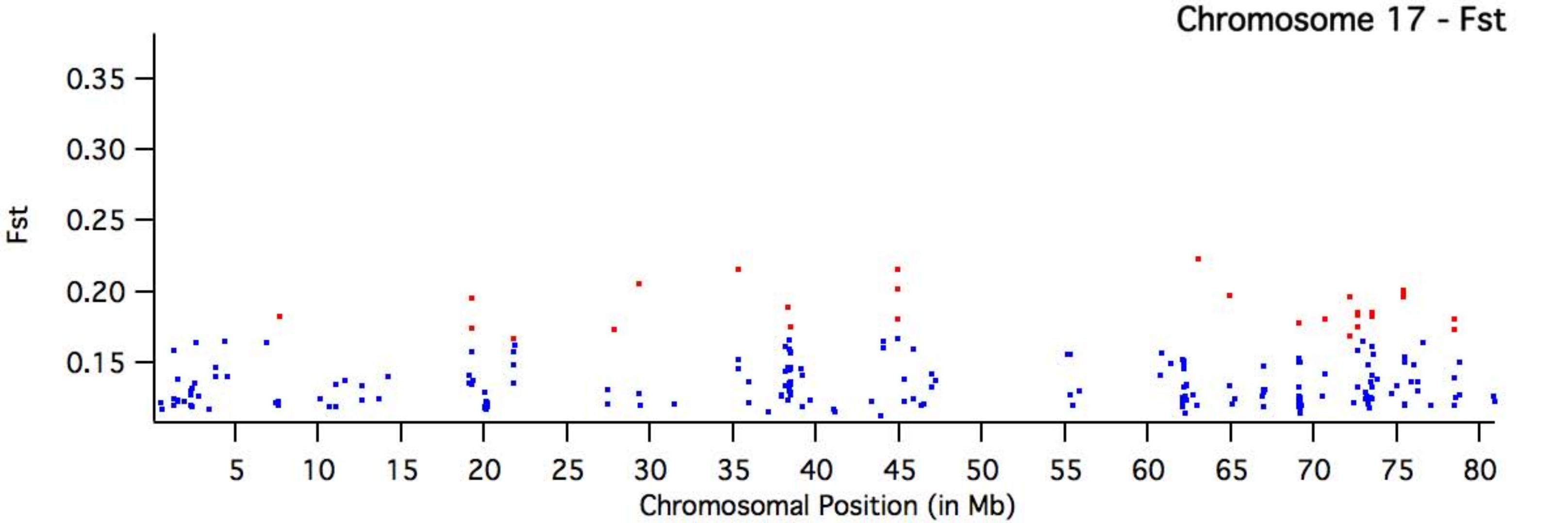

Chromosome 17 - Fst
Fst
0.35
0.30
0.25
0.20
0.15
5
10
15
20
25
30
35
40
45
50
55
60
65
70
75
80
Chromosomal Position (in Mb)

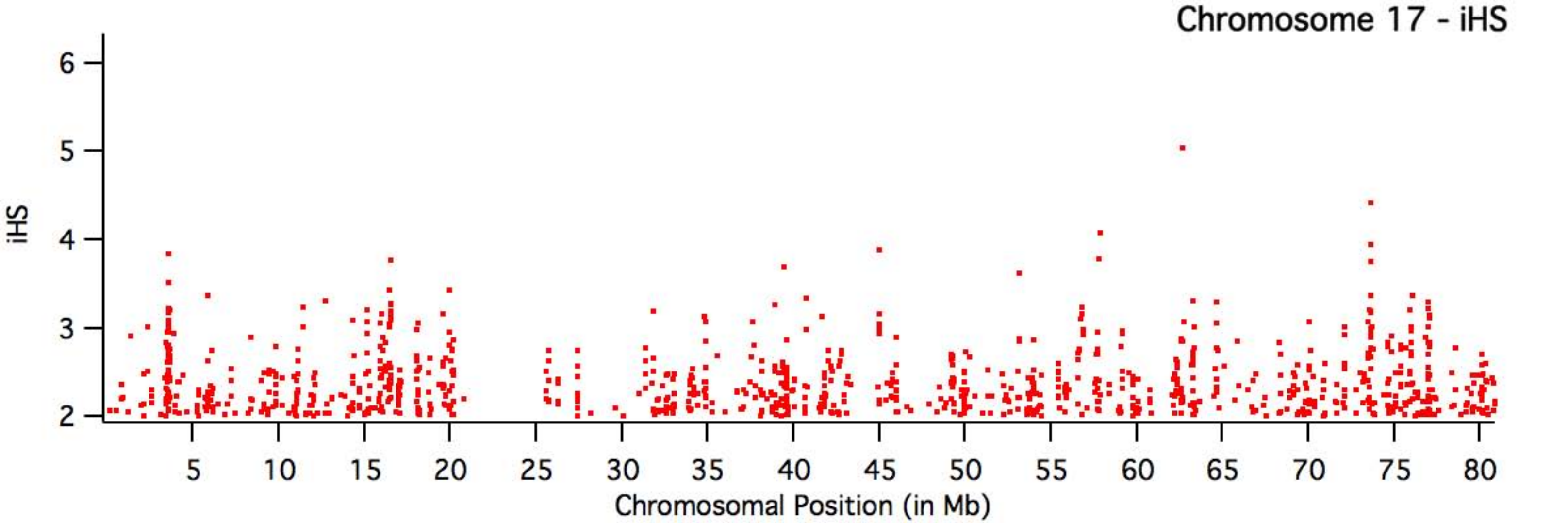

Chromosome 17 - iHS
iHS
2
3
4
5
6
5
10
15
20
25
30
35
40
45
50
55
60
65
70
75
80
Chromosomal Position (in Mb)

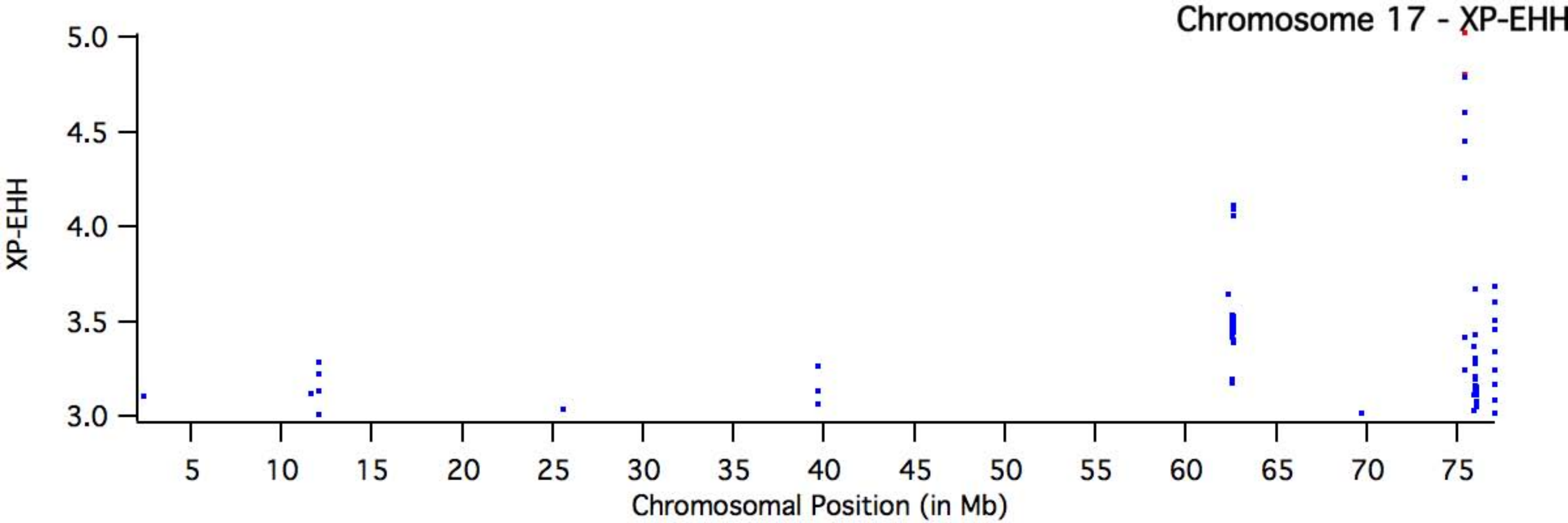
Chromosome 17 - XP-EHH
XP-EHH
5.0
4.5
4.0
3.5
3.0
5
10
15
20
25
30
35
40
45
50
55
60
65
70
75
Chromosomal Position (in Mb)

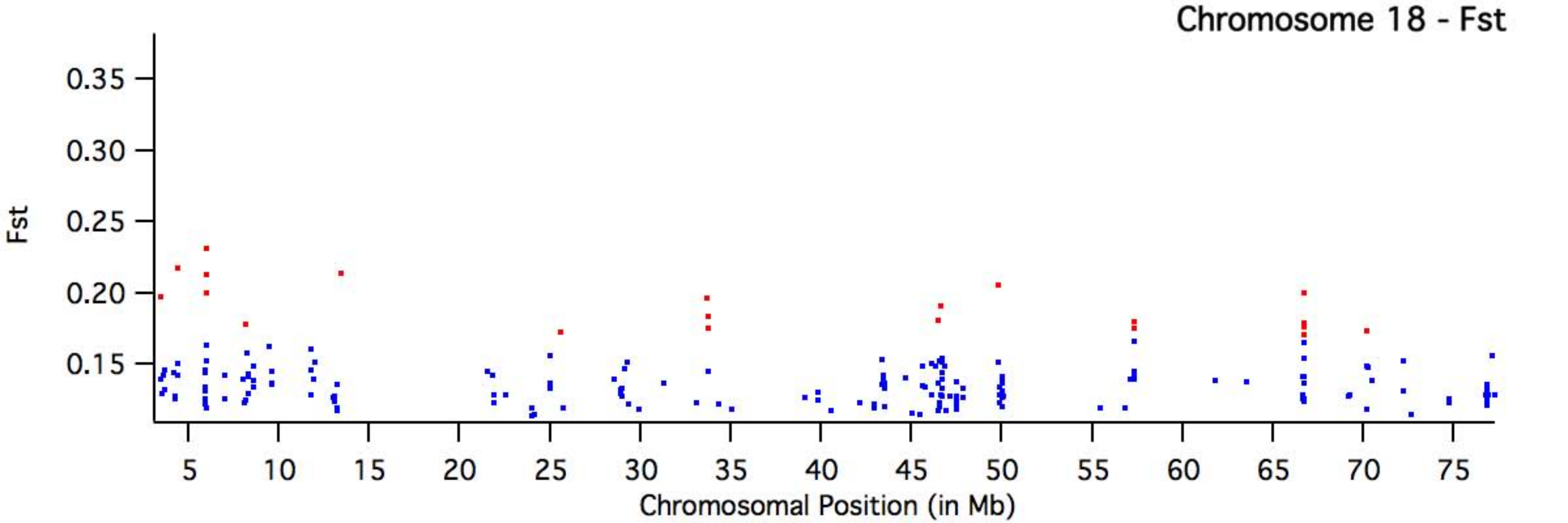
Chromosome 18 - Fst
Fst
0.35
0.30
0.25
0.20
0.15
5
10
15
20
25
30
35
40
45
50
55
60
65
70
75
Chromosomal Position (in Mb)

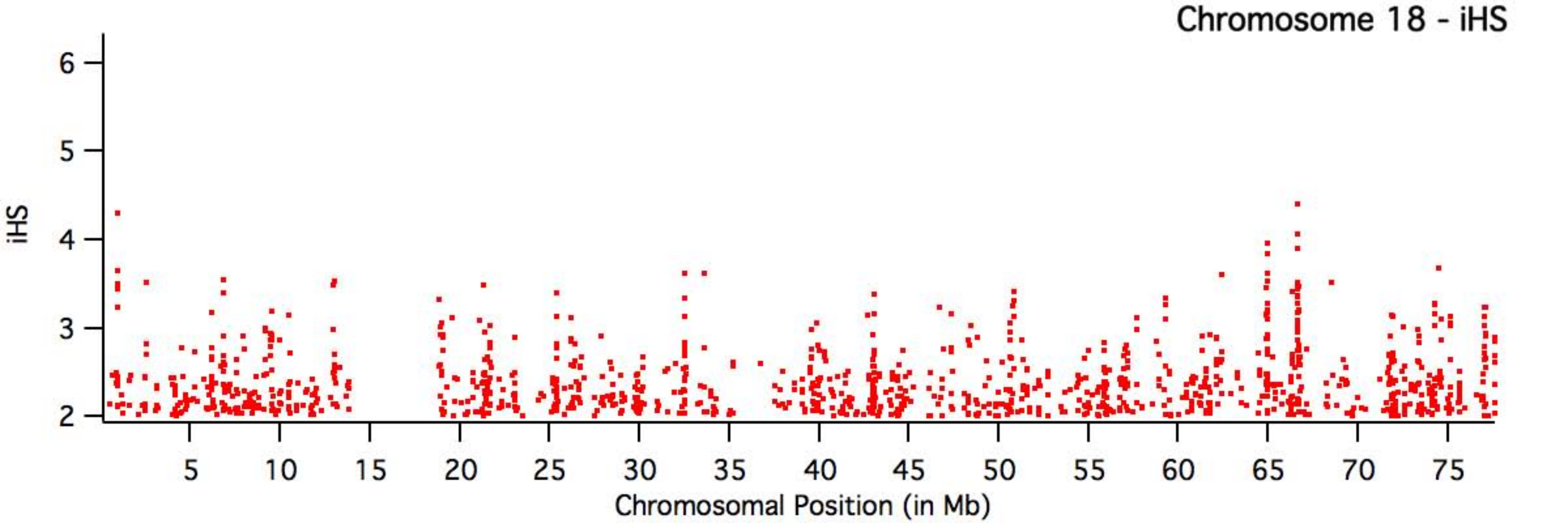

Chromosome 18 - iHS
iHS
6
5
4
3
2
5
10
15
20
25
30
35
40
45
50
55
60
65
70
75
Chromosomal Position (in Mb)

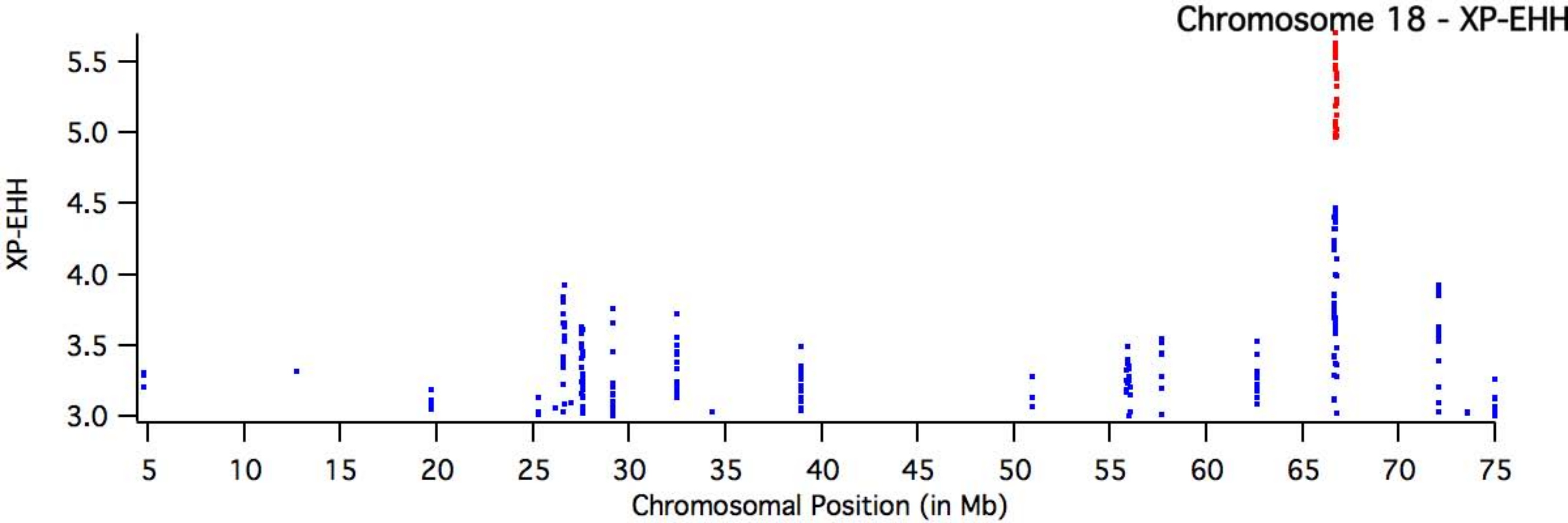
Chromosome 18 - XP-EHH
XP-EHH
5.5
5.0
4.5
4.0
3.5
3.0
5
10
15
20
25
30
35
40
45
50
55
60
65
70
75
Chromosomal Position (in Mb)

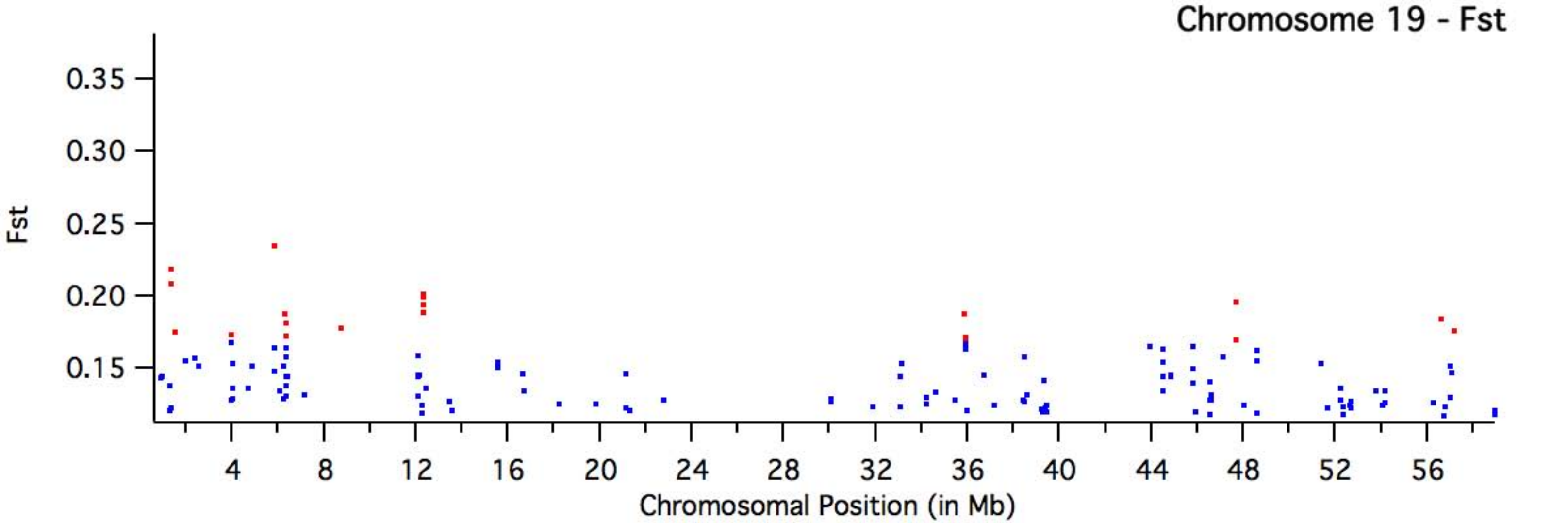
Chromosome 19 - Fst
Fst
0.35
0.30
0.25
0.20
0.15
4
8
12
16
20
24
28
32
36
40
44
48
52
56
Chromosomal Position (in Mb)

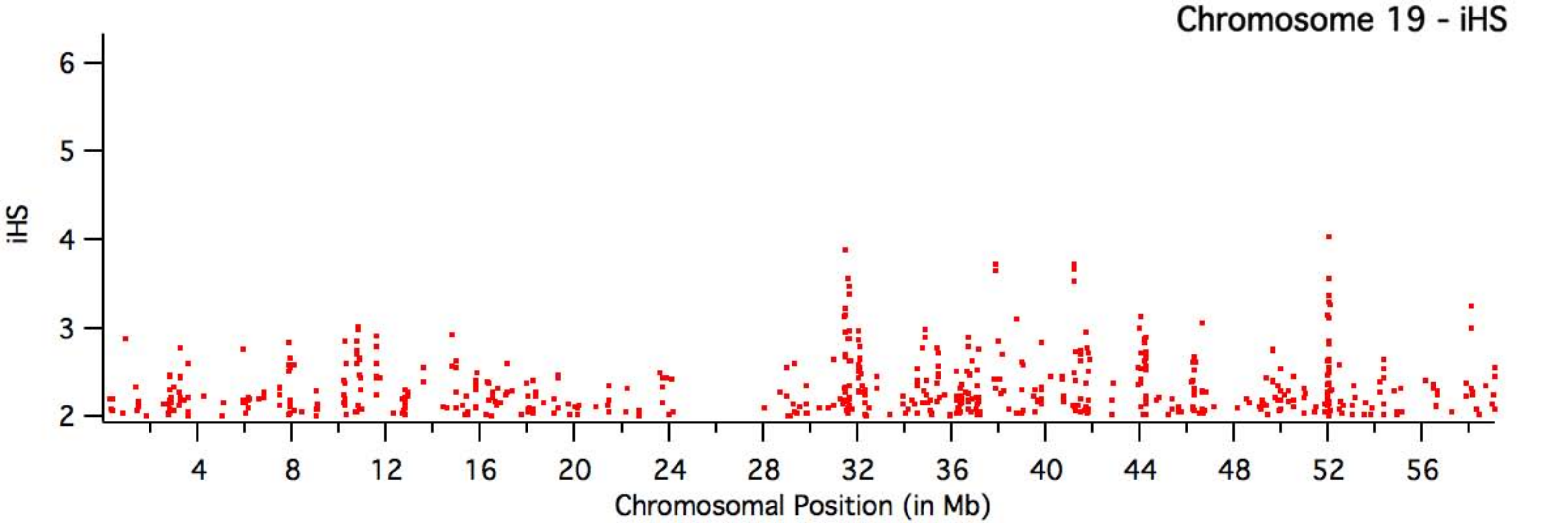
Chromosome 19 - iHS
iHS
6
5
4
3
2
4
8
12
16
20
24
28
32
36
40
44
48
52
56
Chromosomal Position (in Mb)

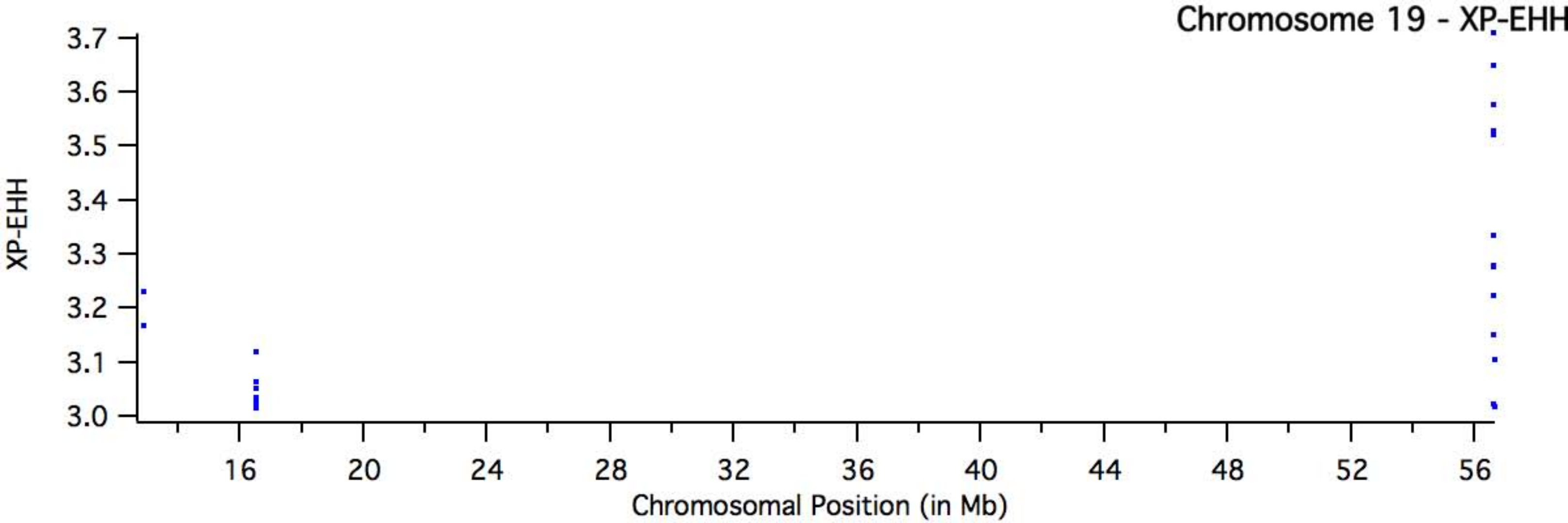

Chromosome 19 - XP-EHH
XP-EHH
3.7
3.6
3.5
3.4
3.3
3.2
3.1
3.0
16
20
24
28
32
36
40
44
48
52
56
Chromosomal Position (in Mb)

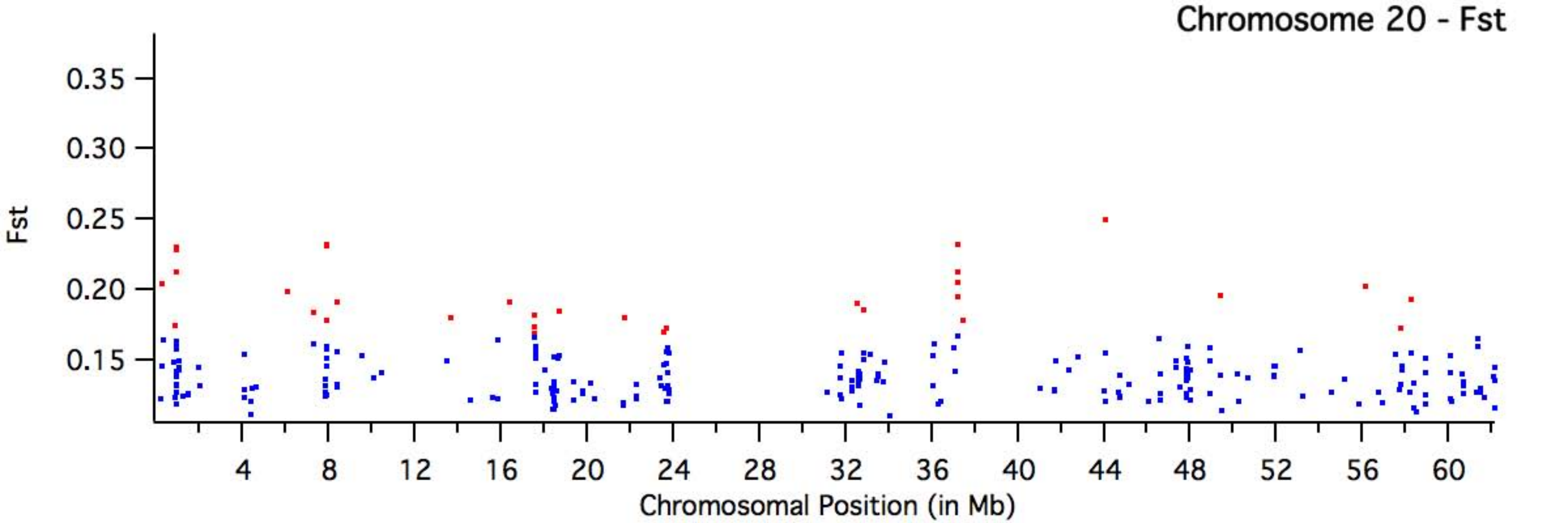
Chromosome 20 - Fst
Fst
0.35
0.30
0.25
0.20
0.15
4
8
12
16
20
24
28
32
36
40
44
48
52
56
60
Chromosomal Position (in Mb)

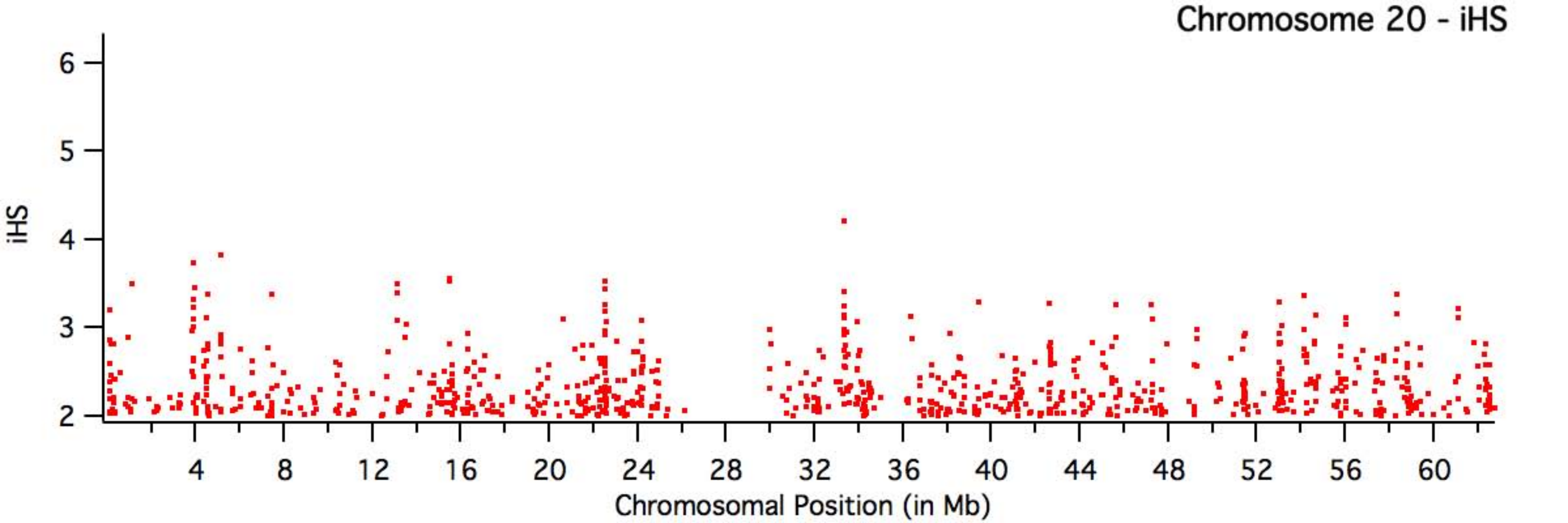

Chromosome 20 - iHS
iHS
6
5
4
3
2
4
8
12
16
20
24
28
32
36
40
44
48
52
56
60
Chromosomal Position (in Mb)

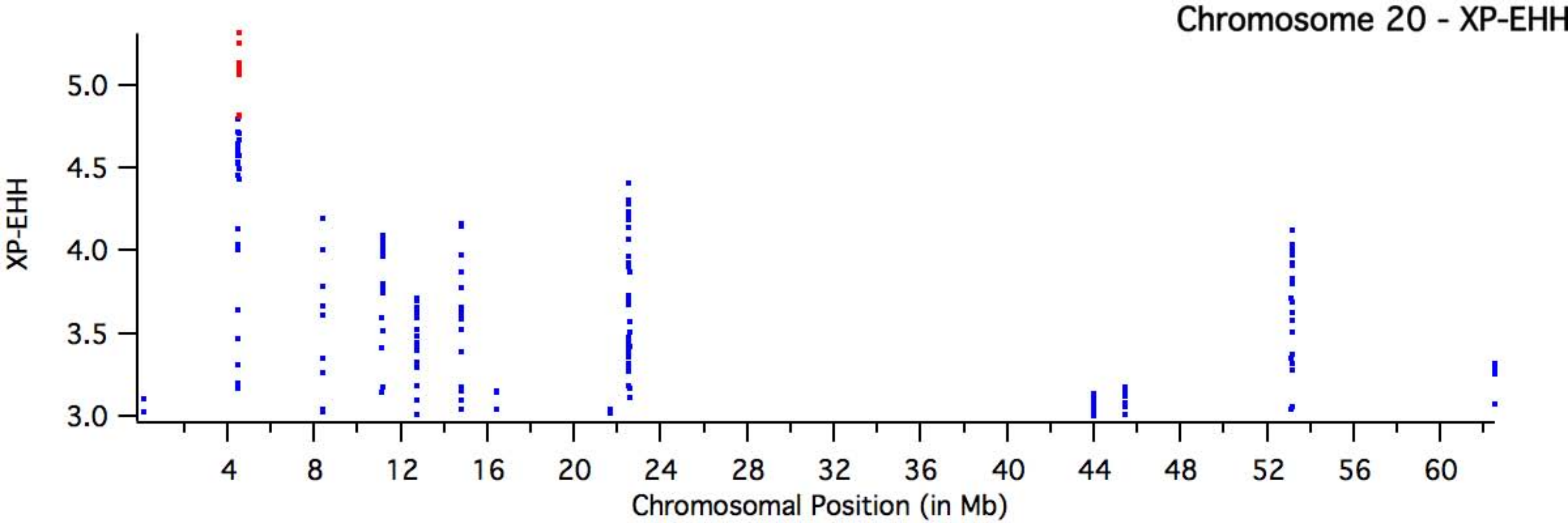
Chromosome 20 - XP-EHH
XP-EHH
5.0
4.5
4.0
3.5
3.0
4
8
12
16
20
24
28
32
36
40
44
48
52
56
60
Chromosomal Position (in Mb)

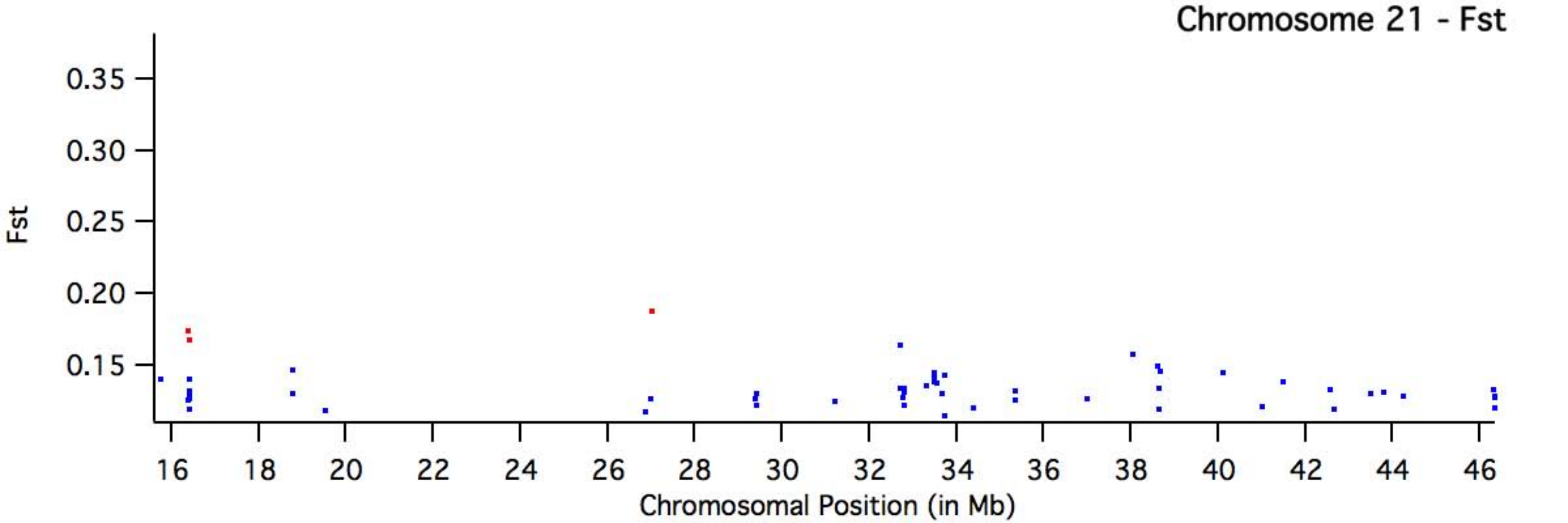
Chromosome 21 - Fst
Fst
0.35
0.30
0.25
0.20
0.15
16
18
20
22
24
26
28
30
32
34
36
38
40
42
44
46
Chromosomal Position (in Mb)

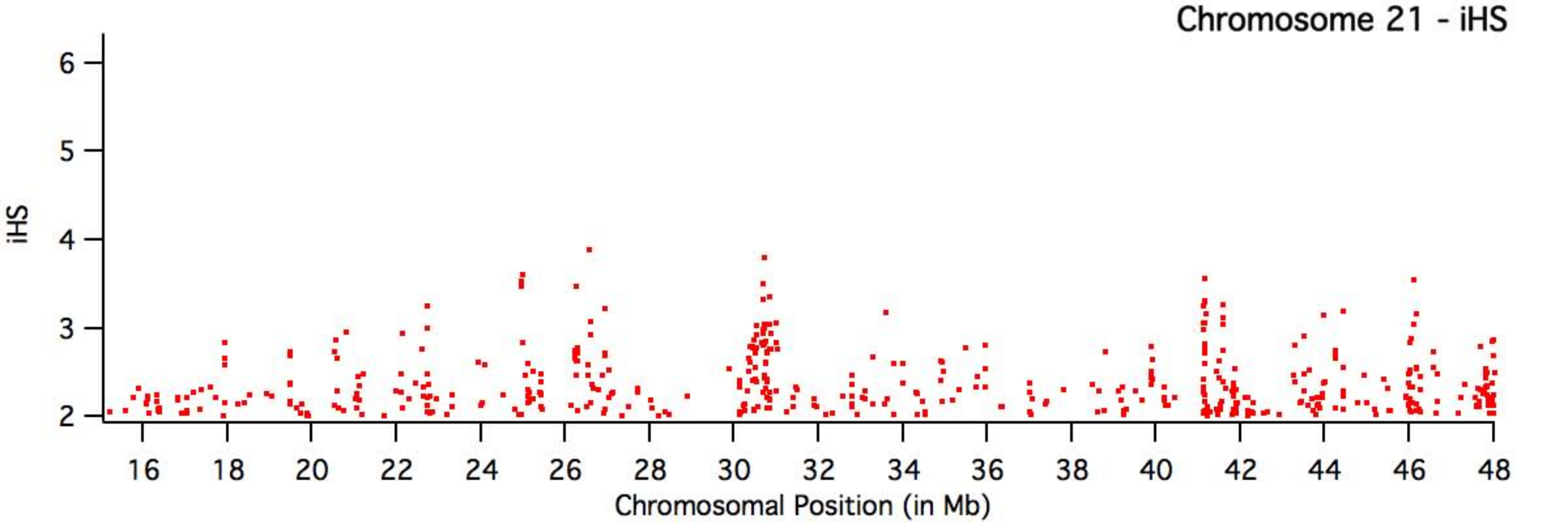
Chromosome 21 - iHS
iHS
2
3
4
5
6
16
18
20
22
24
26
28
30
32
34
36
38
40
42
44
46
48
Chromosomal Position (in Mb)

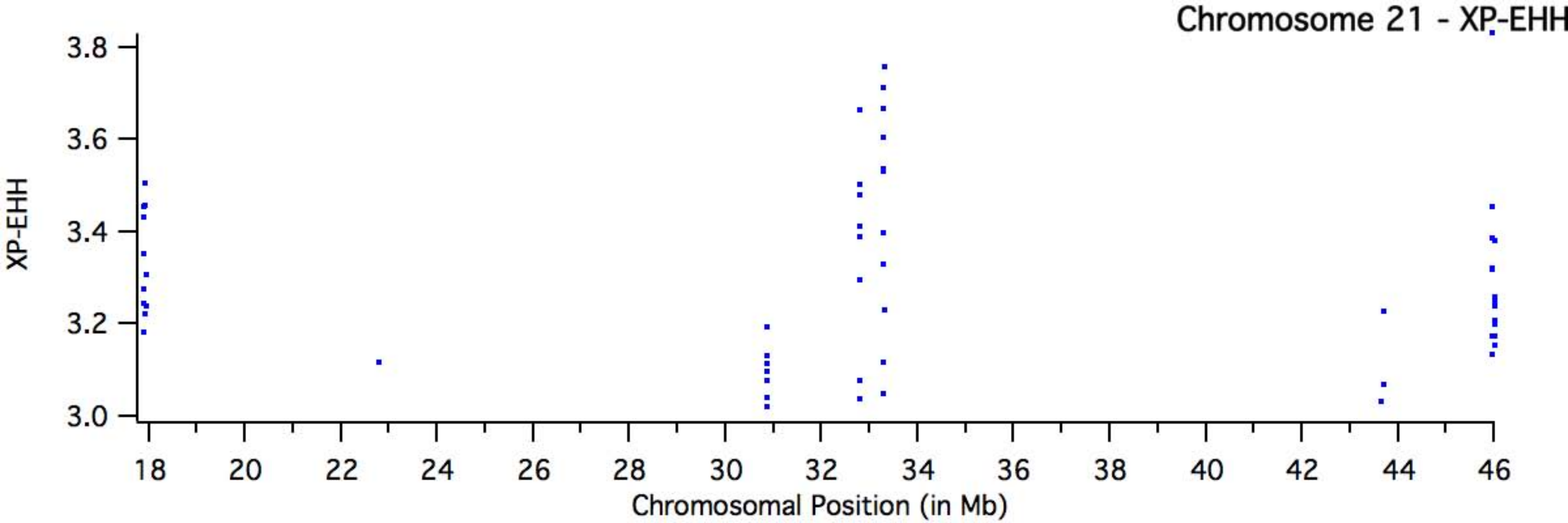
Chromosome 21 - XP-EHH
XP-EHH
3.0
3.2
3.4
3.6
3.8
18
20
22
24
26
28
30
32
34
36
38
40
42
44
46
Chromosomal Position (in Mb)

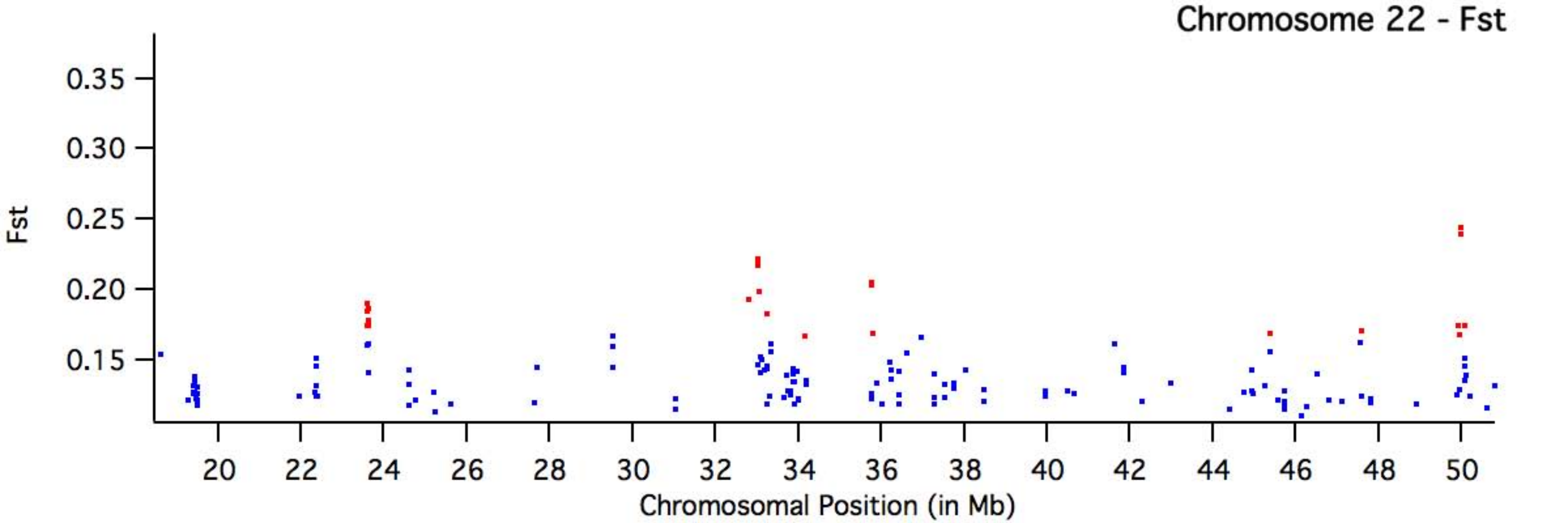
Chromosome 22 - Fst
Fst
0.35
0.30
0.25
0.20
0.15
20
22
24
26
28
30
32
34
36
38
40
42
44
46
48
50
Chromosomal Position (in Mb)

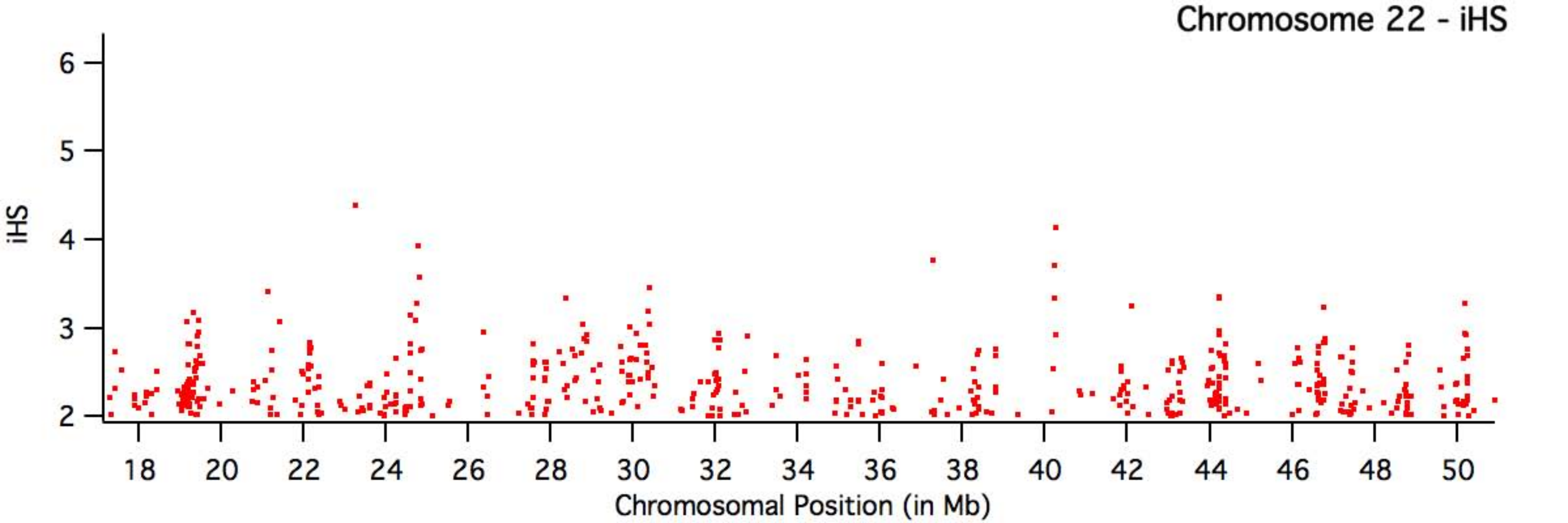

Chromosome 22 - iHS
iHS
6
5
4
3
2
18
20
22
24
26
28
30
32
34
36
38
40
42
44
46
48
50
Chromosomal Position (in Mb)

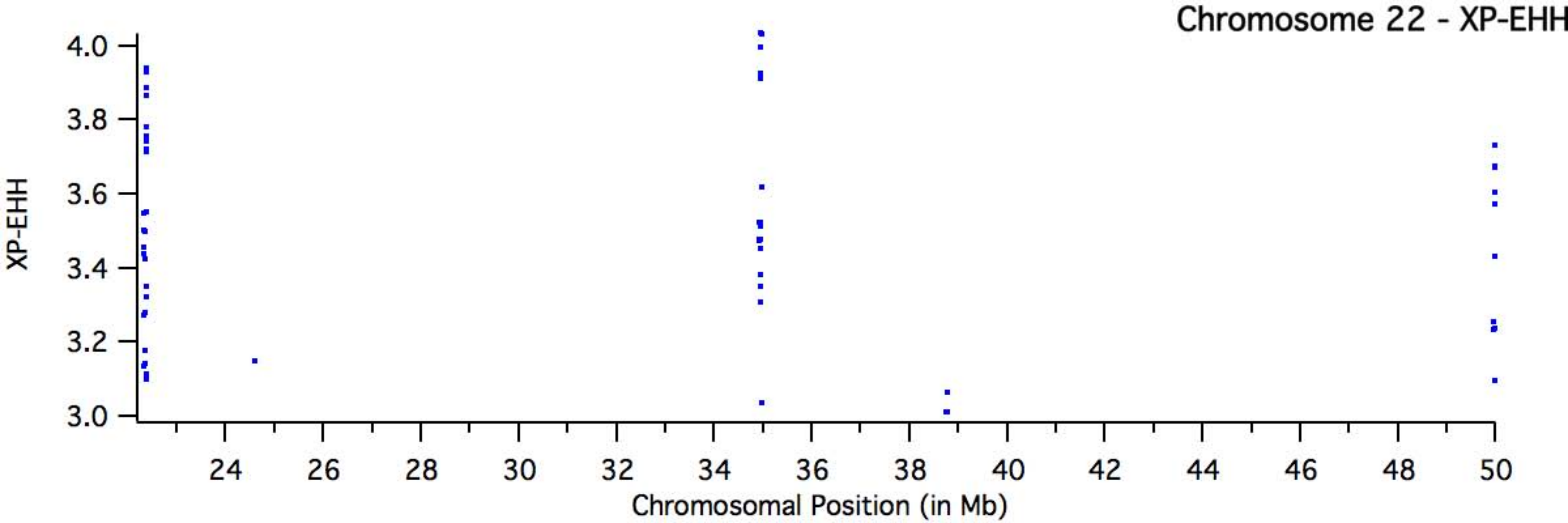

Chromosome 22 - XP-EHH
XP-EHH
4.0
3.8
3.6
3.4
3.2
3.0
24
26
28
30
32
34
36
38
40
42
44
46
48
50
Chromosomal Position (in Mb)
